# Dynamic changes in the gut microbiota during three consecutive trimesters of pregnancy and their correlation with abnormal glucose and lipid metabolism

**DOI:** 10.1186/s40001-024-01702-0

**Published:** 2024-02-12

**Authors:** Yiyang Gao, Jinjia Zhang, Haiying Chen, Xiaohui Jin, Zhenyu Lin, Chenling Fan, Zhongyan Shan, Weiping Teng, Jing Li

**Affiliations:** 1 Department of Endocrinology and Metabolism, Institute of Endocrinology, NHC Key Laboratory of Diagnosis and Treatment of Thyroid Diseases, Liaoning Provincial Key Laboratory of Endocrine Diseases, The First Hospital of China Medical University, Shenyang, 110001 People’s Republic of China; 2 Department of Obstetrics and Gynecology, The First Hospital of China Medical University, Shenyang, China

**Keywords:** Pregnancy, Gut microbiota, Glucose and lipid metabolism

## Abstract

**Introduction:**

During normal pregnancy, changes in the gut microbiota (GM) in response to physiological alterations in hormonal secretion, immune functions and homeostasis have received extensive attention. However, the dynamic changes in the GM during three consecutive trimesters of pregnancy and their relationship with glucose and lipid metabolism have not been reported. In this study, we aimed to investigate the dynamic changes in the diversity and species of the GM during three consecutive trimesters in women who naturally conceived, and their relationships with abnormal fasting blood glucose (FBG) and serum lipid levels.

**Methods:**

A total of 30 pregnant women without any known chronic or autoimmune inflammatory disease history before pregnancy were enrolled during the first trimester. Serum and stool samples were collected during the first trimester, the second trimester, and the third trimester. Serum samples were tested for FBG and blood lipid levels, and stool specimens were analyzed by 16S rDNA sequencing.

**Results:**

The abundance ratio of bacteroidetes/firmicutes showed an increasing tendency in most of the subjects (19/30, 63.3%) from the first to the third trimester. LEfSe analysis showed that the abundance of *Bilophila* was significantly increased from the first to the third trimester. In addition, at the genus level, the increased relative abundance of *Mitsuokella*, *Clostridium *sensu stricto and *Weissella* were potentially involved in the development of high FBG during pregnancy. The raised relative abundance of *Corynebacterium*, *Rothia* and *Granulicatella* potentially contributed to the occurrence of dyslipidemia during pregnancy.

**Conclusions:**

There are dynamic changes in the GM during the three trimesters, and the alterations in some bacterium abundance may contribute to the development of high FBG and dyslipidemia during pregnancy. Monitoring enterotypes and correcting dysbiosis in the first trimester may become new strategies for predicting and preventing glucolipid metabolism disorders during pregnancy.

**Supplementary Information:**

The online version contains supplementary material available at 10.1186/s40001-024-01702-0.

## Background

The human intestine is colonized by more than 10^14^ bacteria and various types of phages, viruses, etc., collectively known as the gut microbiota (GM). The composition and activity of these bacteria depend on the pathophysiological status of the host and are subject to complex interactions depending on the host’s genome, nutrition and lifestyle [1]. The GM is engaged in numerous physiological and metabolic activities in humans, including regulating immunological function, maintaining the intestinal environment, and controlling gastrointestinal motility [2, 3]. Although it is still debated whether the fetal and prenatal intrauterine environment is stably colonized by microbial communities in healthy pregnancies [4], metabolites or maternal immunological responses may indirectly expose babies to microbes and affect the environment for development [5].

Changes in the GM are thought to be linked to immunological and physiological adaptations during pregnancy [6]. For normal fetal growth and development, the GM was observed to undergo a gradual shift toward microbes involved in energy production and accumulation, which appear to contribute to maternal weight gain and insulin resistance and may affect maternal glucolipid metabolism and fetal growth [7–9]. In addition, the changes in the GM that occur during pregnancy may influence energy metabolism and accumulation, which may affect maternal glucose and lipid metabolism and fetal growth and development. Several studies have investigated the changes that occur in the GM during the first trimester (T1), the second trimester (T2) or the third trimester (T3) [9–12], and some of them have looked into the link between GM alterations in T1 or T2 and glucose and lipid metabolism [9, 13, 14]. Ma et al. [13] recently discovered a link between an increase in *Ruminococcaceae* abundance during T1 and the subsequent development of gestational diabetes (GD) at 20–24 gestational weeks. Hu et al. [14] demonstrated that the majority of *Enterobacteriaceae*, *Ruminococcaceae spp.*, and *Veillonellaceae spp.* in T1 had a substantial correlation with the incidence of GD during the late second trimester. In a study performed in T2 pregnant women, the increases in the abundance of total bacteria and the number of *staphylococci* were related to an increase in plasma cholesterol levels, and an increase in the abundance of *Bacteroides* was related to an increase in folic acid and HDL-C levels [9].

However, not only did prior studies fail to yield consistent results, but the dynamic changes in the distribution of the GM in pregnant women over the course of three trimesters and their relationship to glucose and lipid metabolism have yet to be described. This work focused on the dynamic changes in the GM in pregnant women over three consecutive trimesters and attempted to determine their relationship with abnormal glucose and lipid metabolism.

## Materials and methods

### Participants

This prospective research was performed based on the maternal–fetal cohort study in Northeast China (MFCNC) and was approved by the Ethics Committee of the First Hospital of China Medical University (No. 2018-22-3). Participants were enrolled at the Obstetrics Clinic of the First Hospital of China Medical University from January 2018 to September 2019, and all of them signed informed consent forms. The following conditions had to be met for inclusion: (1) females aged 20–35 years, (2) at least 1 year of residence in the northeastern region of China, and (3) natural conception and in T1 (7–13 weeks). The exclusion criteria were as follows: (1) thyroid dysfunction or thyroid autoantibody positivity, (2) body mass index (BMI) < 18.0 kg/m^2^ or ≥ 28.0 kg/m^2^ before pregnancy, (3) smoking and drinking histories, (4) use of antibiotics and probiotics within 3 months at the time of enrollment, (5) any known chronic or autoimmune inflammatory disease history before pregnancy, (6) lack of blood or fecal specimens, and (7) lack of follow-up information. A total of 30 women in T1 were ultimately recruited. Serum and fecal samples were collected consecutively at T1, T2 (14–27 weeks), and T3 (28–33 weeks).

### Demographic information collection

The pregnant women were given questionnaires at the time of enrollment. The questionnaires included the following items: (1) age, ethnicity, education level, pre-pregnancy weight, and height; (2) reproductive history, history of disease and surgery, family history, etc.; and (3) pre-pregnancy smoking and alcohol intake history. Parts of the questionnaire that contain the information used in this study are given in Additional file 1.

### Serum sample collection and tests

Fasting serum samples were collected consecutively in each of the three trimesters. All subjects fasted overnight for 8–12 h, and approximately 5 ml of peripheral venous blood was collected between 7:30 and 9:30 am. All the samples were stored at 4 °C immediately after collection, and then centrifugation was performed at 3800 rpm for 10 min to separate the serum. Serum samples were assayed for FBG, triglyceride (TG), total cholesterol (TC), high-density lipoprotein cholesterol (HDL-C), and low-density lipoprotein cholesterol (LDL-C) levels. Thyroid functions including free thyroxine (FT4) and thyroid stimulating hormone (TSH), and thyroid autoantibodies including serum thyroid peroxidase antibody (TPOAb) and thyroglobulin antibodies (TgAb) were examined during T1 to have those women with thyroid dysfunctions excluded from this study.

FBG and blood lipid (TG, TC, HDL-C, and LDL-C) levels were measured by a Roche COBAS8000 C702 automatic biochemical immunoassay analyzer (Roche Diagnosis, Switzerland). FBG levels were measured by the hexokinase method, TG levels were measured by the enzymatic method, TC levels were measured by the cholesterol oxidase method, and HDL-C and LDL-C levels were measured by the enzyme colorimetric method. Tests for thyroid function and thyroid antibodies, including FT4, TSH, TPOAb and TgAb, were performed with electrochemiluminescence immunoassay using the Roche Cobas Elesys 601 Kit (Roche Diagnosis, Switzerland).

Based on the American Diabetes Association (ADA) standards, the cutoff value for increased FBG in pregnant women is 5.10 mmol/L [15]. In addition, hypertriglyceridemia was defined as TG levels ≥ 1.7 mmol/L (150 mg/dL), hypercholesterolemia as TC levels ≥ 5.2 mmol/L (200 mg/dL), hyper-low-density lipoprotein cholesterolemia as LDL-C levels ≥ 3.4 mmol/L (130 mg/dL), and hypo-high-density lipoprotein cholesterolemia as HDL-C levels < 1.3 mmol/L (50 mg/dL) [16]. Any abnormality in the levels of blood lipids (TG, TC, HDL-C, and LDL-C) was diagnosed as dyslipidemia. According to the American Thyroid Association (ATA) guideline, trimester-specific reference ranges should be applied for assessing thyroid functions in pregnant women [17]. Based on trimester-specific reference intervals previously established by our group using electrochemiluminescence immunoassay (Roche Diagnosis, Switzerland) [18], euthyroidism in T1 was defined as 13.35 ≤ FT4 ≤ 19.01 pmol/L and 0.35 ≤ TSH ≤ 4.13 mIU/L. Subjects with thyroid dysfunction were excluded from this study. In addition, based on the reference range from the manufacturer, those with positivity for thyroid autoantibodies (TPOAb > 34 IU/mL or TgAb > 115 IU/mL) in the serum were also excluded.

### Fecal sample collection and 16S rDNA sequencing

Fresh fasting fecal samples were collected in each of the three consecutive trimesters by PSP® Spin Stool DNA Plus Kit (Invitek, Berlin, German) containing DNA stabilizer. They were then immediately stored at − 80 °C until all specimens were available for 16S rDNA sequencing. Temperature control was used during shipping of the samples.

In brief, the QIAamp Fast DNA Stool Mini Kit (Qiagen, Hilden, Germany) was used to extract fecal genomic DNA in accordance with the manufacturer's instructions. The V3–V4 region of the 16S rDNA gene was amplified through polymerase chain reaction (PCR) with sample DNA as a template and 341F and 806R as primers. A KAPA HiFi Hotstart ReadyMix PCR kit (KAPA Biosystems, UK) was used to conduct PCR. Following the manufacturer's recommendations, amplicons were extracted from 2% agarose gels, purified with the AxyPrep DNA Gel Extraction Kit (Axygen Biosciences, Union City, CA, USA), and quantified with Qubit®20 (Invitrogen, USA). The MiSeq platform PE250 strategy (Illumina, Inc., CA, USA) was used for paired-end sequencing, and the overlapping relationship was used for splicing to obtain long reads in the hypervariable region. The remaining lengths and average base quality of the assembled tags were tested after barcode and primer sequences were trimmed. The average Phred score of bases was no worse than 20 (Q20), with no more than three ambiguous N. 16S tags were confined between 220 and 500 bp. The number of copies of tags was counted, and duplicate tags were eliminated. Only tags with a frequency greater than one were grouped into operational taxonomic units (OTUs), each with a representative tag. Using UPARSE (http://drive5.com/uparse/), the OTUs were clustered with 97% similarity. Chimeric sequences were identified and removed using Usearch (version 7.0.1090). RDP Classifier (http://rdp.cme.msu.edu/) was used to assign each representative tag to a taxon using a confidence threshold of 0.8 [19, 20]. DNA extraction, library construction and sequencing were conducted at Realbio Genomics Institute (Shanghai, China) [21]. Nucleic acid sequences are available at the Sequence Read Archive (SRA) under accession number SRP429877 at http://ncbi.nlm.nih.gov.

### Statistical analysis

The demographic and serological data were processed and analyzed using IBM SPSS 23.0 (SPSS Inc., Chicago, IL, USA). Categorical variables were given as numbers (percentages), normally distributed variables were given as the mean ± standard deviation, and skewed variables were given as the median (25–75th percentiles). Furthermore, the Chi-square test, independent sample *t* test, one-way ANOVA, Mann‒Whitney *U* test, Kruskal‒Wallis test, Wilcoxon signed-rank test, paired *t* test and Friedman’s rank test were adopted for statistical analysis when appropriate.

QIIME (V1.9.1) and R (V3.5.1) were mainly used for the sequencing and statistical analysis of the GM. QIIME was used to construct an OTU profiling table and perform alpha and beta diversity analysis. The Chao1 index of the alpha diversity index was used to gauge sequencing depth. Alpha diversity analysis, including assessments of Chao1, Simpson's and Shannon's indices, was performed to reflect the microbial community richness. Comparing the diversity of the microbial community between samples was performed using principal coordinates analysis (PCoA) of beta diversity analysis. The differences in the species or communities between groups were determined by linear discriminant analysis effect size (LEfSe). The LEfSe results were assessed by linear discrimination analysis (LDA), which was performed by LEfSe Tools, with an LDA score cutoff of 2. The differences in alpha diversity and LEfSe between groups were analyzed by rank sum test analysis. The Wilcoxon test in R was used for comparing two groups of samples, and the Kruskal test in R was used for comparing more than two groups of samples. Differences in all the statistical analyses were considered significant when the *P* value < 0.05 or the adjusted cutoff *P* value was due to multiple comparisons in the rank sum test and Chi-square test.

## Results

### Description of the study cohort

This study included 30 pregnant women who had no known history of chronic or autoimmune inflammatory diseases. According to the dynamic changes in FBG levels during the three trimesters, they were assigned into the following 4 groups: ① normal FBG levels during the whole pregnancy (WNG, *n* = 15), ② normal FBG levels in T1 and high FBG levels in T2 and/or T3 (ENG, *n* = 8), ③ normal FBG levels in T3 and high FBG levels in T1 and/or T2 (LNG, *n* = 5) and ④ high FBG levels during the whole pregnancy (WHG, *n* = 4). Since there were 2 subjects with high FBG levels only at T2 who were included in both the ENG and LNG groups, we did not perform any comparison analysis between the two groups. According to the diagnostic criteria for GD proposed by the International Association of the Diabetes and Pregnancy Study Groups (IADPSG) [22], all those women in the ENG, LNG and WHG groups had GD. Based on differences in serum lipid levels, the subjects were assigned into the following 3 groups: ① dyslipidemia starting from T1 of pregnancy (DL1, *n* = 6), ② dyslipidemia since T2 (DL2, *n* = 16) and ③ dyslipidemia only in T3 (DL3, *n* = 8). The WHG group was older than the ENG and LNG groups, and the WNG group was older than the LNG group (Table 1). There was one pre-gestational overweight woman in the WHG group (BMI: 25.80 kg/m^2^); however, no significant difference was found in BMI between groups. As shown in Table 1, the ENG group exhibited a significant increase in FBG level from T1 to T2, and the LNG group showed a significant decrease in FBG level in T3 as compared with that in T1. In addition, the serum levels of TG, TC, LDL-C and dyslipidemia prevalence exhibited gradual increases with the progression of pregnancy among those women (Table 2). However, as shown in Table 2, there was no significant difference between the DL1, DL2 and DL3 groups except for the lipid levels.

**Table 1:** Characteristics of the pregnant women included in this study stratified by FBG level in the three trimesters

	Total subjects (*n* = 30)	WNG (*n* = 15)	ENG (*n* = 8)	*P*1^d^	*P*2^e^	LNG(*n* = 5)	*P*1^d^	*P*2^e^	WHG (*n* = 4)	*P*1^d^
Age, years^a^	30 (28 ~ 30.25)	30 (28 ~ 31)	28.5 (28 ~ 29.75)	0.19	**0.03**	28 (25 ~ 28)	**0.01**	**0.02**	30.5 (30 ~ 32.5)	0.36
College and above education^b^	29 (96.67)	15 (100)	8 (100)	1.00	0.14	5 (100)	1.00	0.24	3 (75.00)	**0.047**
Han nationality^b^	28 (93.30)	14 (93.33)	8 (100)	0.64	0.14	5 (100)	0.55	0.24	3 (75.00)	0.29
BMI before pregnancy, kg/m^2c^	21.29 ± 1.93	21.56 ± 1.58	20.73 ± 1.49	0.88	0.13	20.40 ± 1.67	0.18	0.17	22.90 ± 3.14	0.24
History of tea drinking^b^	0 (0)	0 (0)	0 (0)	1.00	1.00	0 (0)	1.00	1.00	0 (0)	1.00
History of coffee drinking^b^	2 (6.67)	1 (6.67)	1(12.5)	0.64	0.46	0 (0)	0.55	1.00	0 (0)	0.60
Family history of GD ^b^	0 (0)	0 (0)	0 (0)	1.00	1.00	0 (0)	1.00	1.00	0 (0)	1.00
Family history of diabetes^b^	1 (3.33)	0 (0)	0 (0)	1.00	1.00	1 (20.00)	0.08	0.34	0 (0)	1.00
Gestational age when the serum samples collected^a^										
T1	10 (8–12)	9 (7–12)	10 (9–12)	0.47	1.00	12 (7.5–12.5)	0.56	0.80	10.5 (7.75–12.5)	0.69
T2	18 (17–20)	18 (18–21)	18 (17–20.25)	0.44	0.43	19 (17–20)	0.59	0.80	19 (17.5–22)	0.72
T3	30 (28.25–32)	31 (28–33)	29 (28.25–31.5)	0.26	0.93	32 (28.5–33)	0.72	0.32	29.5 (28.25–30)	0.13
FBG, mmol/L^c^										
T1	4.87 ± 0.49	4.59 ± 0.34	4.82 ± 0.18	0.10	** < 0.01**	5.17 ± 0.50	** < 0.01**	0.25	5.58 ± 0.46	** < 0.01**
T2	5.01 ± 0.60	4.61 ± 0.28	5.63 ± 0.73	** < 0.01**	0.31	5.34 ± 0.56	** < 0.01**	0.71	5.23 ± 0.15	** < 0.01**
T3	4.91 ± 0.59	4.55 ± 0.30	5.42 ± 0.74	** < 0.01**	0.80	4.71 ± 0.23	0.28	** < 0.01**	5.33 ± 0.13	** < 0.01**
*P*	T1 *vs.* T2:0.27	T1 *vs.* T2: 0.87	**T1 *vs.* T2: 0.03**			T1 *vs.* T2: 0.71			T1 *vs.* T2: 0.26	
	T1 *vs.* T3:0.73	T1 *vs.* T3: 0.60	T1 *vs.* T3: 0.07			**T1 *vs.* T3: 0.049**			T1 *vs.* T3: 0.43	
	T2 *vs.* T3:0.21	T2 *vs.* T3: 0.21	T2 *vs.* T3: 0.48			T2 *vs.* T3: 0.12			T2 *vs.* T3: 0.32	
B/F^a^										
T1	0.99 (0.56 ~ 1.37)	1.03 (0.64 ~ 1.52)	1.16 (0.57 ~ 1.43)	0.68	0.93	0.51 (0.40 ~ 1.70)	0.35	0.73	0.82 (0.55 ~ 1.31)	0.60
T2	1.02 (0.83 ~ 1.46)	1.30 (0.84 ~ 1.48)	0.93 (0.76 ~ 1.12)	0.17	0.11	1.02 (0.82 ~ 1.48)	1.00	0.91	1.21 (0.99 ~ 1.87)	0.74
T3	1.20 (0.90 ~ 1.71)	1.20 (0.84 ~ 1.51)	1.19 (0.81 ~ 2.18)	0.98	0.68	0.90 (0.62 ~ 2.10)	0.55	0.41	1.45 (1.06 ~ 1.81)	0.53
*P*	0.09	0.34	1.00			0.17			0.37	
Dominant GM, Bacteroidetes ^b^										
T1	15 (50.00)	8 (53.33)	5 (62.50)	0.67	0.22	2 (40.00)	0.61	0.64	1 (25.00)	0.31
T2	17 (56.67)	9 (60.00)	4 (50.00)	0.65	0.41	3 (60.00)	1.00	0.64	3 (75.00)	0.58
T3	20 (66.67)	10 (66.67)	5 (62.50)	0.84	0.41	2 (40.00)	0.29	0.64	4 (100)	0.18
*P*	0.43	0.76	0.84			0.77			0.07	

*P* < 0.05 are designated in bold

BMI: body mass index, GD: gestational diabetes, FBG: fasting blood glucose, T1: the first trimester, T2: the second trimester, T3: the third trimester, GM: gut microbiota, B/F: the abundance ratio of Bacteroidetes/Firmicutes, WNG: normal FBG during the whole pregnancy, ENG: normal FBG in the early stage (T1) of pregnancy, LNG: normal FBG in the late stage (T3) of pregnancy, WHG: high FBG during the whole pregnancy

^a^non-normal distributed continuous variables were expressed as median with interquartile ranges (*Q*1 ~ *Q*3), the difference between two groups was analyzed using Mann–Whitney U test, and paired Friedman nonparametric test was performed for self-comparisons among the three stages

^b^Categorical variables were expressed using the number of cases and composition ratio [*n* (%)], and the differences between groups were analyzed using the Chi-square test

^c^Normal distributed continuous variables were reported as mean ± standard deviation and the difference between two groups was analyzed using the independent sample *t* test, and the changes among multiple groups were analyzed using paired *t* test for pairwise comparisons

^d^*P1* is the *P* value of each group compared to the WNG group

^e^*P2* is the *P* value of each group compared to the WHG group

**Table 2:** Characteristics of the pregnant women included in this study stratified by serum lipid levels in the three trimesters

	Total subjects (*n* = 30)	DL3 (*n* = 8)	DL2(*n* = 16)	DL1 (*n* = 6)	*P*
Age, Years^a^	–	28 (25.25 ~ 30.75)	30 (28 ~ 30.75)	30 (29.75 ~ 30.25)	0.30
College and above education^b^	–	8 (100)	15 (93.75)	6 (100)	0.64
Han nationality	–	8 (95.65)	15 (93.75)	5 (85.71)	0.46
BMI before pregnancy, kg/m^2c^	–	21.36 ± 2.08	21.07 ± 1.44	21.07 ± 1.44	0.48
History of tea drinking ^b^	–	0 (0)	0 (0)	0 (0)	1.00
History of coffee drinking ^b^	–	0 (0)	1 (6.25)	1 (16.67)	0.46
Family history of GD ^b^	–	0 (0)	0 (0)	0 (0)	1.00
Family history of diabetes ^b^	–	1 (12.50)	0 (0)	0 (0)	0.24
Gestational age when the serum samples collected ^a^					
T1	–	9.5 (8.25–11.75)	11 (8–12.75)	8.5 (7–10.5)	0.335
T2	–	19 (17–19.75)	18 (17.25–18.75)	21 (19.25–23.25)	0.051
T3	–	29.5 (28.25–32)	30.5 (28.25–32)	31 (29.5–32.25)	0.798
TG, mmol/L ^c^					
T1	1.13 ± 0.42	0.92 ± 0.33 (0.51–1.47) ^d^	1.13 ± 0.38 (0.46–1.66) ^d^	1.39 ± 0.53 (0.83–2.03) ^d^	0.11
T2	1.79 ± 0.67	1.13 ± 0.21 (0.77–1.06) ^d^	1.89 ± 0.51 (1.12–3.01) ^d^	2.41 ± 0.78 (1.54–3.39) ^d^	** < 0.01 (DL3 *vs.* DL2: < 0.01; DL3 *vs.* DL1: < 0.01)**
T3	2.51 ± 0.79	2.25 ± 0.61 (1.66–3.36) ^d^	2.47 ± 0.81 (1.43–4.75) ^d^	2.95 ± 0.88 (2.16–4.30) ^d^	0.26
*P*	**T1 *vs.* T2: < 0.01**	T1 *vs.* T2: 0.24	**T1 *vs.* T2: < 0.01**	**T1 *vs.* T2: 0.01**	
	**T1 *vs.* T3: < 0.01**	**T1** ***vs.* T3:** **<** **0.01**	**T1 *vs.* T3: < 0.01**	**T1 *vs.* T3: 0.01**	
	**T2 *vs.* T3: < 0.01**	**T2** ***vs.*** **T3:** **<** **0.01**	**T2 *vs.* T3: 0.02**	T2 *vs.* T3: 0.06	
TC, mmol/L^c^					
T1	4.24 ± 0.70	3.78 ± 0.61 (2.75–4.69) ^d^	4.36 ± 0.53 (2.87–5.08) ^d^	4.54 ± 1.05 (3.09–6.06) ^d^	0.09
T2	5.08 ± 0.76	4.36 ± 0.49 (3.58–4.95) ^d^	5.31 ± 0.66 (4.02–6.54) ^d^	5.42 ± 0.74 (4.34–6.26) ^d^	** < 0.01 (DL3 *vs.* DL2: < 0.01, DL3 *vs.* DL1:0.01)**
T3	5.81 ± 0.83	5.44 ± 0.59 (4.35–6.26) ^d^	6.06 ± 0.89 (4.33–7.56) ^d^	5.65 ± 0.85 (4.62–6.70) ^d^	0.20
*P*	**T1 *vs.* T2: < 0.01**	**T1 *vs.* T2: < 0.01**	**T1 *vs.* T2: < 0.01**	**T1 *vs.* T2: 0.04**	
	**T1 *vs.* T3: < 0.01**	**T1 *vs.* T3: < 0.01**	**T1 *vs.* T3: < 0.01**	**T1 *vs.* T3: 0.048**	
	**T2 *vs.* T3: < 0.01**	**T2 *vs.* T3: < 0.01**	**T2 *vs.* T3: < 0.01**	T2 *vs.* T3: 0.14	
HDL-C, mmol/L ^c^					
T1	1.80 ± 0.40	1.74 ± 0.35 (1.32–2.33) ^d^	1.91 ± 0.41 (1.30–2.75) ^d^	1.60 ± 0.43 (1.15–2.17) ^d^	0.24
T2	2.03 ± 0.45	2.07 ± 0.47 (1.55–2.84) ^d^	2.07 ± 0.51 (1.48–3.08) ^d^	1.87 ± 0.22 (1.63–2.19) ^d^	0.64
T3	2.01 ± 0.41	1.99 ± 0.39 (1.54–2.77) ^d^	2.07 ± 0.49 (1.36–2.93) ^d^	1.87 ± 0.18 (1.60–2.05) ^d^	0.60
*P*	**T1 *vs.* T2: < 0.01**	**T1 *vs.* T2: < 0.01**	T1 *vs.* T2: 0.11	T1 *vs.* T2: 0.19	
	**T1 *vs.* T3:0.01**	T1 *vs.* T3: 0.11	T1 *vs.* T3: 0.15	T1 *vs.* T3: 0.24	
	T2 *vs.* T3:0.67	T2 *vs.* T3: 0.54	T2 *vs.* T3: 0.99	T2 *vs.* T3: 0.97	
LDL-C, mmol/L ^c^					
T1	2.40 ± 0.65	2.00 ± 0.66 (0.73–2.99) ^d^	2.44 ± 0.43 (1.66–3.17) ^d^	2.81 ± 0.89 (1.76–3.90) ^d^	0.05
T2	2.86 ± 0.66	2.24 ± 0.59 (0.96–3.01) ^d^	3.06 ± 0.58 (1.93–4.38) ^d^	3.13 ± 0.46 (2.67–3.95) ^d^	** < 0.01 (DL3 *vs.* DL2: < 0.01, DL3 *vs.* DL1:0.02)**
T3	3.42 ± 0.83	2.99 ± 0.64 (1.75–3.85) ^d^	3.73 ± 0.86 (1.67–5.15) ^d^	3.16 ± 0.70 (2.21–3.92) ^d^	0.08
*P*	**T1 *vs.* T2: < 0.01**	**T1 *vs.* T2: 0.03**	**T1 *vs.* T2: < 0.01**	T1 *vs.* T2: 0.33	
	**T1 *vs.* T3: < 0.01**	**T1 *vs.* T3: < 0.01**	**T1 *vs.* T3: < 0.01**	T1 *vs.* T3: 0.49	
	**T2 *vs.* T3: < 0.01**	**T2 *vs.* T3: < 0.01**	**T2 *vs.* T3: < 0.01**	T2 *vs.* T3: 0.92	
B/F^a^					
T1	–	0.91 (0.36 ~ 1.49)	0.97 (0.69 ~ 1.32)	1.09 (0.56 ~ 1.52)	0.87
T2	–	1.10 (0.70 ~ 1.52)	1.09 (0.84 ~ 1.44)	0.96 (0.84 ~ 1.63)	0.98
T3	–	1.16 (0.90 ~ 1.71)	1.16 (1.12 ~ 1.48)	1.24 (1.07 ~ 1.53)	0.97
*P*	–	0.22	0.27	0.84	
Dominant GM, Bacteroidetes^b^					
T1	–	4 (50.00)	8 (50.00)	3 (50.00)	1.00
T2	–	4 (50.00)	10 (62.50)	3 (50.00)	0.79
T3	–	5 (62.50)	10 (62.50)	5 (83.33)	0.63
*P*	–	0.84	0.71	0.39	

*P* < 0.05 are designated in bold

BMI: body mass index, GD: gestational diabetes, FBG: fasting blood glucose, T1: the first trimester, T2: the second trimester, T3: the third trimester, GM: gut microbiota, TG: triglycerides, TC: total cholesterol, HDL-C, high-density cholesterol, LDL-C: low-density cholesterol, B/F: the abundance ratio of Bacteroidetes/Firmicutes, DL3: with dyslipidemia only in T3, DL2: with dyslipidemia since T2, DL1: dyslipidemia starting from T1 of pregnancy

^a^Non-normal distributed continuous variables were expressed as median with interquartile ranges (*Q*1 ~ *Q*3), the difference between two groups was analyzed using Mann–Whitney U test, and paired Friedman nonparametric test was performed for self-comparisons among the three stages

^b^Categorical variables were expressed using the number of cases and composition ratio [*n* (%)] and the differences between groups were analyzed using the Chi-square test

^c^Normal distributed continuous variables were reported as mean ± standard deviation and the differences between two groups was analyzed using the independent sample *t* test, and the differences among multiple groups were analyzed using paired *t* test for pairwise comparisons

^d^The ranges of serum lipid levels including TG, TC, HDL-C, LDL-C, were expressed by minimum to maximum values

### Comparison of the GM composition during the three stages of pregnancy

**Fig. 1:**
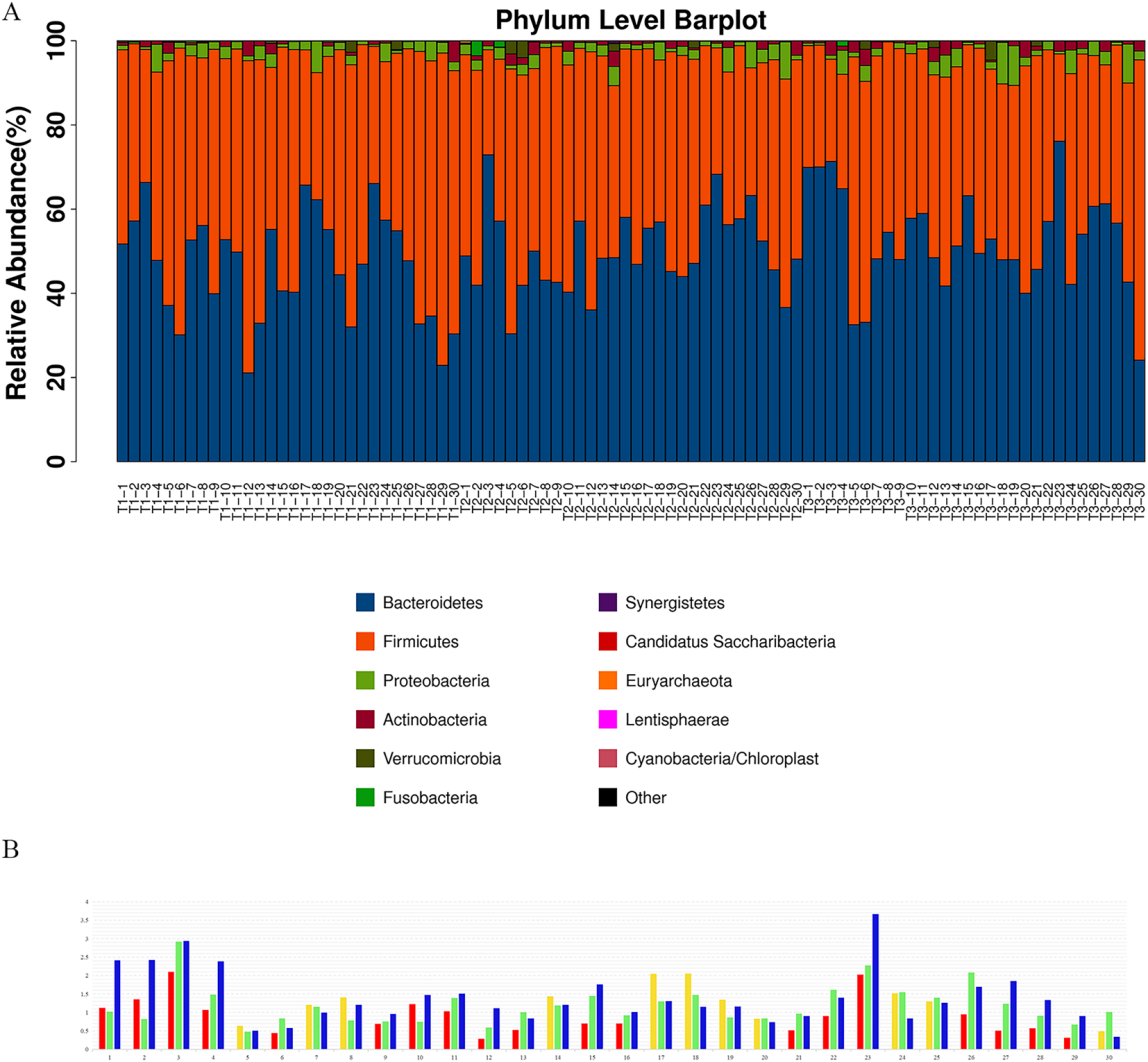
Composition of gut microbiota at phylum level in all subjects studied during the three trimesters of pregnancy. Histogram of gut microbiota (GM) composition in each subject at phylum level during the first trimester (T1), the second trimester (T2) and the third trimester (T3) (**A**); Dynamic change in the abundance ratio of Bacteroidetes/Firmicutes (B/F) in each subject from T1 to T3 (**B**). There was an overall increasing trend in B/F value from T1 (red), T2 (green) to T3 (blue) in 19 women, and no increasing trend from T1 (yellow), T2 (green) to T3 (blue) in the rest 11 women

Pairwise comparisons of the GM were conducted in all subjects enrolled across the three trimesters of pregnancy. Alpha diversity and beta diversity were analyzed to compare the richness and species diversity of the GM among the three trimesters. No significant differences were observed (Additional file 2). In addition, the abundance map from all the subjects showed that Bacteroidetes and Firmicutes were dominant at the phylum level, followed by Proteobacteria and Actinobacteria (Fig. 1A). Bacteroidetes (B) and Firmicutes (F) are the main components of the human GM. The abundance ratio of B/F showed an increasing tendency in most of the subjects (19/30, 63.3%) from T1 to T3 (Fig. 1B). Those subjects were then divided into the B (B/F > 1) and F (B/F < 1) groups in each of the three trimesters. The B/F value significantly increased from T1 to T2 only in the subjects with B/F < 1 in T1, but it did not change from T2 to T3 [B/F: 0.57 (0.48 ~ 0.70) in T1, 0.92 (0.76 ~ 1.23) in T2, 0.96 (0.74 ~ 1.40) in T3; T2 *vs.* T1: *P* < 0.01, T3 *vs.* T1: *P* < 0.01, T3 *vs.* T2: *P* = 0.69]. There were no significant differences in demographic information, FBG, serum lipid levels or the incidence rates of high FBG and dyslipidemia between the B and F groups in each trimester.

**Fig. 2:**
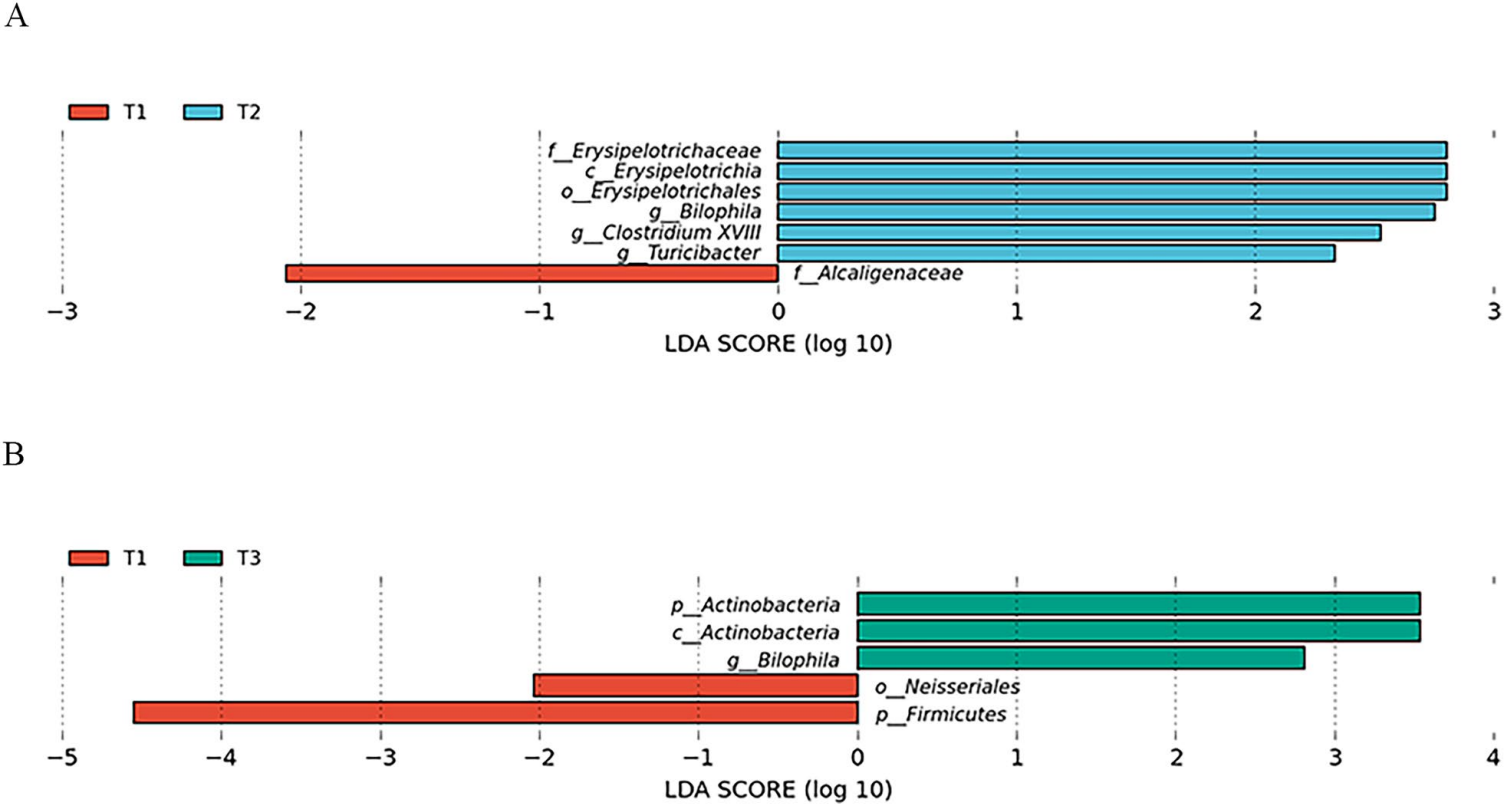
Differentially abundant taxa of gut microbiota in all the subjects across the three trimesters of pregnancy. There were significant differences in the abundance taxa of gut microbiota (GM) by linear discrimination analysis effect size (LEfSe) analysis [linear discrimination analysis (LDA) score > 2.0] between the first trimester (T1) and the second trimester (T2) (**A**), and between T1 and the third trimester (T3) (**B**)

LEfSe analysis was carried out to observe the dynamic changes in the abundance of GM across the three trimesters. As compared with that of T1, the abundances of Erysipelotrichales, Erysipelotrichaceae, Erysipelotrichia, *Bilophila*, *Clostridium XVIII*, and *Turicibacter* were significantly increased, while Alcaligenaceae was markedly decreased in T2 (Fig. 2A). In addition, the abundances of the phylum Actinobacteria, clas*s* Actinobacteria and *Bilophila* were significantly higher and those of Neisseriales and Firmicutes were lower in T3 than T1 (Fig. 2B). LEfSe showed no significant change in GM abundance between T2 and T3. These results suggest that throughout the entire pregnancy, the GM in T1 may exert a dominant influence on the regulation of glucose and lipid metabolism.

### Analysis of the relationship between GM variance and dynamic changes in FBG across the three trimesters

**Fig. 3:**
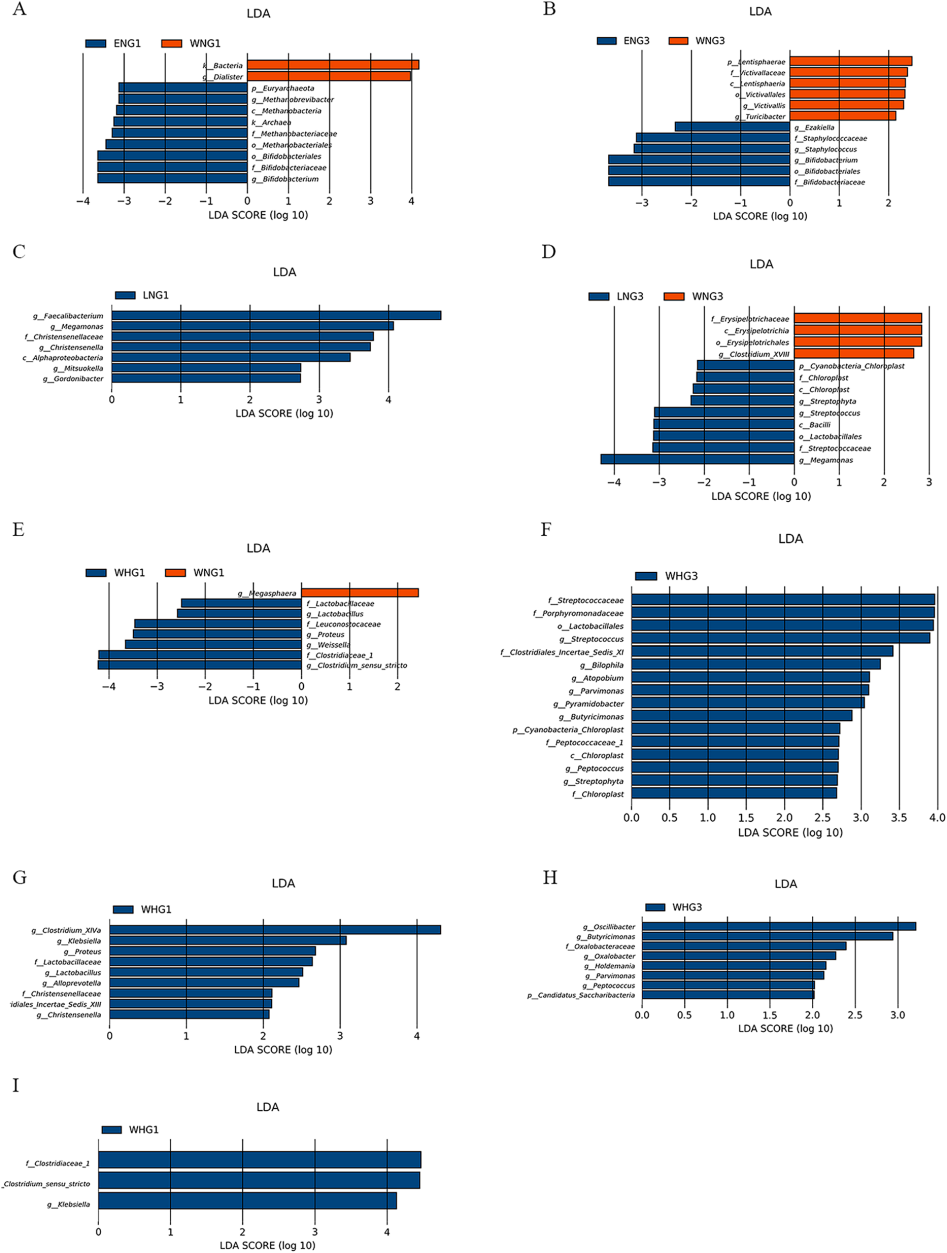
Differentially abundant taxa of gut microbiota with the dynamic change in FBG level across the three trimesters. There were significant differences by linear discrimination analysis effect size (LEfSe) analysis between WNG and ENG groups in the first trimester (T1, **A**) and the third trimester (T3, **B**), between WNG and LNG groups in T1 (**C**) and T3 (**D**), between WNG and WHG groups in T1 (**E**) and T3 (**F**), between WHG and ENG groups in T1 (**G**) and T3 (**H**), between WHG and LNG groups in T1 (**I**). FBG: fasting blood glucose, WNG: normal FBG during the whole pregnancy, ENG: normal FBG in the early stage (T1) of pregnancy, LNG: normal FBG in the late stage (T3) of pregnancy, WHG: high FBG during the whole pregnancy

Analyses were performed to investigate whether GM alterations and FBG fluctuation had potential mutual interaction and whether dysbiosis could induce metabolic disorders. No differences were shown in the alpha or beta diversities among the WNG, ENG, LNG and WHG groups in each trimester (Additional files 3 and 4). LEFSe analysis of GM abundance showed no significant differences among the four groups in T2. At the family and genus levels, the abundances of Bifidobacteriaceae and *Bifidobacterium* were significantly higher in the ENG group than WNG group in both T1 (Fig. 3A) and T3 (Fig. 3B). It indicates that they may promote the later increase in FBG level of the ENG group. In addition, Methanobacteriaceae and *Methanobrevibacter* were more enriched in the ENG group during T1, while the genus *Dialister* decreased in abundance (Fig. 3A). It suggests that the differential distribution of the bacteria may also contribute to the elevated FBG later in pregnancy. The families Staphylococcaceae, Staphylococcus and *Ezakiella* were more enriched in the ENG group during T3, while the families Victivallaceae, *Victivallis* and *Turicibacter* decreased in abundance (Fig. 3B). When compared with that of the WNG group, the abundances of *Faecalibacterium*, *Megamonas*, Christensenellaceae, *Christensenella*, *Gordonibacter*, and *Mitsuokella* showed significant increases in the LNG group during T1, and *Megamonas* was also markedly enriched during T3 (Fig. 3C, D). In addition, Streptococcaceae, Streptococcus, Chloroplast, and *Streptophyta* were mainly enriched during T3 in the LNG group, while Erysipelotrichaceae and *Clostridium_XVIII* were markedly depleted during this period. Some bacteria from the order Lactobacillales and order Clostridiales were more enriched in the WHG group than the WNG group during both T1 and T3 (Figs. 2F, 3E). In addition, the abundances of Chloroplast, *Streptophyta*, Peptococcaceae_1, and *Peptococcus* were markedly increased in the WHG group during T3 (Fig. 3F).

The WHG group had higher abundances of *Clostridium_XIVa*, Christensenellaceae, Clostridiales_Incertae_Sedis_XIII, *Christensenella*, *Klebsiella*, *Proteus*, Lactobacillaceae, *Lactobacillus* and *Alloprevotella* than the ENG group during T1 (Fig. 3G). The abundances of *Oscillibacter*, *Parvimonas*, *Peptococcus*, Oxalobacteraceae, and *Oxalobacter* were mainly increased during T3 (Fig. 3H). The WHG group had higher abundances of *Clostridium_sensu_stricto*, Clostridiaceae_1 and *Klebsiella* than the LNG group during T1 (Fig. 3I). Furthermore, there was no difference in the distribution of the GM between the LNG and WHG groups during T3. The overall differences in GM distributions with dynamic changes in FBG across the three trimesters are shown in Fig. 3A–I.

### Analysis of the relationship between GM variance and dynamic changes in blood lipids across the three trimesters

**Fig. 4:**
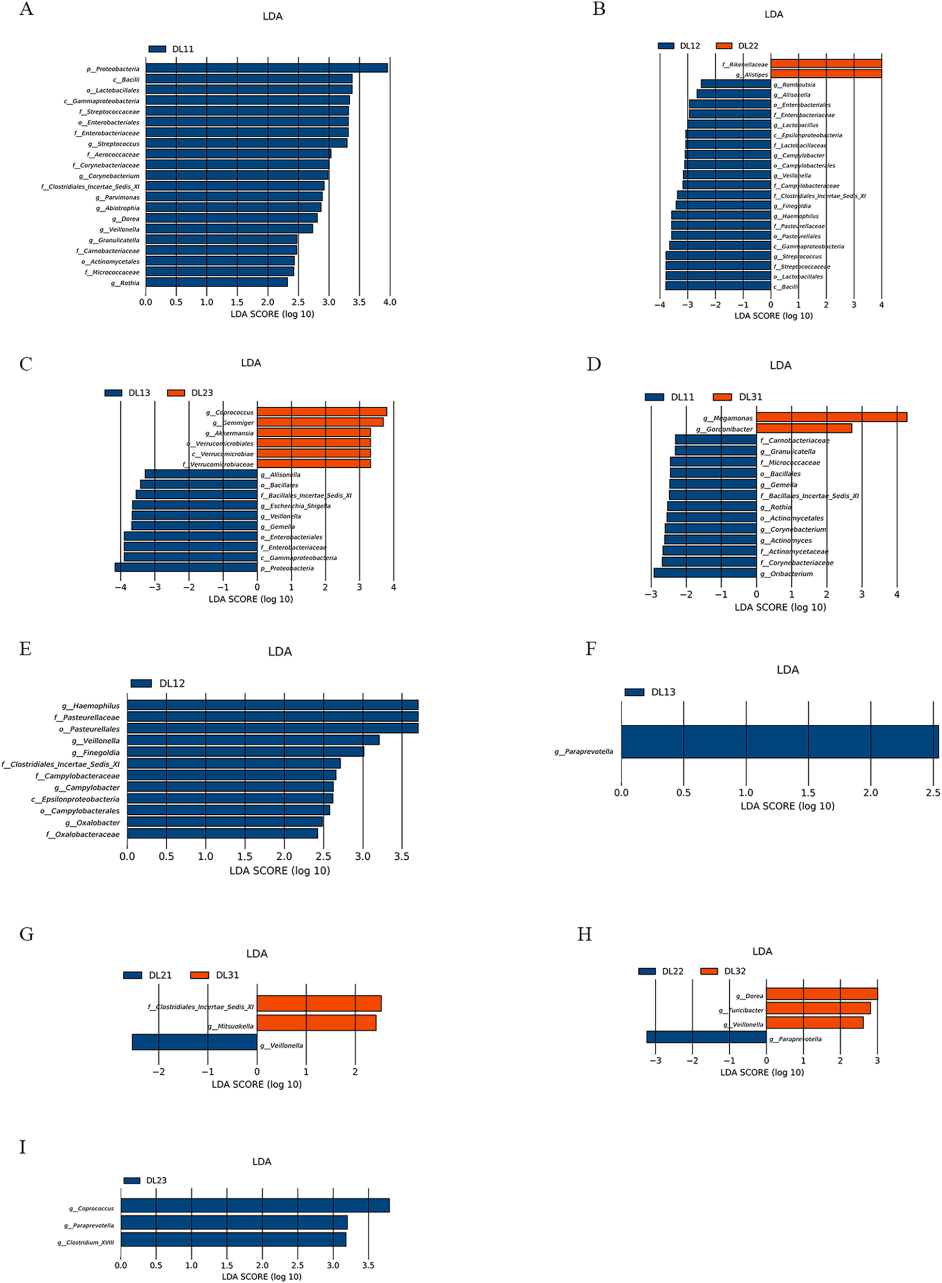
Differentially abundant taxa of gut microbiota with dynamic changes in blood lipids across the three trimesters. There were significant differences by linear discrimination analysis effect size (LEfSe) analysis between DL1 and DL2 groups in the first trimester (T1, **A**), the second trimester (T2, **B**) and the third trimester (T3, **C**), between DL1 and DL3 groups in T1 (**D**), T2 (**E**) and T3 (**F**), between DL2 and DL3 groups in T1 (**G**), T2 (**H**) and T3 (**I**). DL3: with dyslipidemia only in T3, DL2: with dyslipidemia since T2, DL1: dyslipidemia starting from T1 of pregnancy

The relationship between GM distribution and the development of dyslipidemia was further explored (Fig. 4A–I). No differences were shown in the alpha or beta diversities among the DL1, DL2 and DL3 groups in each trimester (Additional files 5 and 6). LEfSe analysis showed that the family Corynebacteriaceae, genus *Corynebacterium,* genus *Rothia* and genus *Granulicatella* were identically more abundant in the GM of the DL1 group than in those of both the DL2 and DL3 groups during T1 (Fig. 4A, D). However, no other changes consistent with the development of dyslipidemia were found among the three groups across the three trimesters (Fig. 4).

### Relationship between the dynamics in the relative abundance of the GM and in blood glucose and lipids across the three trimesters

**Fig. 5:**
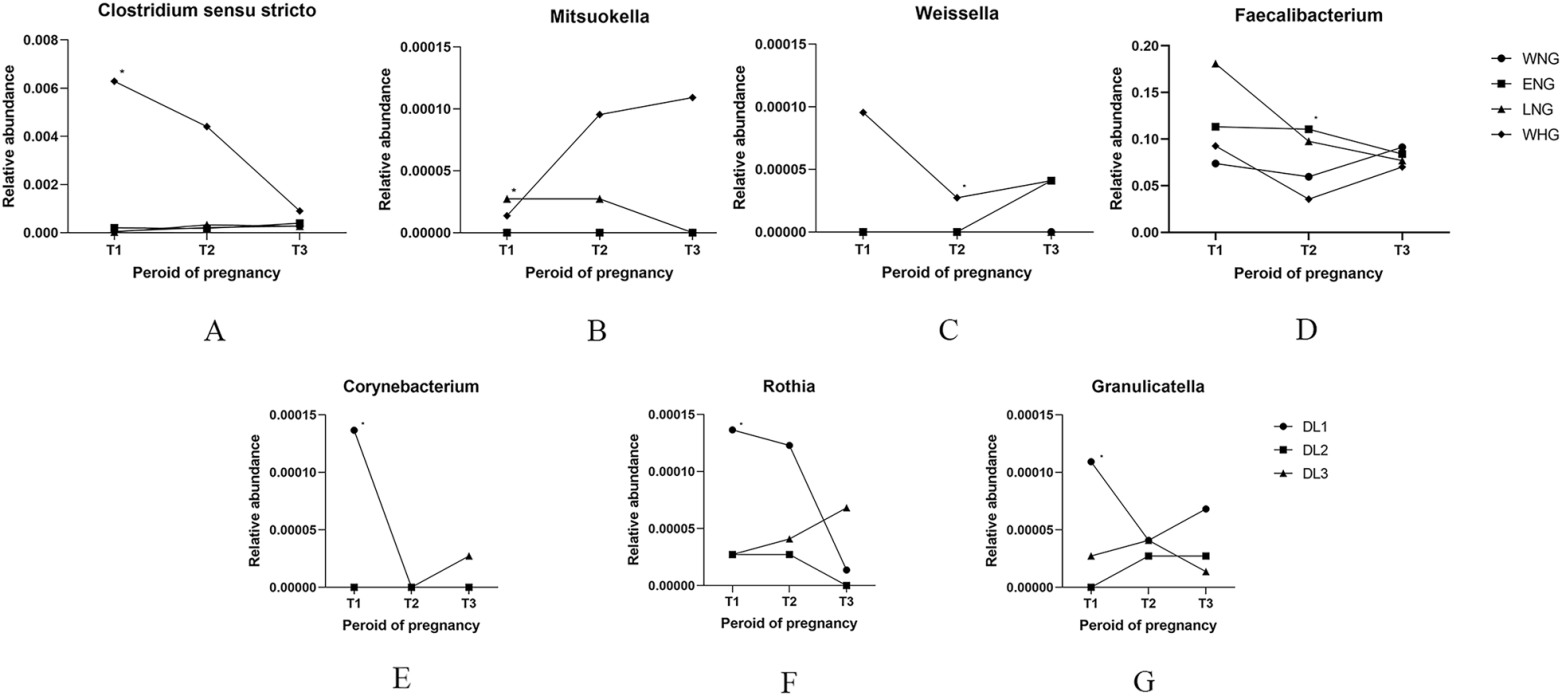
Dynamic changes in the relative abundances of gut bacteria with the dynamic alterations in FBG and serum lipid levels across three trimesters. The different shaped dots represent the median (**A**–**C**, **E**–**G**) or the average relative abundance (**D**) of the gut bacteria in those groups with different onset time of high FBG or dyslipidemia in each trimester. A*: WHG > WNG in T1, *P* = 0.04. B*: LNG > WNG in T1, *P* = 0.038. C*: WHG > ENG in T2, *P* = 0.018. D*: ENG > WHG in T2, *P* = 0.048. E*: DL1 > DL2 and DL3 in T1, *P* = 0.022 and 0.021, respectively. F*: DL1 > DL2 and DL3 in T1, *P* = 0.011 and 0.003, respectively. G*: DL1 > DL2 in T1, *P* = 0.007. T1: the first trimester; T2: the second trimester; T3: the third trimester; WNG: normal FBG during the whole pregnancy, ENG: normal FBG in the early stage (T1) of pregnancy, LNG: normal FBG in the late stage (T3) of pregnancy, WHG: high FBG during the whole pregnancy; DL3: with dyslipidemia only in T3, DL2: with dyslipidemia since T2, DL1: dyslipidemia starting from T1 of pregnancy

Based on the LEfSe analysis of bacteria in T1 above among the groups with different onset time of hyperglycemia and dyslipidemia, the dynamic alterations in their relative abundance at the genus level were further analyzed using one-way ANOVA or Kruskal‒Wallis comparisons during three trimesters. As compared with that of the WNG group, the relative abundances of *Mitsuokella* in the LNG group and *Clostridium *sensu stricto in the WHG group were significantly increased during T1 (Fig. 5A, B). Besides, the relative abundance of *Weissella* was significantly higher while *Faecalibacterium* was markedly lower in the WHG group than the ENG group during T2 (Fig. 5C and D). These phenomena suggest that the four strains mentioned above are potentially involved in the development of high FBG during pregnancy, among which *Mitsuokella*, *Clostridium *sensu stricto* and Weissella* might promote the occurrence of hyperglycemia, while *Faecalibacterium* might have an inhibitory effect.

The relative abundances of *Corynebacterium* and *Rothia* in the DL1 group were significantly higher than those in the DL2 and DL3 groups during T1 (Fig. 5E, F). In addition, the relative abundance of *Granulicatella* was pronouncedly higher in the DL1 group than the DL2 group during T1 (Fig. 5G). There were no significant differences in these three bacteria between the DL2 and DL3 groups during the three trimesters. The findings from the dynamic change analysis suggest that the increases of *Corynebacterium, Rothia,* and *Granulicatella* in the GM during T1 might be related to the occurrence of dyslipidemia during pregnancy.

Finally, the relative abundances of the bacteria mentioned above during the three trimesters had been included into logistic regression analysis for the risk of high FBG or dyslipidemia during pregnancy (data not shown). However, no independent associations were found. It suggests that the alterations in GM might affect the development of hyperglycemia and dyslipidemia through some indirect mechanisms (e.g. microbiota metabolites and inflammatory reactions), which await further exploration.

## Discussion

To our knowledge, this study is the first to reveal dynamic changes in the GM profile during the three trimesters of pregnancy and to analyze the relationships between the GM composition and glycolipid metabolic disorders in each trimester.

In the present study, we found that the abundance of *Bilophila* increased significantly from T1 to T3. The abundances of Erysipelotrichales, Erysipelotrichaceae, Erysipelotrichia, *Clostridium XVIII*, and *Turicibacter* were significantly increased, and the abundance of Alcaligenaceae was markedly lower in T2 than in T1. The abundances of the phylum Actinobacteria and class Actinobacteria were markedly higher, while those of Neisseriales and Firmicutes were lower in T3 than in T1. No dynamic changes in GM abundance were observed between T2 and T3. Like in our study, Koren et al. [7] reported increased abundances of Actinomycetes and Proteobacteria in T3 compared with T1, reflecting a similar microbiome to obese individuals. The abundance of Actinobacteria significantly increased with pregnancy and was found to be related to physiological insulin resistance. However, they also included overweight pregnant women [7]. In addition, the abundance of *Bilophila* was found to be increased in the GM of people on a high-fat diet (HFD) [23], and it synergistically acted with HFD in mice, leading to higher abnormalities in glucose metabolism and hepatic steatosis [24]. These dynamic changes indicate that the GM in T1 may play a predominant role in the regulation of glucose and lipid metabolism throughout pregnancy.

Then based on LEFSe analysis and the Kruskal‒Wallis test of the relative abundances in T1 among the WNG, ENG, LNG and WHG groups, we further selected four kinds of bacteria, *Mitsuokella*, *Clostridium *sensu stricto, *Faecalibacterium* and *Weissella,* for analysis of their relationship with the different onset time of high FBG. The overall comparisons on the dynamic changes in their abundances between WNG, ENG, LNG and WHG groups in each trimester suggest that they are all potentially involved in the elevated FBG level during pregnancy. Liu. et al. found that *Mitsuokella* was dominant in the salivary microbiome of treatment-naive type 2 diabetes (T2D) patients [25]. In addition, the abundance of *Mitsuokella* was significantly increased in obese individuals in another study [26]. However, possibly because of variations in sample size, gestational age, and clinical parameters, there were no consistent conclusions from previous studies on the roles of *Clostridium *sensu stricto, *Faecalibacterium* and *Weissella* in the disturbance of glucose metabolism. *Faecalibacterium* has usually been thought as a producer of butyrate and short-chain fatty acids (SCFAs) with anti-inflammatory actions. It has been found to be negatively associated with FBG and uric acid levels [27, 28] and is significantly decreased in patients newly diagnosed with T2D [29, 30]. However, Liu et al. [31] reported that there was an increased abundance of *Faecalibacterium* in the GM of women with GD, which was positively related to circulatory concentrations of proinflammatory factors. Crusell et al. [10] found an enrichment of species annotated to *Faecalibacterium* in women with GD and varying correlations between different *Faecalibacterium* species in the GM and serum hsCRP levels. Based on our results and the findings above, we inferred that *Faecalibacterium* might play a compensatory and counterbalancing role when hyperglycemia is developed, and the hyperglycemic state might also inhibit its growth, since its relative abundance was markedly higher in the ENG group than the WHG group in T2.

Numerous species from *Weissella* have long been recognized as probiotics, and *Weissella cibaria MW01* can regulate inflammation through the NF-κB-mediated MLCK–pMLC pathway [32, 33]. However, certain species of this genus, including *Weissella confusa* bacteremia, have been found to be opportunistic pathogens [34, 35]. In addition, *Weissella* was found to be enriched in women with GD who failed to respond to medical nutrition treatment (MNT) for glycemic control [36]. *Clostridium *sensu stricto was positively related to waist circumference, BMI and serum lipid levels, including LDL-C, TG and TC levels [37]. However, some studies have shown that it is decreased in T2D patients [38] and women with GD [10]. These results indicate that the different species from *Clostridium *sensu stricto, *Faecalibacterium* and *Weissella* presumably have differential impacts on blood glucose. The role of specific bacteria in abnormal glucose metabolism during the three trimesters of pregnancy and their related mechanisms require further in-depth research.

To date, there has been a lack of attention given to the relationship between dyslipidemia during pregnancy and the GM. The present research explored, for the first time, the dynamic changes in *Corynebacterium*, *Rothia,* and *Granulicatella* from T1 to T3, and these changes may be related to the occurrence of dyslipidemia. *Corynebacterium* and *Rothia* are both from the class *Actinobacteria*, which has been found to be a characteristic microbiota of people with obesity [7]. It has been shown that *Corynebacterium* species require lipids [39]. Moreover, the genome sequencing of 11 species of *Corynebacterium* revealed a wide presence of toxins, multidrug resistance and virulence factors, which are thought to be the source of infection in various diseases [40]. *Rothia* is an opportunistic pathogen that has been found to be associated with various infections [41]. However, Xu et al. demonstrated that as a butyrate-producing bacterium, *Rothia* might play an important role in alleviating atherosclerosis [42]. Studies have reported that *Granulicatella* is associated with numerous infections [43, 44] and was found to be markedly abundant in the salivary microbiome in periodontally healthy obese people [45]. All of these findings indicate that *Corynebacterium*, *Rothia*, and *Granulicatella* are linked to obesity and inflammation, and an increase in their relative abundance may influence lipid metabolism in pregnant women. The metabolism-related results of the abovementioned bacteria in previous studies are summarized in Table 3.

**Table 3:** Metabolism-related findings reported by previous studies about those GM related to high FBG or dyslipidemia found in this study

Microbiota	Previous studies			The present study	
	Author	Year	Main findings	FBG	Blood lipids
Mitsuokella	Liu et al. [25]	2021	*Mitsuokella* was significantly dominant in the salivary microbiome for treatment-naive T2D patients	↑^a^	
	Palmas et al. [26]	2021	*Mitsuokella* from Firmicutes taxa was significantly increased in obese subjects, while Firmicutes taxa positively correlated with body fat and negatively with muscle mass		
Faecalibacterium	Yuan et al. [27]	2022	The serum uric acid level negatively associated with *Faecalibacterium*, which is one of the producers of short-chain fatty acids (SCFAs)	↓^b^	
	Ye et al. [28]	2019	The relative abundance of *Faecalibacterium* was lower in pregnant women with GD whose blood glucose failed to get control after lifestyle modifications		
			The relative abundance of *Faecalibacterium* was negatively correlated with FBG		
			The peroxisome proliferator-activated receptor was positively correlated with the relative abundance of *Faecalibacterium*		
	Wu et al. [29]	2020	The abundance of *Faecalibacterium sp* were decreased in treatment-naive T2D patients		
	Ferrocino et al. [46]	2018	*Faecalibacterium* was inversely correlated to FBG level in patients with GD		
	Crusell et al. [10]	2018	Enrichment of species annotated to *Faecalibacterium* were showed in GD cohort compared with the normoglycaemic pregnant women		
	Liu et al. [31]	2020	The gut microbiota of women with GD showed increased abundance of *Faecalibacterium,* and the relative abundance of *Faecalibacterium* was positively related to inflammatory factor concentrations		
Weissella	Huang et al. [32]	2020	*Weissella cibaria MW01* could regulate inflammation through the NF-κB-mediated MLCK–pMLC pathway	↑^a^	
	Chen et al. [36]	2022	*Weissella* was found to be enriched in GD women who failed to respond to medical nutrition treatment for glycemic control		
Clostridium sensu stricto	Martínez-Cuesta et al. [47]	2021	The relative abundance of *Clostridium *sensu stricto was significantly diminished in obese individuals	↑^a^	
	Zeng et al. [37]	2019	*Clostridium *sensu stricto was positively related to waistline, BMI and serum lipid levels including LDL-C, TG and TC		
	Maskarinec et al. [38]	2021	*Clostridium *sensu stricto was more abundant in T2D patients		
	Crusell et al. [10]	2018	There was a depletion of species annotated to *Clostridium *sensu stricto in GD patients		
Corynebacterium	Nasim et al. [40]	2021	11 species of *Corynebacterium* revealed a wide presence of toxins, multi-drug resistance and virulence factors, which are thought to be the source of infection in various diseases		↑^a^
			Diphtheroid is the last common ancestor of all the *Corynebacterium* species		
	Li et al. [50]	2021	Serum/liver lipid and carbohydrate profiles were found strongly negatively correlated with *Corynebacterium*		
Rothia	Fatahi-Bafghi et al. [41]	2021	The genus *Rothia* are emerging as opportunistic pathogens associated with various infections, including endocarditis, pneumonia, peritonitis and, septicemia, abdominal infection, tonsillitis, spondylodiscitis, keratitis, meningitis, catheter-related infection, prosthetic device infection, osteomyelitis, peritoneal fluid, sputum, synovial fluid, bile, and bronchitis		↑^a^
	Xu [42]	2022	*Rothia* might play an important role in alleviating atherosclerosis		
Granulicatella	Li et al. [43]	2022	*Granulicatella* was the independent risk factor for death of Hepatic encephalopathy after transjugular intrahepatic portosystemic shunt		↑^a^
	Aranaz et al. [44]	2021	*Granulicatella* were more abundant in subjects with high inflammatory score		
	Wu et al. [45]	2018	The abundance of *Granulicatella* in salivary microbiome was significantly increased in periodontally healthy obese people		
	Crusell et al. [10]	2018	*Granulicatella* was a biomarker of GD		
	Dong et al. [48]	2021	The abundance of *Granulicatella* in salivary microbiome was significantly higher in subjects with high serum TSH level (> 4.2 mIU/L) compared with subjects with normal TSH level (0.6–4.20 mIU/L)		
	Zhao et al. [49]	2020	As a risk factor for the development of metabolic-associated fatty liver disease, insulin resistance was positively correlated with the abundance of *Granulicatella* in supragingival plaques		

^a^ "↑" represents the corresponding microbiota may promote the occurrence of high FBG or Blood lipids levels in the present study

^b^ "↓" represents the corresponding microbiota may have an inhibitory effect of the development of high FBG in the present study

At phylum level, Bacteroidetes and Firmicutes are the dominant microbiota in the intestine, and growing attention is being paid to the question of whether there is a connection between the abundance ratios of the two and metabolic diseases. In this study, the abundance changes of the two dominant bacteria and the dynamic B/F ratio were first investigated across the whole pregnancy. The disproportionate of the abundance ratio of Bacteroidetes to Firmicutes not only affects carbohydrate metabolism but also changes the production of SCFAs and induces insulin resistance [51–53]. Bacteroidetes are mainly involved in bile acid and steroid metabolism, while Firmicutes mainly participate in polysaccharide metabolism and produce butyrate. Firmicutes start to take the lead as gestational age increases, which is comparable to the features of the GM seen in obese people [10, 54]. Contrary to previous beliefs, our results indicate that the dominant microbiota significantly shifted from Firmicutes to Bacteroidetes during the three trimesters in women who were F-dominant in T1. Studies have found a higher Firmicutes/Bacteroides ratio among both patients with prediabetes and patients newly diagnosed with T2D [30]. Among pregnant women, Cortez et al. found that women with GD had a higher Firmicutes/Bacteroides ratio [12]. Likewise, we noticed that FBG levels were higher in the F-dominant group than in the B-dominant group in all periods. However, neither their study nor ours showed any statistically significant differences between metabolic changes during pregnancy and shifts in the B/F ratio. These results indicate that changes in the dominant microbiota may not directly affect glucose or lipid metabolism. However, the proportion of minor microbiota, although low in abundance, may have a higher predictive value for metabolic disorders.

The strengths of this study are that it is currently the first study to analyze the dynamic changes in the microbiome during pregnancy, compare the differences in the GM between participants with different glycolipid metabolism statuses during pregnancy and identify bacteria related to the occurrence of high FBG levels and dyslipidemia. However, our study has several limitations. First, the number of cases in this study was too small to allow for further analysis of outcomes, and a larger sample size and a multicenter cohort study are necessary to confirm any pertinent findings. In addition, only FBG levels in the third trimester were analyzed in this study, and no oral glucose tolerance test was performed for the diagnosis of GD. In addition, one pre-gestational overweight woman was included in the study, which may influence the GM composition during pregnancy. We also plan to use blood metabolomics to explore the link between the changes in the GM and glycolipid metabolism. Finally, we admitted that T2 is longer than T1 and T3, and only once sampling might not well reflect the conditions during the whole T2. We would collect the samples at 4–6 week intervals during T2 in the future study.

## Conclusion

There are dynamic changes in the GM during the three trimesters, and the alterations in some bacterium abundance may contribute to the development of high FBG and dyslipidemia during pregnancy. Analysis of the GM during T1 may help to predict abnormal glucose and lipid metabolism during pregnancy and be targeted for intervention. In addition, some low-abundance microbiota may have a higher predictive value for the occurrence of aberrant glycolipid metabolism than those main bacteria. Attention should be given to the GM in T1 for early intervention, but the exact relationship between changes in the GM and glycolipid metabolism disorders still needs to be further explored.

### Supplementary Information

**Additional file 1.** Part of the questionnaire given to the pregnant women at the time of enrollment in early pregnancy.

**Additional file 2.** Comparisons of the Chao1 index of alpha diversity and PCoA of the beta diversity of the gut microbiota during the three trimesters. P1–3: The horizontal axis: the grouping of samples, the Longitudinal axis: Chao1 index. *P* values are shown on the middle line and in the upper left corner of each chart. P4–6: The horizontal and longitudinal axis represent the first and second principal coordinates, respectively; the percentage indicates the contribution rate of the corresponding principal coordinate to the sample difference; the *P* value is the test *P* value of the corresponding principal coordinate; the dots indicate each sample; and different colors indicate that the samples belong to different groups. The horizontal box chart shows the distribution of values of different groups on the first principal coordinate; the vertical box chart shows the distribution of values of different groups on the second principal coordinate. There were neither significant differences in the alpha diversity of the gut microbiota (GM) between T1 and T2 (P1), T1 and T3 (P2), T2 and T3 (P3), nor did the beta diversity of the GM analysis between T1 and T2 (P4), T1 and T3 (P5), T2 and T3 (P6). T1: the first trimester; T2: the second trimester; T3: the third trimester

**Additional file 3.** Comparisons of the Chao1 index of alpha diversity of the gut microbiota in those groups with different onset time of high FBG across the three trimesters. There were no significant differences in the alpha diversity of the gut microbiota (GM) between WNG and ENG (P1–3), WNG and LNG (P4–6), WNG and WHG (P7–9), WHG and ENG (P10–12) and WHG and LNG (P13–15) in T1, T2 and T3. *P* value is shown in each chart. T1: the first trimester; T2: the second trimester; T3: the third trimester; WNG: normal FBG during the whole pregnancy, ENG: normal FBG in the early stage (T1) of pregnancy, LNG: normal FBG in the late stage (T3) of pregnancy, WHG: high FBG during the whole pregnancy.

**Additional file 4:** PCoA of the beta diversity of the gut microbiota in those groups with different onset time of high FBG across the three trimesters. There were no significant differences in the beta diversity of the gut microbiota (GM) between WNG and ENG (P1–3), WNG and LNG (P4–6), WNG and WHG (P7–9), WHG and ENG (P10–12) and WHG and LNG (P13–15) in T1, T2 and T3. *P* value is shown in each chart. All the abbreviations as described in Additional file 3.

**Additional file 5.** Comparisons of the Chao1 index alpha diversity of the gut microbiota in those groups with different onset time of dyslipidemia across the three trimesters. There were no significant differences in the alpha diversity of the gut microbiota (GM) between DL1 and DL2 (P1–3), DL1 and DL3(P4–6), DL2 and DL3 (P7–9) in T1, T2 and T3. *P* value is shown in each chart. T1: the first trimester; T2: the second trimester; T3: the third trimester; DL3: with dyslipidemia only in T3, DL2: with dyslipidemia since T2, DL1: dyslipidemia starting from T1 of pregnancy.

**Additional file 6.** PCoA of the beta diversity of the gut microbiota in those groups with different onset time of dyslipidemia across the three trimesters. There were no significant differences in the beta diversity of the gut microbiota (GM) between DL1 and DL2 (P1–3), DL1 and DL3(P4–6), DL2 and DL3 (P7–9) in T1, T2 and T3. *P* value is shown in each chart. All the abbreviations as described in Additional file 5.

## Data Availability

The data used during the current study are available from the corresponding author upon reasonable request.

## Abbreviations

GM: Gut microbiota
FBG: Fasting blood glucose
T1: The first trimester
T2: The second trimester
T3: The third trimester
GD: Gestational diabetes
TG: Triglyceride
TC: Total cholesterol
HDL-C: High-density lipoprotein cholesterol
LDL-C: Low-density lipoprotein cholesterol
FT4: Free thyroxine
TSH: Thyroid stimulating hormone
TPOAb: Thyroid peroxidase antibody
TgAb: Thyroglobulin antibodies
ADA: American Diabetes Association
OTUs: Operational taxonomic units
SRA: Sequence Read Archive
PCoA: Principal coordinates analysis
LEfSe: Linear discrimination analysis effect size
LDA: Linear discrimination analysis
WNG: Pregnant women with normal FBG level during the whole course of pregnancy
ENG: Pregnant women with normal FBG level in T1 while with high FBG level in T2 and/or T3
LNG: Pregnant women with normal FBG level in T3 while with high FBG level in T1 and/or T2
WHG: Pregnant women with high FBG level during the whole course of pregnancy
IADPSG: International Association of the Diabetes and Pregnancy Study Groups
DL1: Pregnant women with dyslipidemia starting from T1 of pregnancy
DL2: Pregnant women with dyslipidemia since T2
DL3: Pregnant women with dyslipidemia only in T3
B/F: The abundance ratio of Bacteroidetes/Firmicutes
HFD: High-fat diet
T2D: Type 2 diabetes
SCFAs: Short-chain fatty acids
