# Supplementary material for: Dynamic changes in the gut microbiota during three consecutive trimesters of pregnancy and their correlation with abnormal glucose and lipid metabolism
Source: Eur J Med Res. 2024 Feb 12;29:117. doi: 10.1186/s40001-024-01702-0 (PMC10860297; doi:10.1186/s40001-024-01702-0)
Supplement: Supplementary file 1 — Additional file 1. Part of the questionnaire given to the pregnant women at the time of enrollment in early pregnancy. [file 40001_2024_1702_MOESM1_ESM.pdf]

Survey Location: ___________

Posting of questionnaire code bar

Health Survey of Natural Population in Northeast Region of China

(Mother in Early Pregnancy)

Informed Consent

This health survey was funded by the Key Research and Development Program of the Ministry of Science and Technology of China.

Purpose and content: To understand the health status of the natural population in the Northeast region of China. Questionnaires and physical examinations will be conducted at baseline and follow-up visits. The physical examination will mainly include height, weight, abdominal circumference, blood pressure, body composition, biochemical tests and urinary and stool routine. In the meantime, we will establish a population biospecimen bank for future health-related genetic and histological studies at the population level. A portion of the biological sample remaining from your test will need to be retained at the time of the physical examination.

Possible Risks: The possible risks associated with this investigation are no greater than those associated with routine medical checkups.

Benefits and Advantages: You will receive a free medical report and expert advice that reflects your physical and health status.

Confidentiality commitment: We guarantee that under no circumstances will your personal information and examination results in any form of disclosure.

Voluntary principle: This survey medical examination is voluntary, you can refuse to participate or withdraw at any time.

I have communicated in detail with the subject staff and fully understand the purpose, content, possible risks and benefits of this survey, and hereby voluntarily participate in this survey.

Signature: _______________ **or** ___________ **(handprint)**

Part I Basic Information

A1. **Name:** ________

A2. **Ethnicity:** _________

A3. **ID number** |__|__|__|__|__|__ |__ |__ |__ |__ |__|__ |__|__|__|__|__|__|__|

A4. **Does your actual date of birth match your ID card?**

1…Yes 2...No, **Actual date of birth** _______year____month___ day

A5. **Maternal Health Handbook:** ____

A6. **Is your current residential address the same as your registered address?**

1 …Yes (**skip to** A7)

2 …No, **Your current address:** _____ Province / Autonomous Region_____City / League_____District / County_____Town / Township_____ Village

A7. **Your contact information:** 1. **Mobile phone number** (required)___________

2. **□Landline phone/□WeChat/□Email/□Other cell phone number**_______________

A8. **Your husband's contact information:** 1. **Mobile phone number** (required)______________

2. **□Fixed-line phone/□WeChat/□Email/□Other cell phone number**_______________

A9. **What is your current main occupation?**

1. farmer 2. worker 3. organ worker (civil servant) 4. professional and technical staff 5. service, salesman (including driver) 6. retired 7. housework 8. freelance 9. student 10. unemployed

11. other________

A10. **Name of your current workplace?**

____

A11. **Your annual household income: ______10,000 yuan/year**;
Number of persons in your home: _____people.

A12. **Your education level is：**
1. uneducated 2. elementary school 3. junior high school 4. high school or junior college 5. college
6. university 7. graduate and above

A13. **Your current marital status：**
1. unmarried 2. married/cohabiting 3. separated/divorced 4. widowed

A14. **Participation in health insurance (multiple choice)：**
2. urban employees' medical insurance 2. urban residents' medical insurance 3. Agricultural Cooperative insurance 4. Commercial Insurance 5. publicly funded health care 6. medical assistance 7. no medical insurance 8. Other___________

Part II Reproductive History

B1. How old were you when you had your first menstrual period： _____Age

B2. Last menstrual period： _______year____month___day

B2A1. week of gestation： ____week

B2A2. expected date of delivery： _______year____ month____day

B2A3. pre-pregnancy weight： _____kg

B2A4. pre-pregnancy waist circumference： _____cm or____ ft

B2A5. height： _____cm

B3. Have you ever been pregnant？
1. Yes **B3A1. live birth____ times**
2. No (**skip to B8**)

B4. number of abnormal pregnancies： __times

B4A1. abortion： __times

B4A1a. Spontaneous abortion： __times **B4A1b. Medication abortion： __times**

B4A1c. Induced abortions： __times

B4A1d. Induced abortions(embryonic arrest, before 20 weeks of gestation)： __times

B4A2. Fetal death in utero after 20 weeks of gestation： __ times

B4A3. Fetal death during delivery： __ times **B4A4. Neonatal death： __times**

B4A5. Children born with birth defects： __times Defect type： _____

B4A6. Premature birth： __times **B4A7. low birth weight babies： ____times**

B4A8. huge babies： __times

B4A9. less than gestational age： ___times **B4A10. greater than gestational age： __times**

B5A1. Your age at the time of your first child birth (i.e. live birth)： ______years old

B5A2. Your age at the time of your last child birth (i.e. live birth)： _____years old

B6. Have you ever breastfed your child？
1. Yes
2. No **(skip to B8)**

B7. What is your age (age at delivery) and duration of breastfeeding for each live birth？

B7A1. Live births	B7A2. Fertility age	B7A3. Length of breastfeeding
First child	____years old	____month
Second child	____years old	____month
Third child	____years old	____month

B8. Your level of pregnancy vomiting：
1. no vomiting 2. mild vomiting 3. frequent vomiting 4. severe vomiting of pregnancy

B9. Have you ever undergone assisted reproductive technology？

1. Yes，**B9A1. what kind of assisted reproductive technology:** 1. artificial insemination 2. in vitro fertilization
2. No

B10. Have you ever had an IUD in place?
1. Yes， **B10A1. Age of IUD insertion: ___ years old； B10A2. Age of IUD removal: ___ years old**
2. No

B11. Have you ever used oral contraceptives？
1. Yes，**B11A1.You** were ____years old at the time of the first dose；
B11A2.You were ___ years old **at the time of the last dose；**
B11A3. It took a total of___month(s)
2. No

Part III Disease and Surgical History

C1. Frequency of your weekly bowel movements：
1. >1 time/day 2. 1 time/day 3. 1 time/2-3 days 4. <3 times/week

C2. How often your gums bleed when you brush your teeth：
1. rarely or not at all 2. occasionally 3. often 4. rarely or never brush

C3. Have you ever been diagnosed by a doctor with any of the following conditions (prior to this pregnancy) ?

Name of disease	Whether suffering from the disease	Age of first diagnosis
C4.Type I diabetes	1. yes 2. No	C4A1_______years old
C5. Type II diabetes	1. yes 2. No	C5A1_______years old
C6. Hypertension	1. yes 2. No	C6A1_______years old
C7. Cardiovascular disease	1. yes 2. No	C7A1_______years old
C8. Polycystic ovary syndrome	1. yes 2. No	C8A1_______years old
C9. Depression	1. yes 2. No	C9A1_______years old
C10. Anxiety	1. yes 2. No	C10A1_______years old
C11. Other disease (chronic disease) C11A1. __________	1. yes 2. No	C11A2 _______years old

Have you ever been diagnosed by a doctor with any of the following pregnancy-related conditions? (First time pregnancy is not required, skip to C18)

Name of disease	Whether suffering from the disease	Age of first diagnosis
C12. Gestational diabetes	1. yes 2. No	C12A1_______years old
C13. Hypertension in pregnancy	1. yes 2. No	C13A1_______years old
C14. Pre-eclampsia	1. yes 2. No	C14A1_______years old
Depression<br>C15A1. Antenatal depression<br>C15B1. Postpartum depression	1. yes 2. No	C15A2_______years old<br>C15B2_______years old
Anxiety<br>C16A1. Antenatal anxiety<br>C16B1. Postpartum anxiety	1. yes 2. No	C16A2 ______years old<br>C16B2 ______years old
C17. Other diseases	1. yes 2. No	C17A2 ______years old

C17A1.______________		

C18. Have you ever had surgery？
1. Yes 2. No(**skip to next section**)

Name of surgery	Age of surgery	Whether it has been excised	Excision site
C19A1. ______________	C19A2.______years old	C19A3. ______years old	C19A4. ___________
C20A1.______________	C20A2.______years old	C20A3. ______years old	C20A4. ___________
C21A1.______________	C21A2.______years old	C21A3.______years old	C21A4. ___________
C22A1.______________	C22A2.______years old	C22A3.______years old	C22A4. ___________

Part V. History of health care product consumption

Have you taken any of the following medications or supplements regularly **in the past one year and since you were pregnant?**

Health products (※ At least 3 times a week)	Whether have taken	How many months taken before pregnancy?	How many months taken during early pregnancy?
**E1. vitamin A**	1. Yes 2. No	E1A1_______	E1A2_______
**E2. vitamin B**	1. Yes 2. No	E2A1_______	E2A2_______
**E3. vitamin C**	1. Yes 2. No	E3A1_______	E3A2_______
**E4. vitamin D**	1. Yes 2. No	E4A1_______	E4A2_______
**E5. vitamin E**	1. Yes 2. No	E5A1_______	E5A2_______
**E6. vitamin complex**	1. Yes 2. No	E6A1_______	E6A2_______
**E7. Calcium**	1. Yes 2. No	E7A1_______	E7A2_______
**E8. Iron**	1. Yes 2. No	E8A1_______	E8A2_______
**E9. Zinc**	1. Yes 2. No	E9A1_______	E9A2_______
**E10. Iodine preparations**	1. Yes 2. No	E10A1_______	E10A2_______
**E11. Cod liver oil/ DHA**	1. Yes 2. No	E11A1_______	E11A2_______
**E12. Folic acid**	1. Yes 2. No	E12A1_______	E12A2_______
**E13. Elevit**	1. Yes 2. No	E13A1_________	E13A2_______
**E14. Other：** _________	1. Yes 2. No	E14A1_______	E14A2_______

Part VI Personal habits

F1. Did you smoke **before you became pregnant** **(at least 1 cigarette per day** for **more than 6 months)**
1. yes 2. No(**skip to F4**)

F2 At what age did you start smoking daily? ________years old

F3 In general, how many cigarettes do you smoke per day? ______Sticks/day

F4. Did you smoke **during your pregnancy?** (At least 1 cigarette per day)
1. yes
2. No (**skip to F7 if** never smoked**; skip to F5** if less than 1 cigarette per day**;** answer **F4A1** and **F4A2** if you have quit smoking)

F4A1 The age at which you quit smoking: _____ years old

F4A2.What is the most important reason that motivated you to quit smoking? (Skip to F7)

1. preparing for pregnancy 2. suffering from illness 3. financial reasons
4. worried about future health 5. family members' opposition 6. other

F5. Have you smoked today?

1. Yes **F5A1. total______branches，F5A2. ______branches one hour before the blood draw**

2. No

F6. Has your smoking status changed significantly <u>during your pregnancy</u> compared to before?

1. 1. little change 2. significantly more than before 3. significantly less than before

F7. Did you drink alcohol <u>before</u> your <u>pregnancy</u> (at least 1 time per week for **more than 6 months)**

1. Yes 2. No(**skip to F11**)

F8. At what age did you start drinking alcohol regularly?_______years old

F9. Do you blush after drinking?

1. Yes, after the first sip of wine
2. Yes, after a small amount of wine
3. Yes, only after a large amount of wine
4. None

F10. Do you get dizzy after drinking?

1. Yes, after the first sip of wine
2. Yes, after a small amount of wine
3. Yes, only after a large amount of wine
4. None

F11 Do you still drink alcohol <u>during pregnancy?</u>

1. Yes
2. No (**skip to F13** if never drink; **skip to F12** if less than once a week; answer **F11A1 and F11A2** if quit drinking)

F11A1. The age at which you quit drinking is_____years old

F11A2. What was the most important reason that motivated you to quit drinking? (**Skip to F13**)

1. preparing for pregnancy 2. suffering from illness 3. financial reasons
4. fear of future health 5. family members' opposition 6. other

F12. Has your alcohol consumption <u>during pregnancy</u> changed significantly from what it was before?

1. little change 2. significantly more than before 3. significantly less than before

F13. Did you drink tea <u>before you got pregnant</u> (at least 1 time per week for more than 6 months)

1. Yes 2. No(**skip to F16**)

F14. At what age did you start drinking tea regularly?_______years old

F15. How many times a day do you change your tea leaves?______times

F16. Do you still drink tea <u>during your pregnancy</u>?

1. Yes
2. No (If you never drink tea, **skip to F18;** if you drink tea less than once a week, **skip to F17;** if you quit tea, answer **F16A1**)

F16A1. The age at which you stopped drinking tea is years old (skip to F18)

F17. Has your tea drinking changed significantly <u>during your pregnancy</u> compared to a few years ago?

1. Not much change 2. Significantly more than before 3. Significantly less than before

Carbonated beverage consumption

F18. Did you drink carbonated beverages __before your pregnancy__? (at least 1 time per week for more than 6 months)

1. Yes   2. No (**Skip to F20**)

F19. At what age did you start drinking carbonated beverages regularly? _______years old

F20. do you still drink carbonated drinks __during your pregnancy?__

1. Yes

2. No (**skip to F22 if you** never drink carbonated drinks**; skip to F21 if you drink** carbonated drinks less than once a week**;** answer **F20A1** if you quit drinking carbonated drinks)

F20A1. The **age at which you stopped drinking carbonated beverages is_______years old** (**Skip toF22**)

F21. Has your consumption of carbonated beverages __during pregnancy__ changed significantly compared to a few years ago?

1. Not much change   2. Significantly more than before   3. Significantly less than before

Coffee consumption

F22. Did you drink coffee __before you became pregnant__? (at least 1 time per week for **more than 6 months)**

1. Yes   2. No (**Skip to F24**)

F23. At what age did you start drinking coffee regularly? _______years old

F24. Do you still drink coffee __during your pregnancy__?

1. Yes

2. No (If you never drink coffee, **skip to the next section; if you drink coffee** less than once a week, **skip to F25;** if you quit coffee, answer **F24A1**)

F25. Has your coffee drinking changed significantly __during your pregnancy__ compared to a few years ago?

1. Not much change   2. Significantly more than before   3. Significantly less than before
