# Supplementary figures and images for: Dynamic changes in the gut microbiota during three consecutive trimesters of pregnancy and their correlation with abnormal glucose and lipid metabolism
Source: Eur J Med Res. 2024 Feb 12;29:117. doi: 10.1186/s40001-024-01702-0 (PMC10860297; doi:10.1186/s40001-024-01702-0)

Alpha diff boxplot

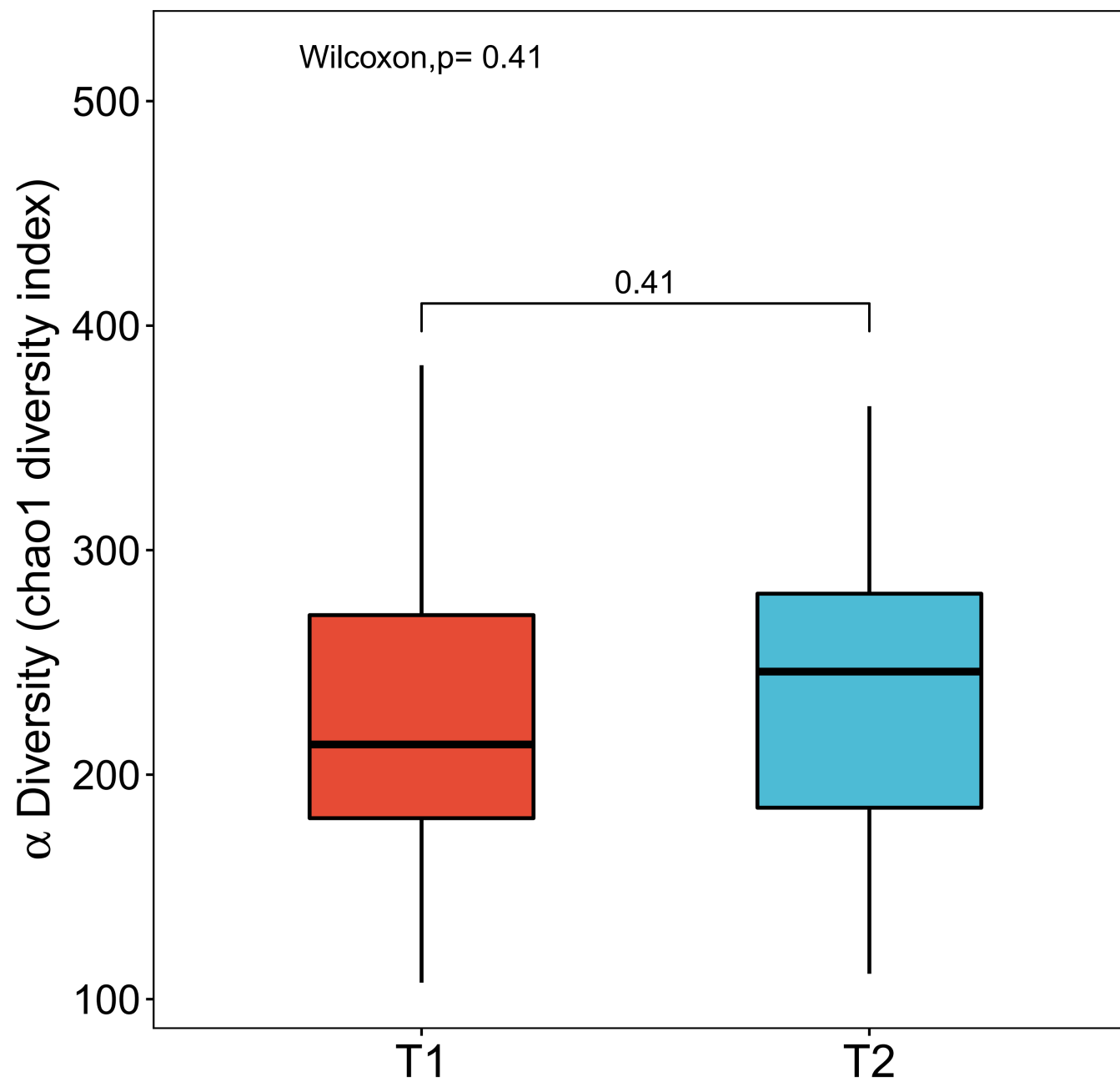

Alpha diff boxplot

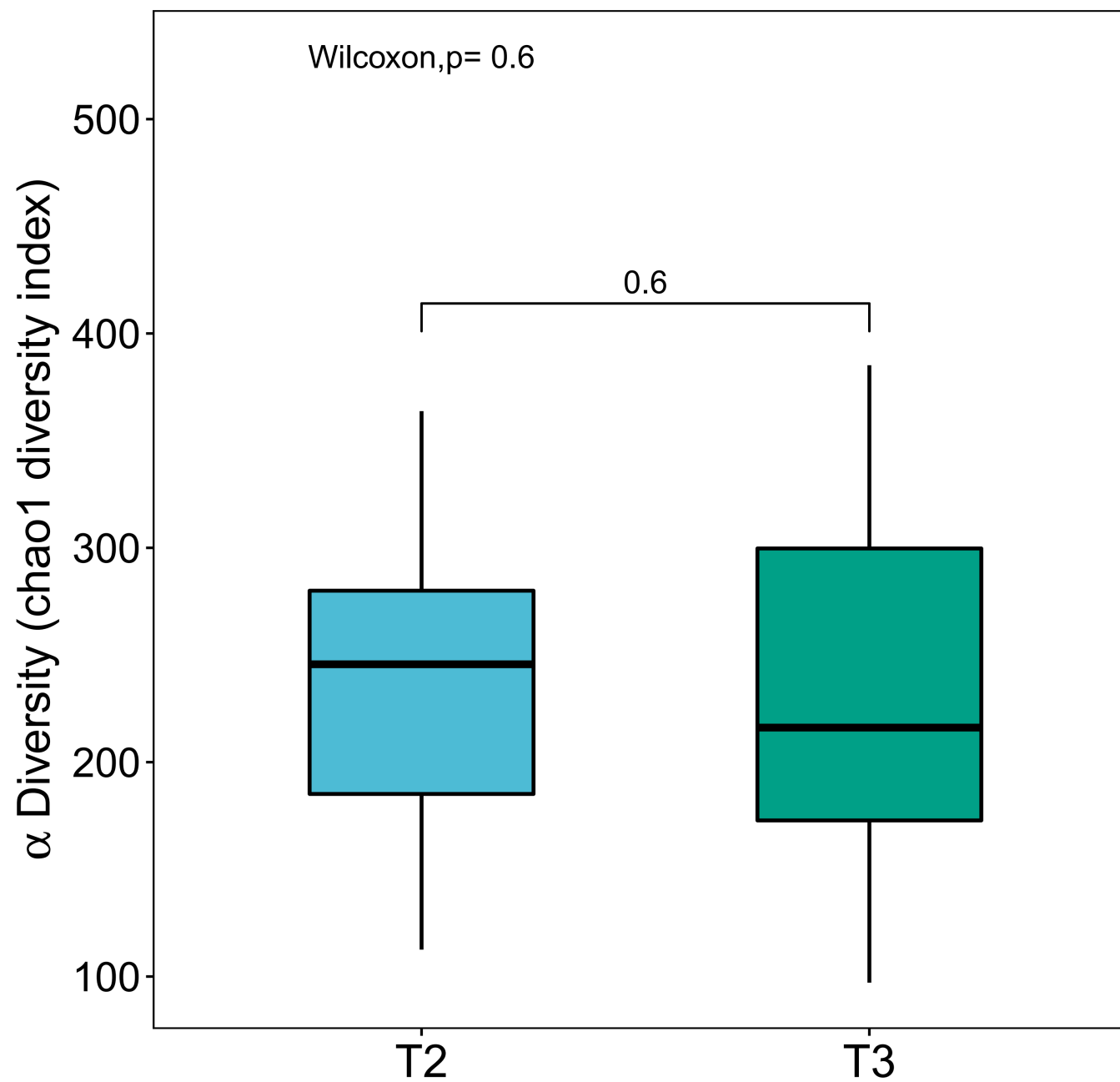

Alpha diff boxplot

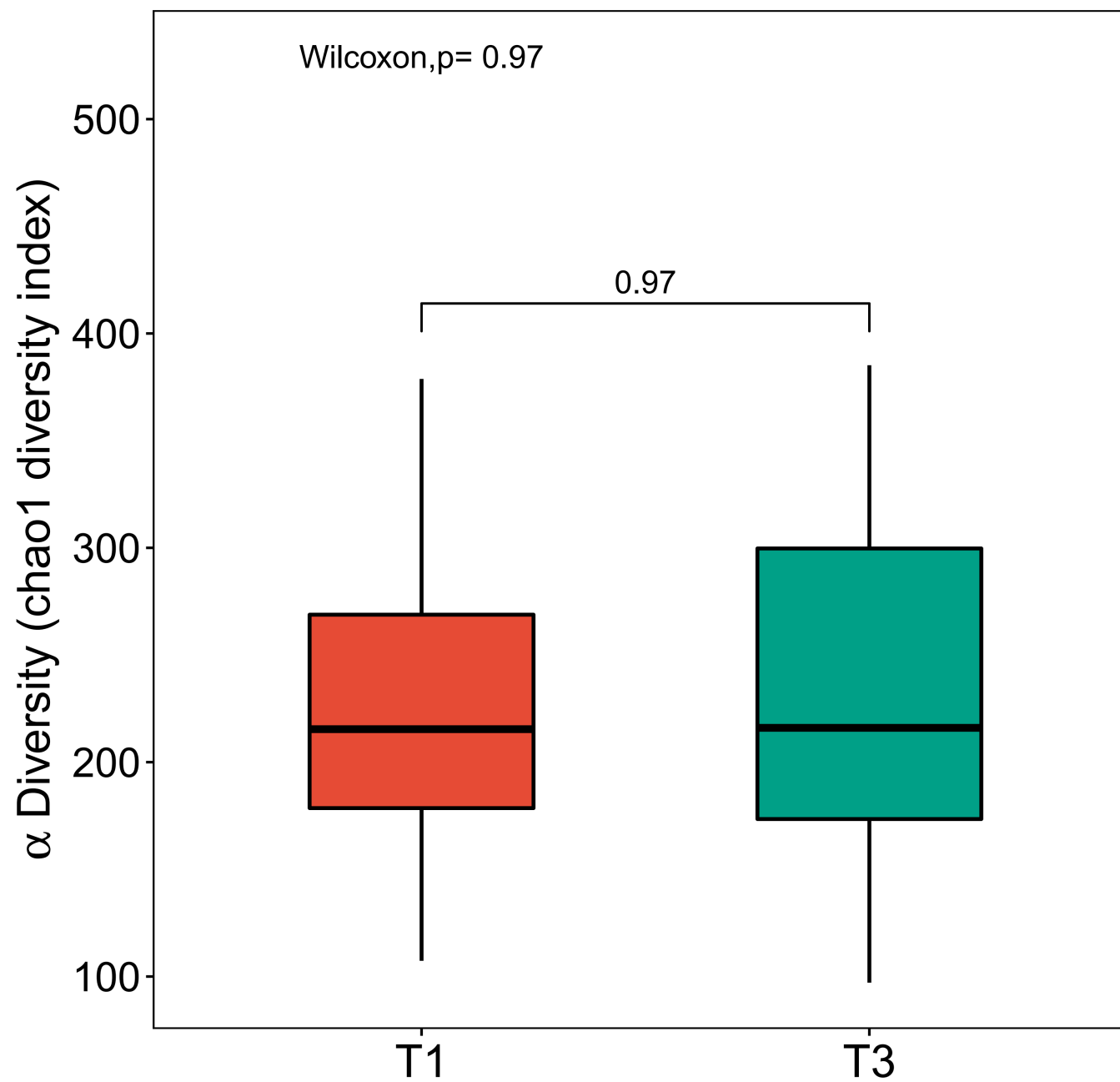

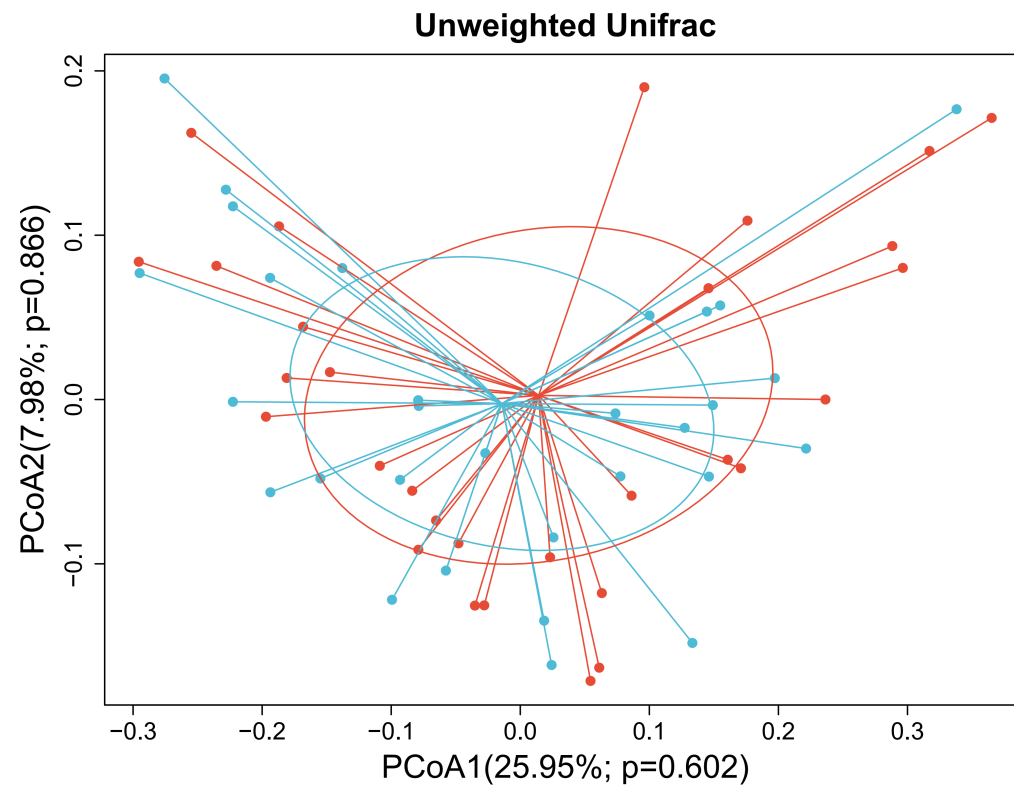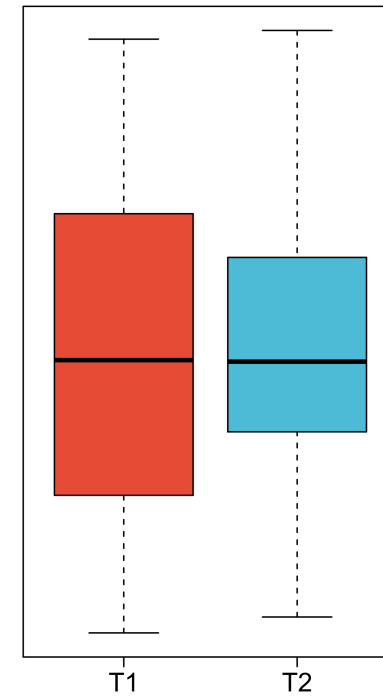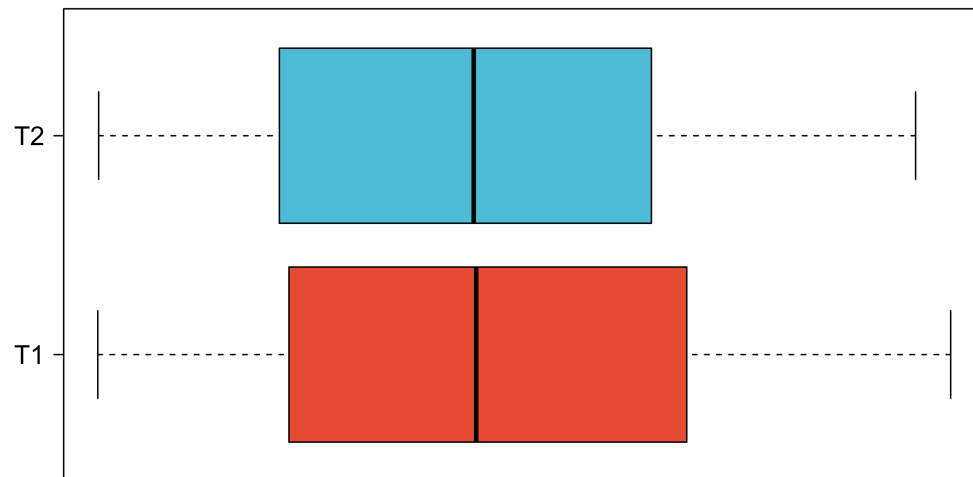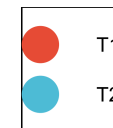

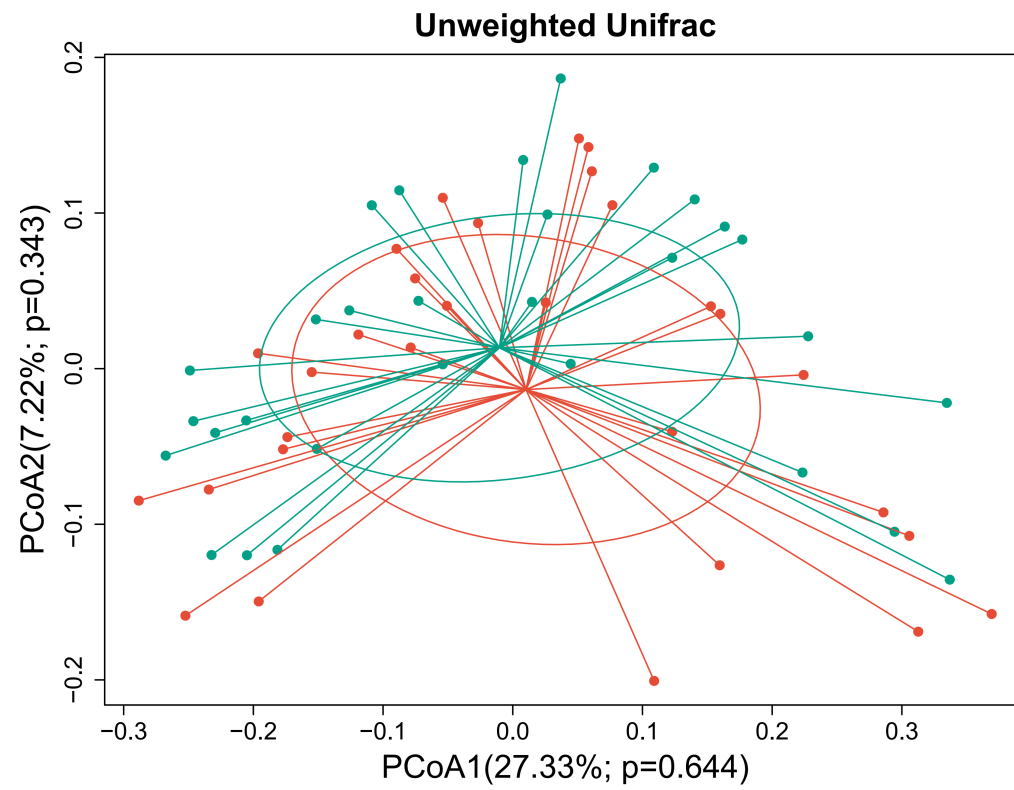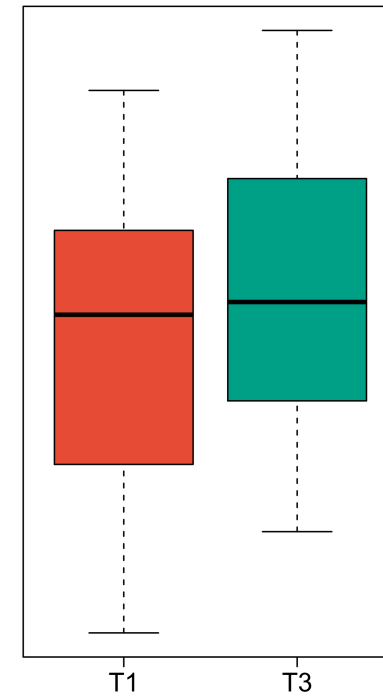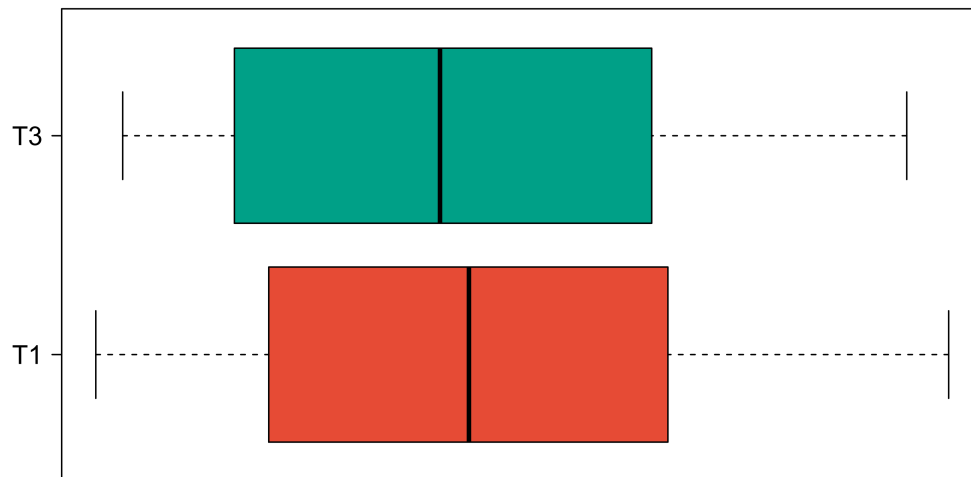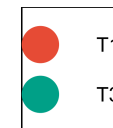

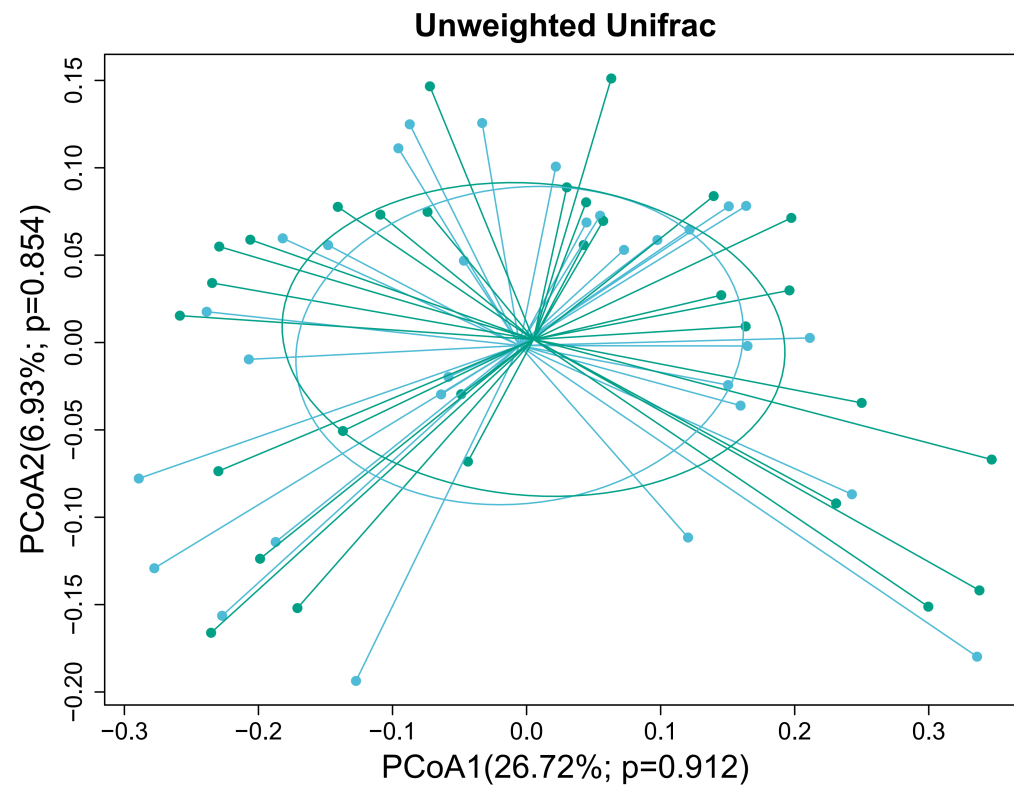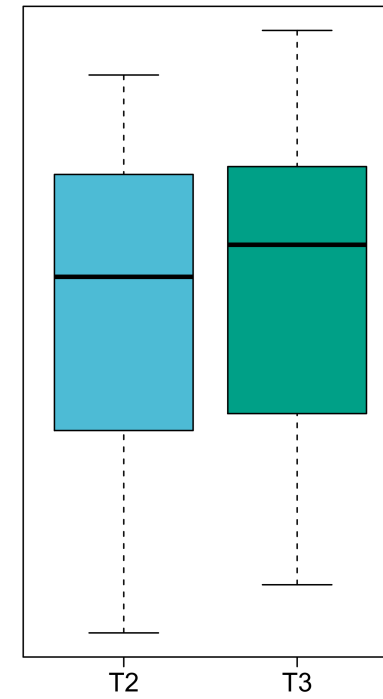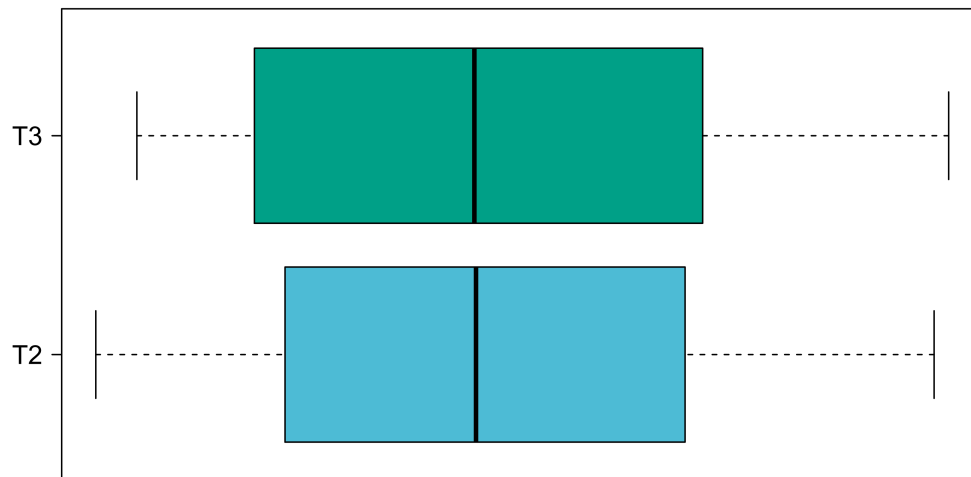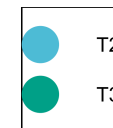

Supplement: Supplementary file 2 — Additional file 2. Comparisons of the Chao1 index of alpha diversity and PCoA of the beta diversity of the gut microbiota during the three trimesters. P1–3: The horizontal axis: the grouping of samples, the Longitudinal axis: Chao1 index. P values are shown on the middle line and in the upper left corner of each chart. P4–6: The horizontal and longitudinal axis represent the first and second principal coordinates, respectively; the percentage indicates the contribution rate of the corresponding principal coordinate to the sample difference; the P value is the test P value of the corresponding principal coordinate; the dots indicate each sample; and different colors indicate that the samples belong to different groups. The horizontal box chart shows the distribution of values of different groups on the first principal coordinate; the vertical box chart shows the distribution of values of different groups on the second principal coordinate. There were neither significant differences in the alpha diversity of the gut microbiota (GM) between T1 and T2 (P1), T1 and T3 (P2), T2 and T3 (P3), nor did the beta diversity of the GM analysis between T1 and T2 (P4), T1 and T3 (P5), T2 and T3 (P6). T1: the first trimester; T2: the second trimester; T3: the third trimester [file 40001_2024_1702_MOESM2_ESM.pdf]

Unweighted Unifrac

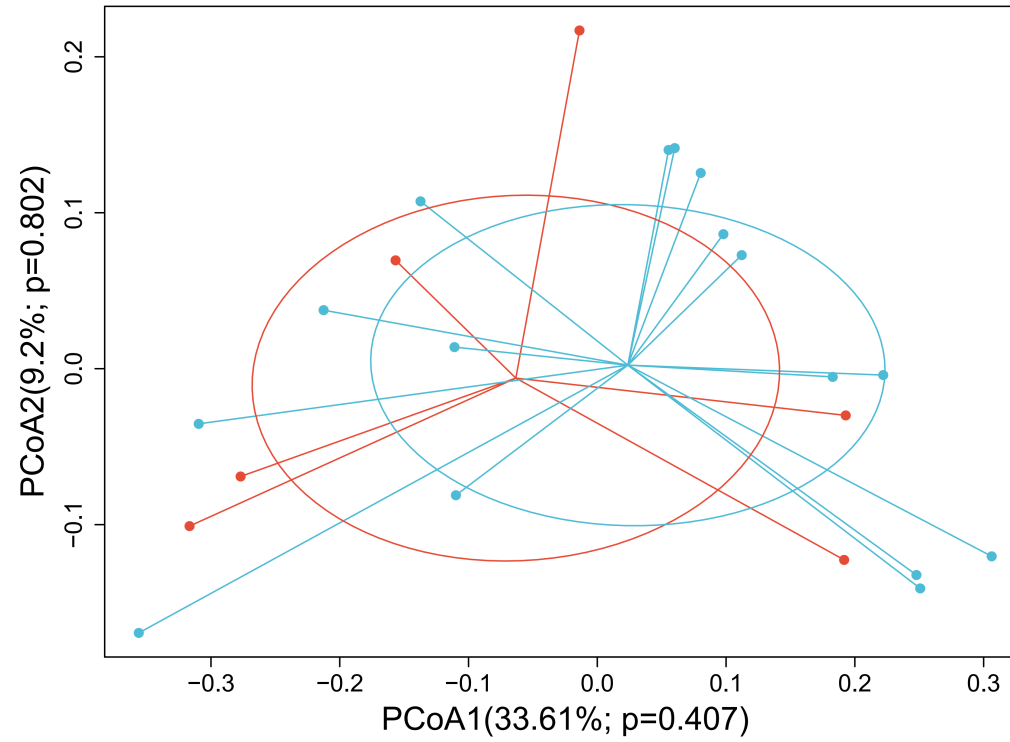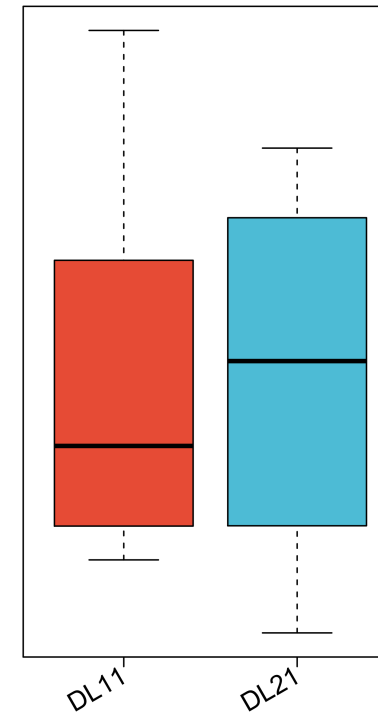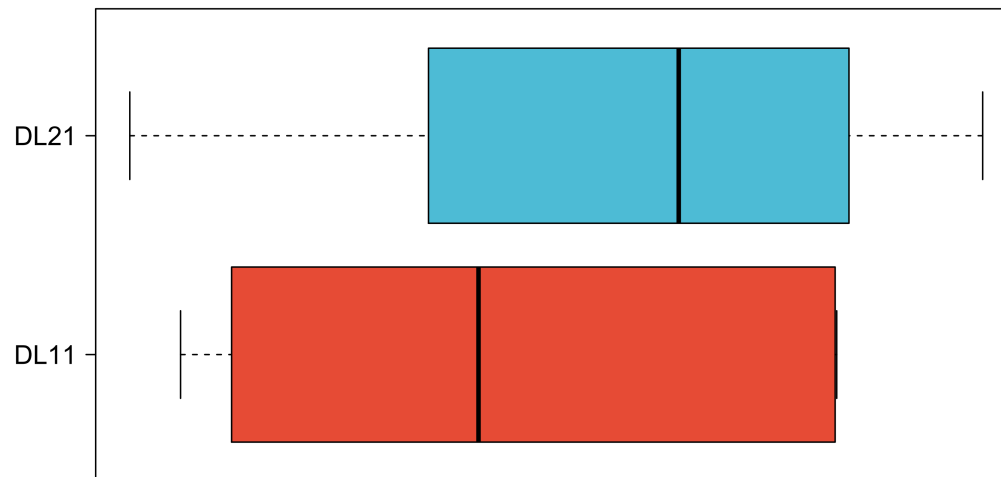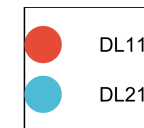

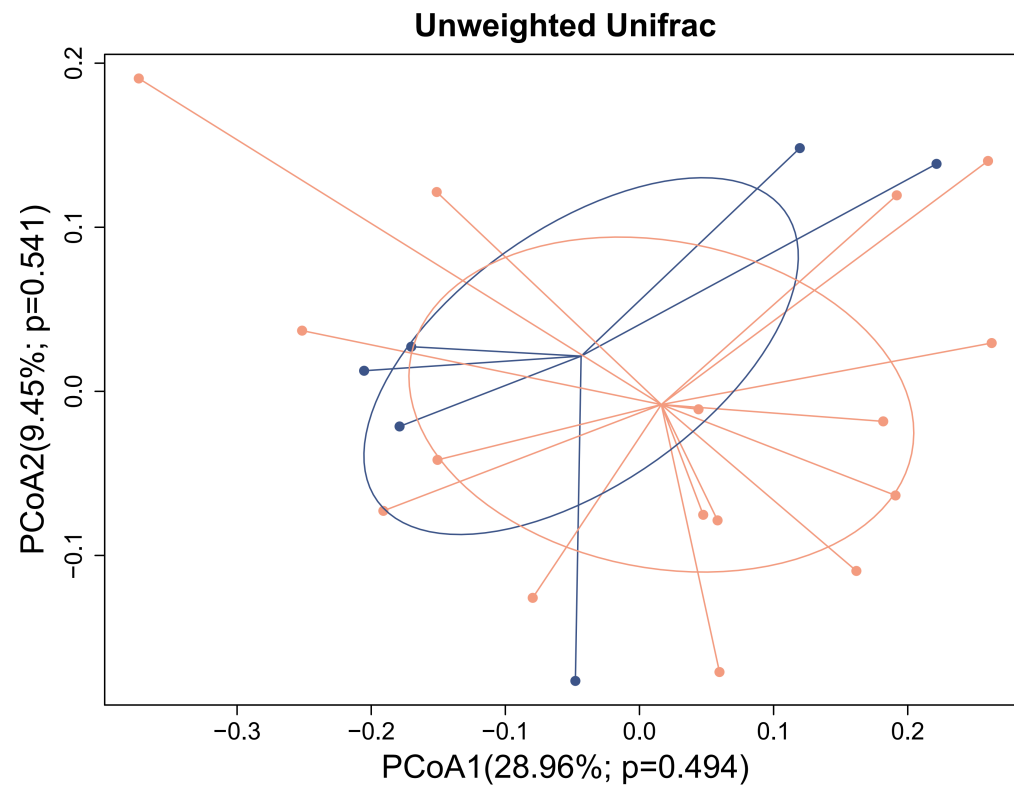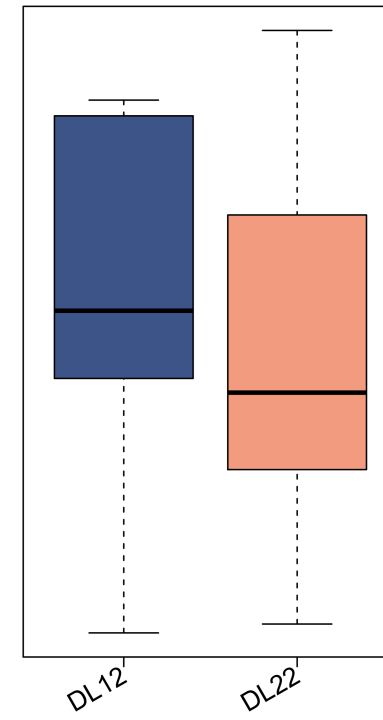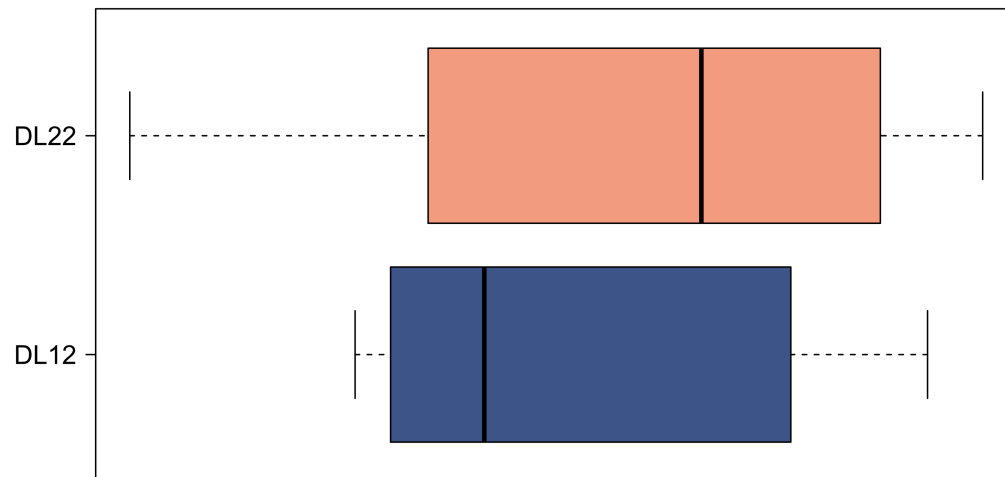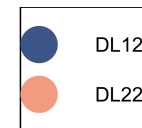

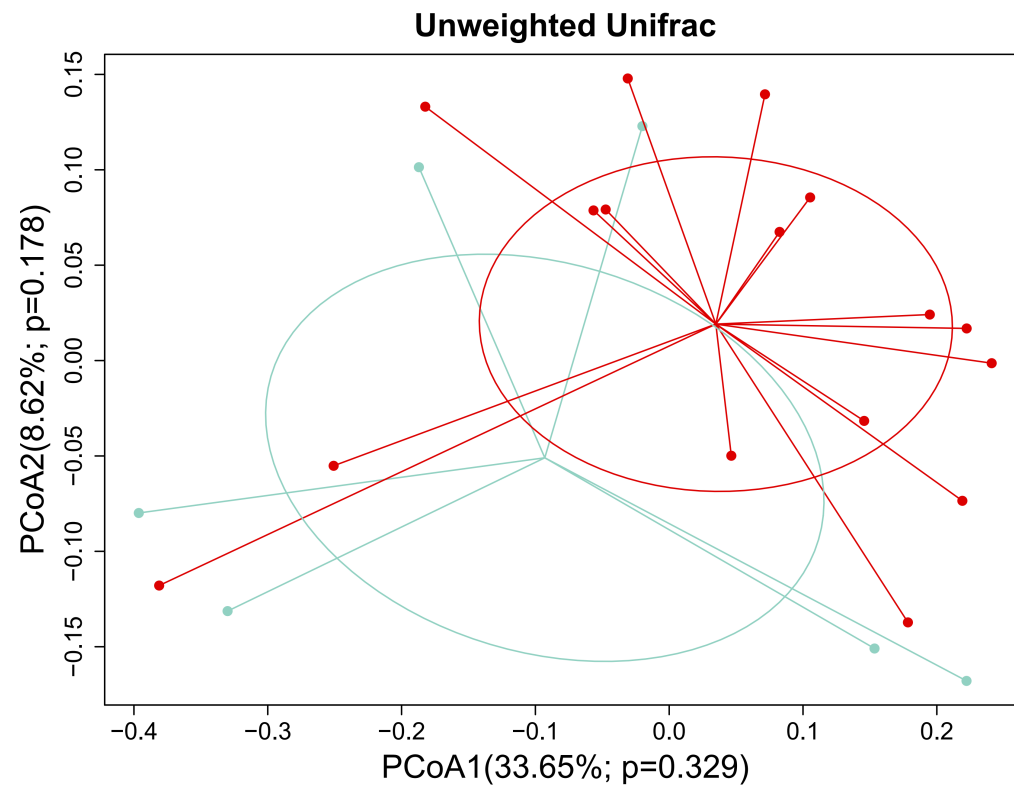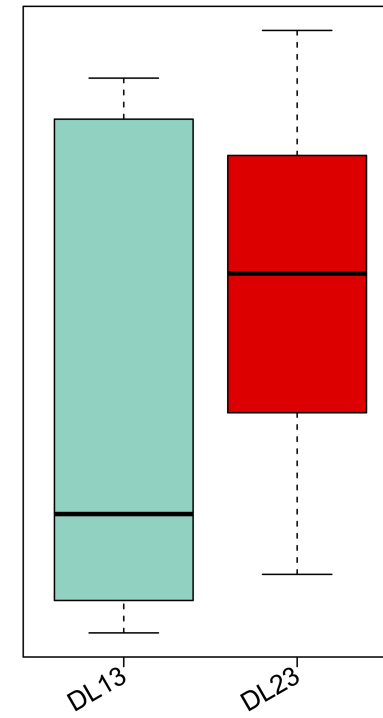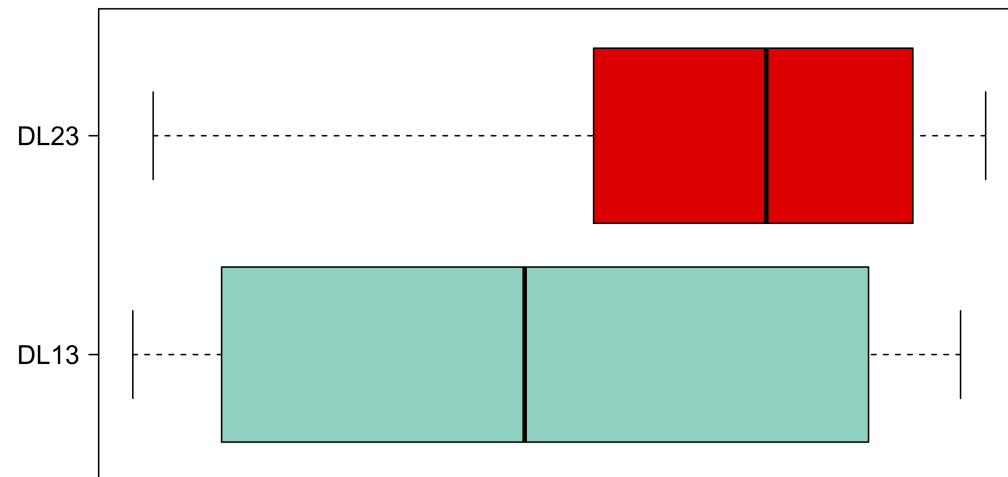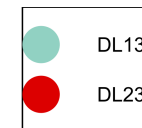

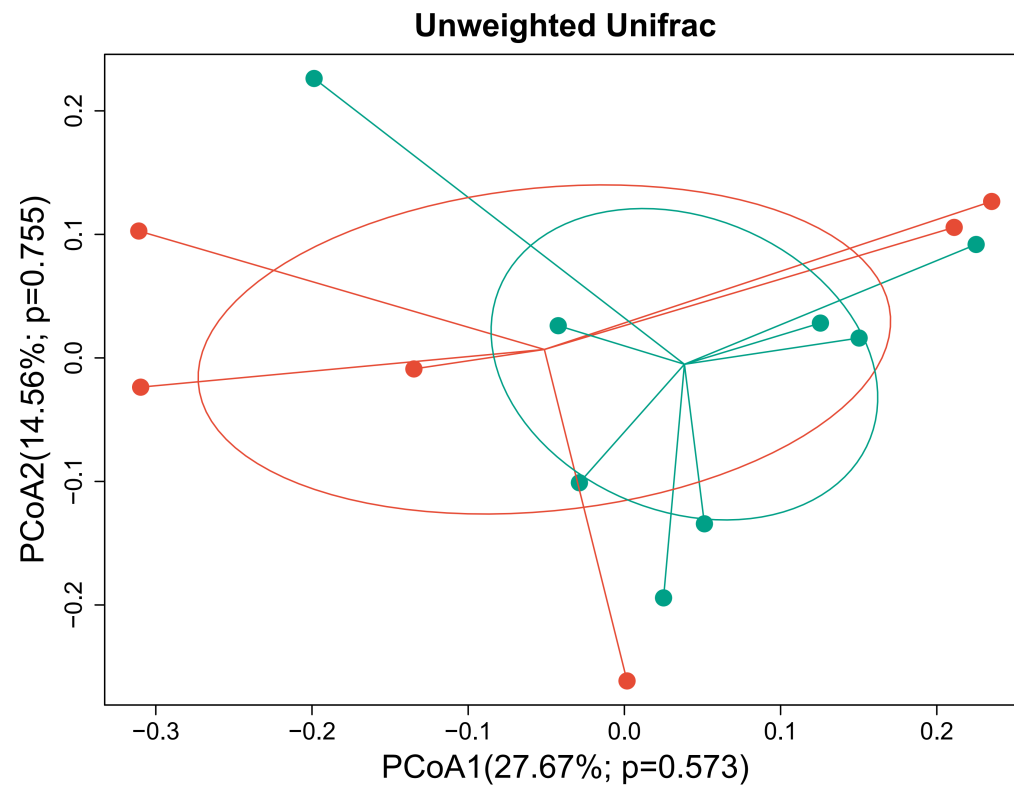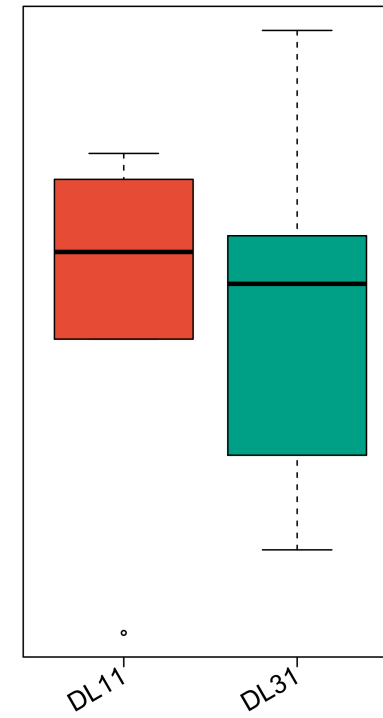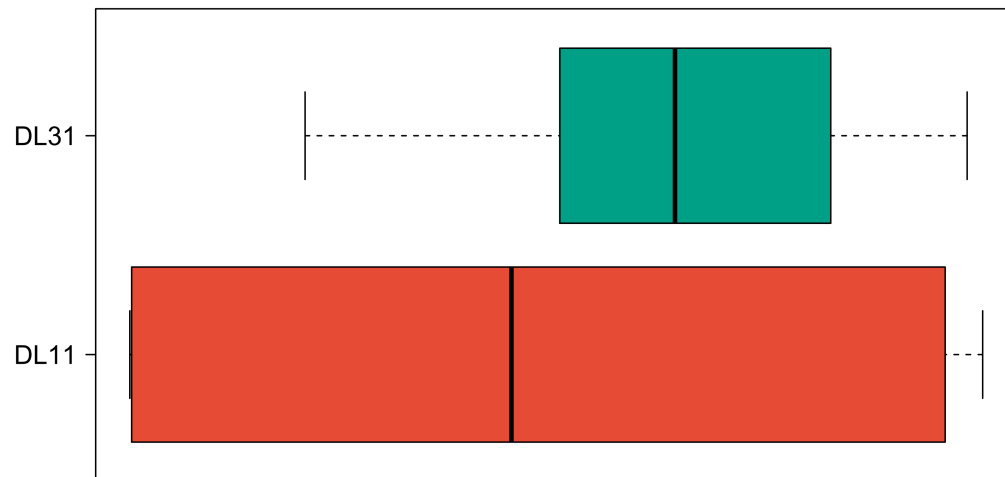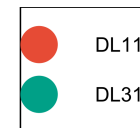

Unweighted Unifrac

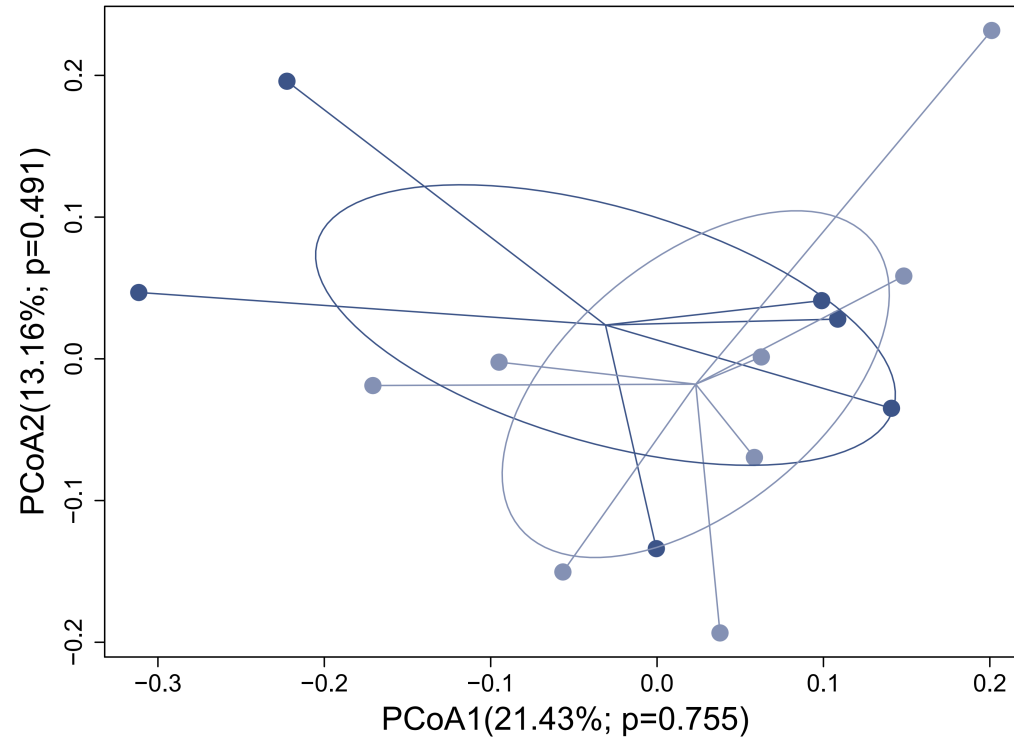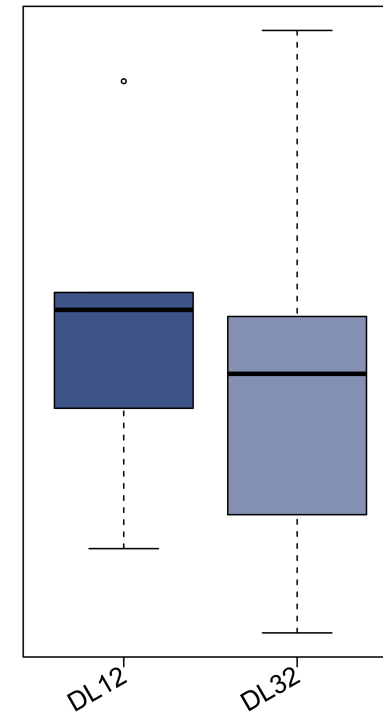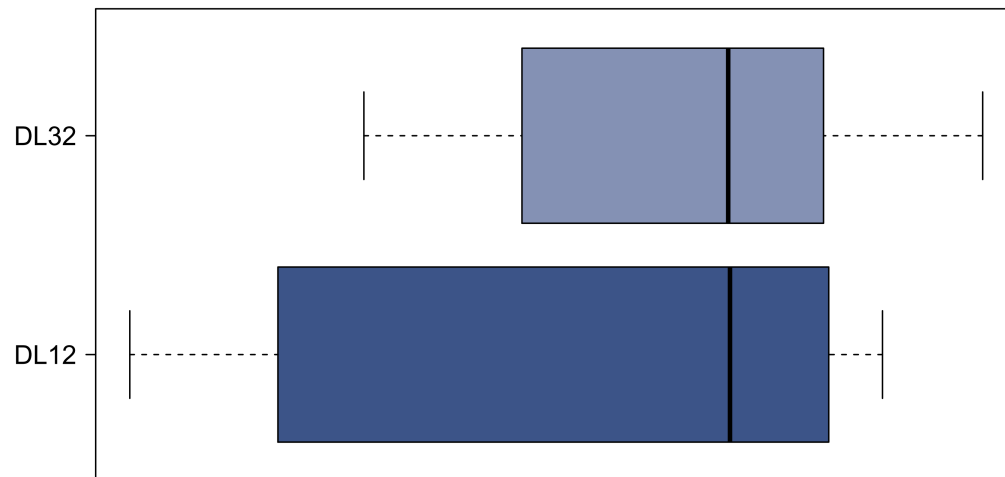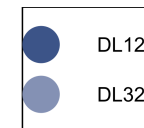

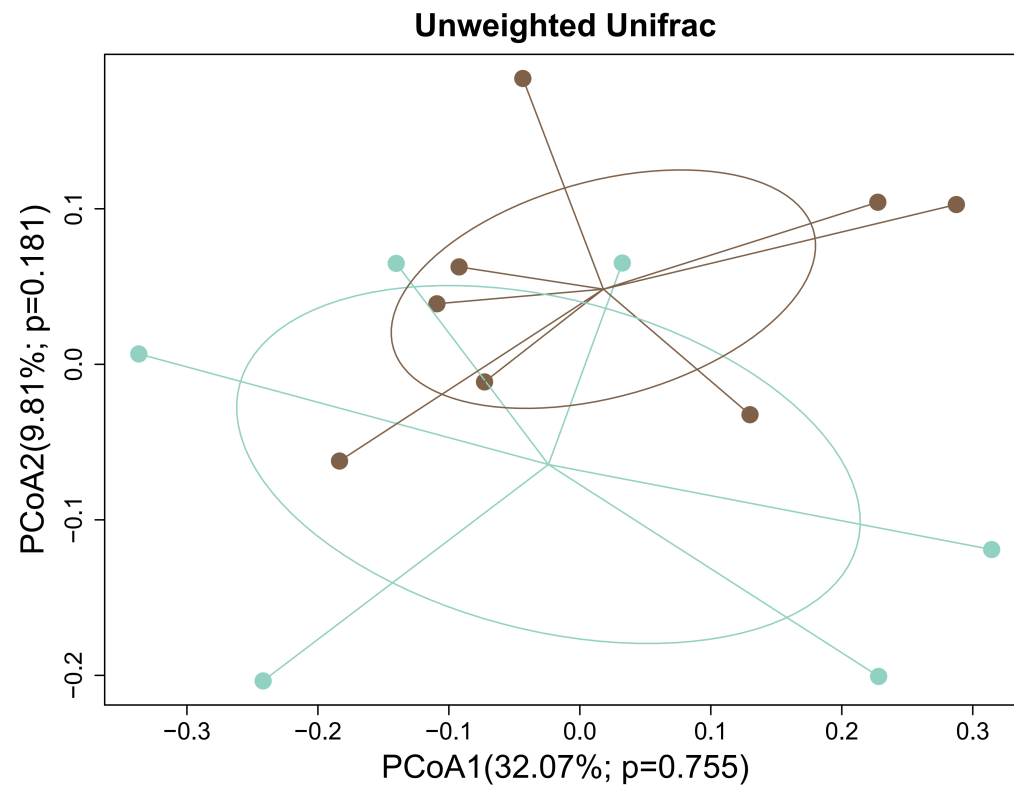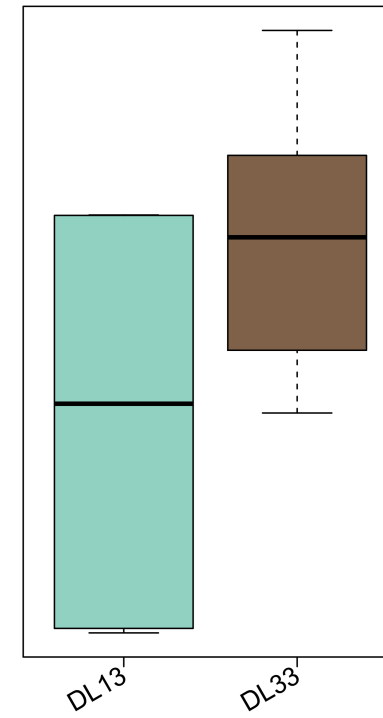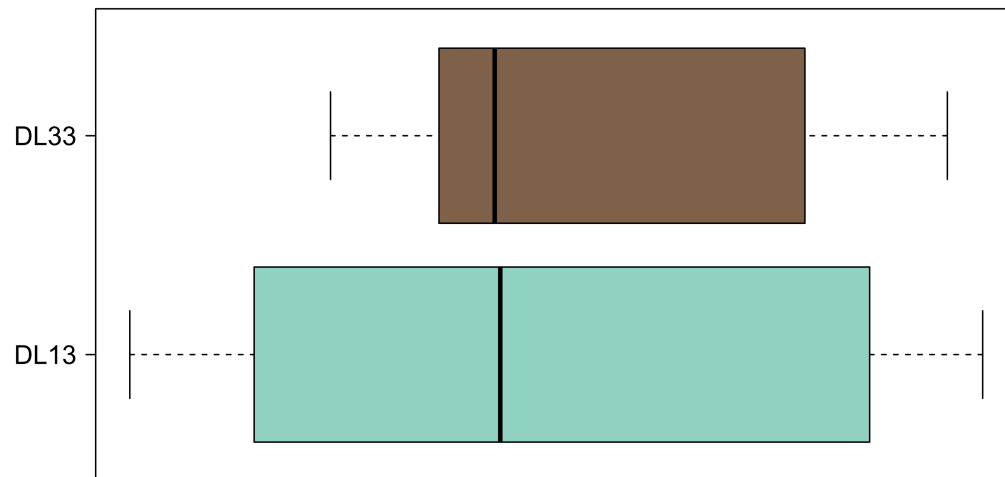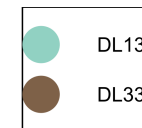

Unweighted Unifrac

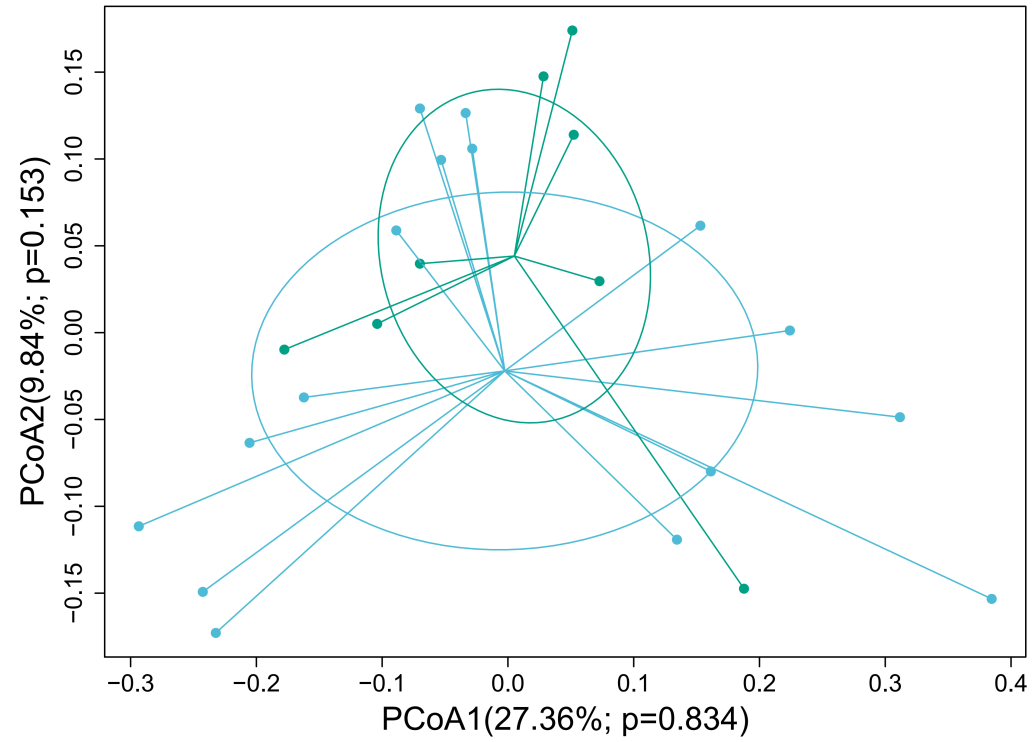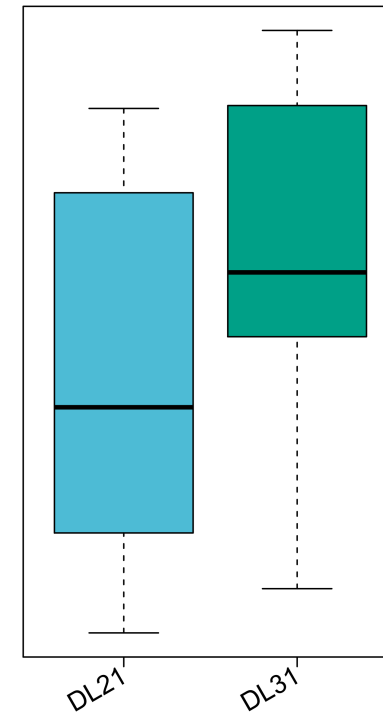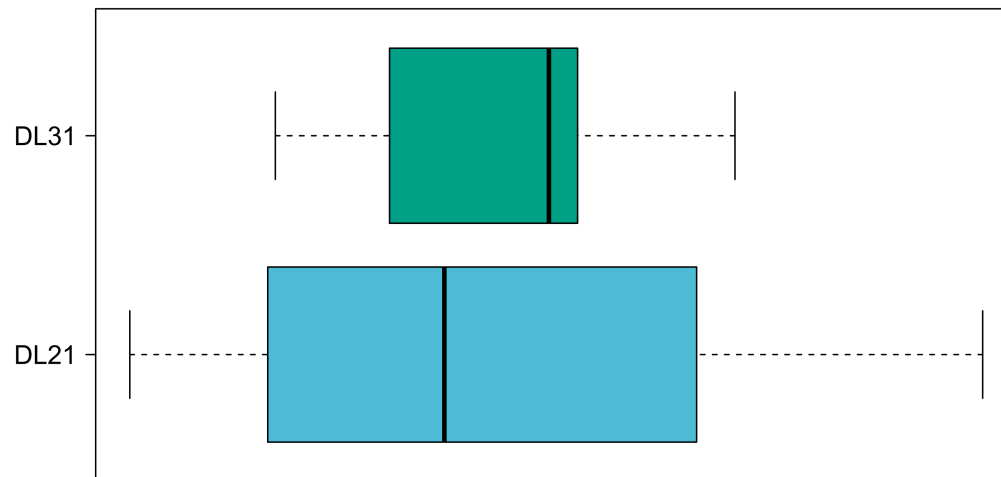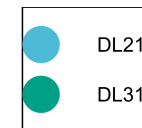

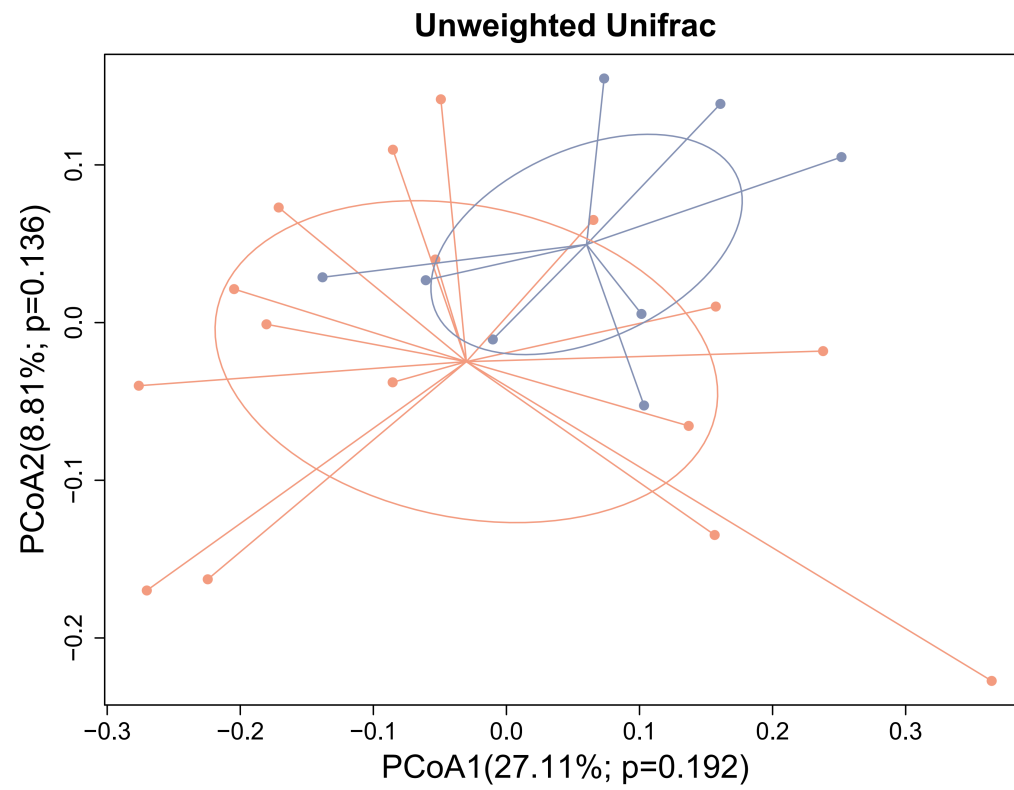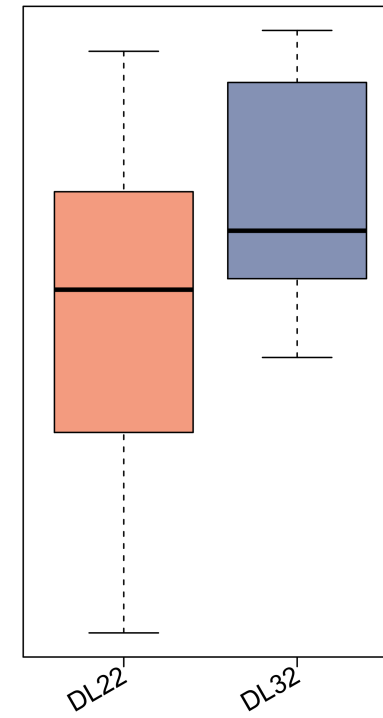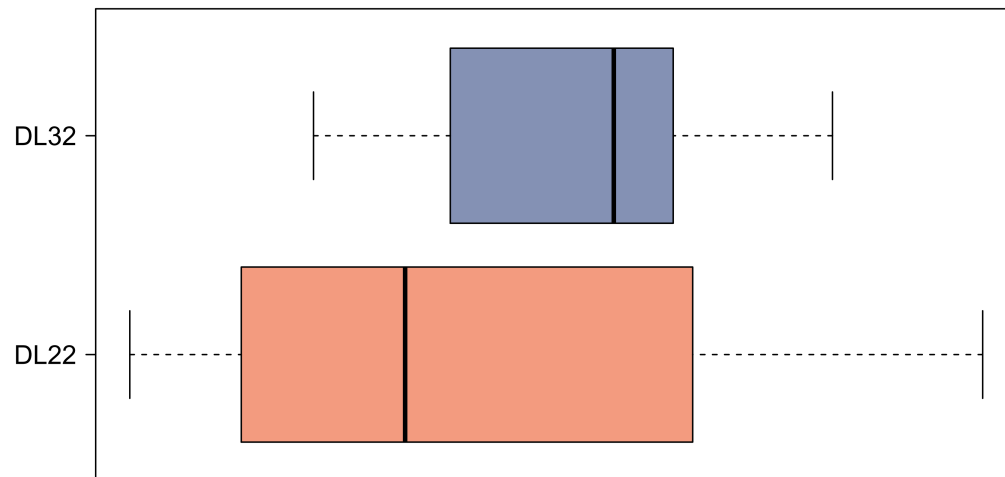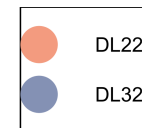

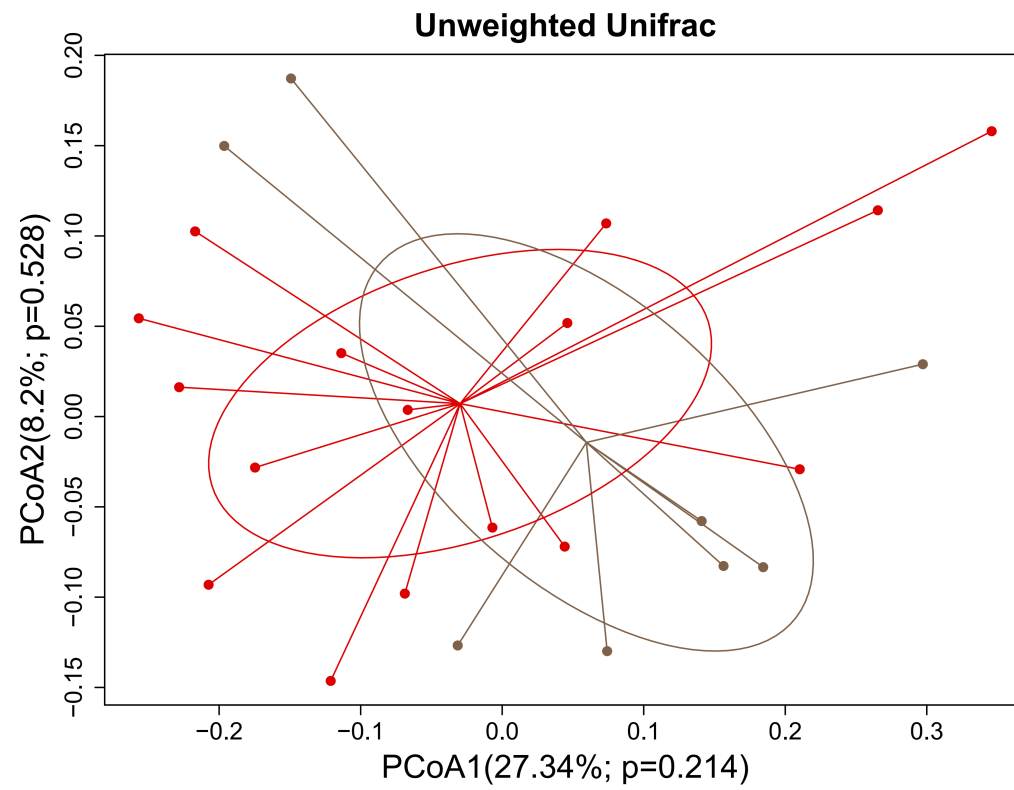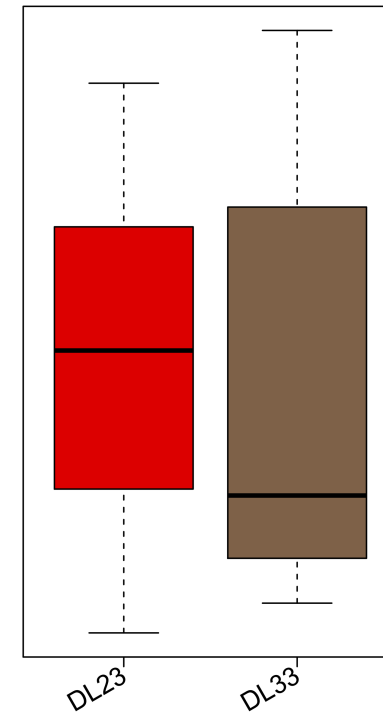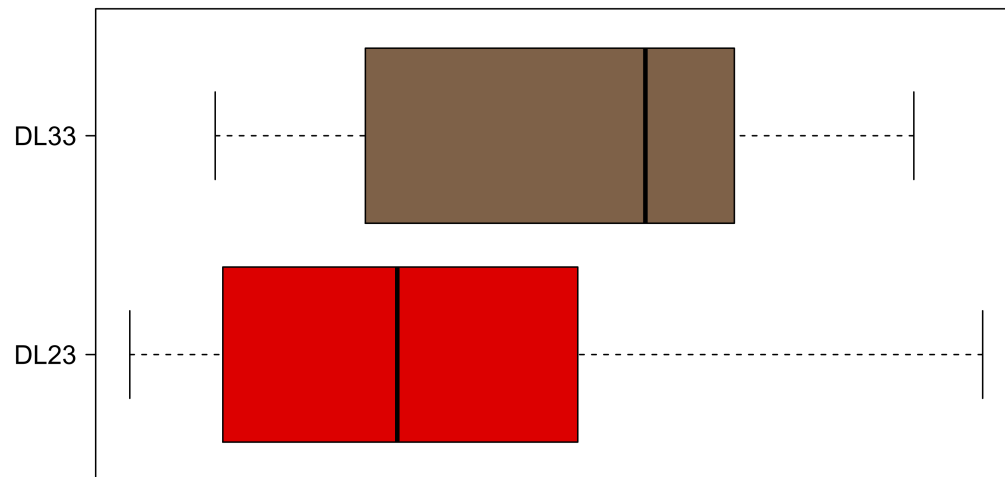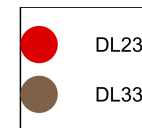

Supplement: Supplementary file 6 — Additional file 6. PCoA of the beta diversity of the gut microbiota in those groups with different onset time of dyslipidemia across the three trimesters. There were no significant differences in the beta diversity of the gut microbiota (GM) between DL1 and DL2 (P1–3), DL1 and DL3(P4–6), DL2 and DL3 (P7–9) in T1, T2 and T3. P value is shown in each chart. All the abbreviations as described in Additional file 5. [file 40001_2024_1702_MOESM6_ESM.pdf]
