# Supplementary material for: Dynamic changes in the gut microbiota during three consecutive trimesters of pregnancy and their correlation with abnormal glucose and lipid metabolism
Source: Eur J Med Res. 2024 Feb 12;29:117. doi: 10.1186/s40001-024-01702-0 (PMC10860297; doi:10.1186/s40001-024-01702-0)
Supplement: Supplementary file 3 — Additional file 3. Comparisons of the Chao1 index of alpha diversity of the gut microbiota in those groups with different onset time of high FBG across the three trimesters. There were no significant differences in the alpha diversity of the gut microbiota (GM) between WNG and ENG (P1–3), WNG and LNG (P4–6), WNG and WHG (P7–9), WHG and ENG (P10–12) and WHG and LNG (P13–15) in T1, T2 and T3. P value is shown in each chart. T1: the first trimester; T2: the second trimester; T3: the third trimester; WNG: normal FBG during the whole pregnancy, ENG: normal FBG in the early stage (T1) of pregnancy, LNG: normal FBG in the late stage (T3) of pregnancy, WHG: high FBG during the whole pregnancy. [file 40001_2024_1702_MOESM3_ESM.pdf]

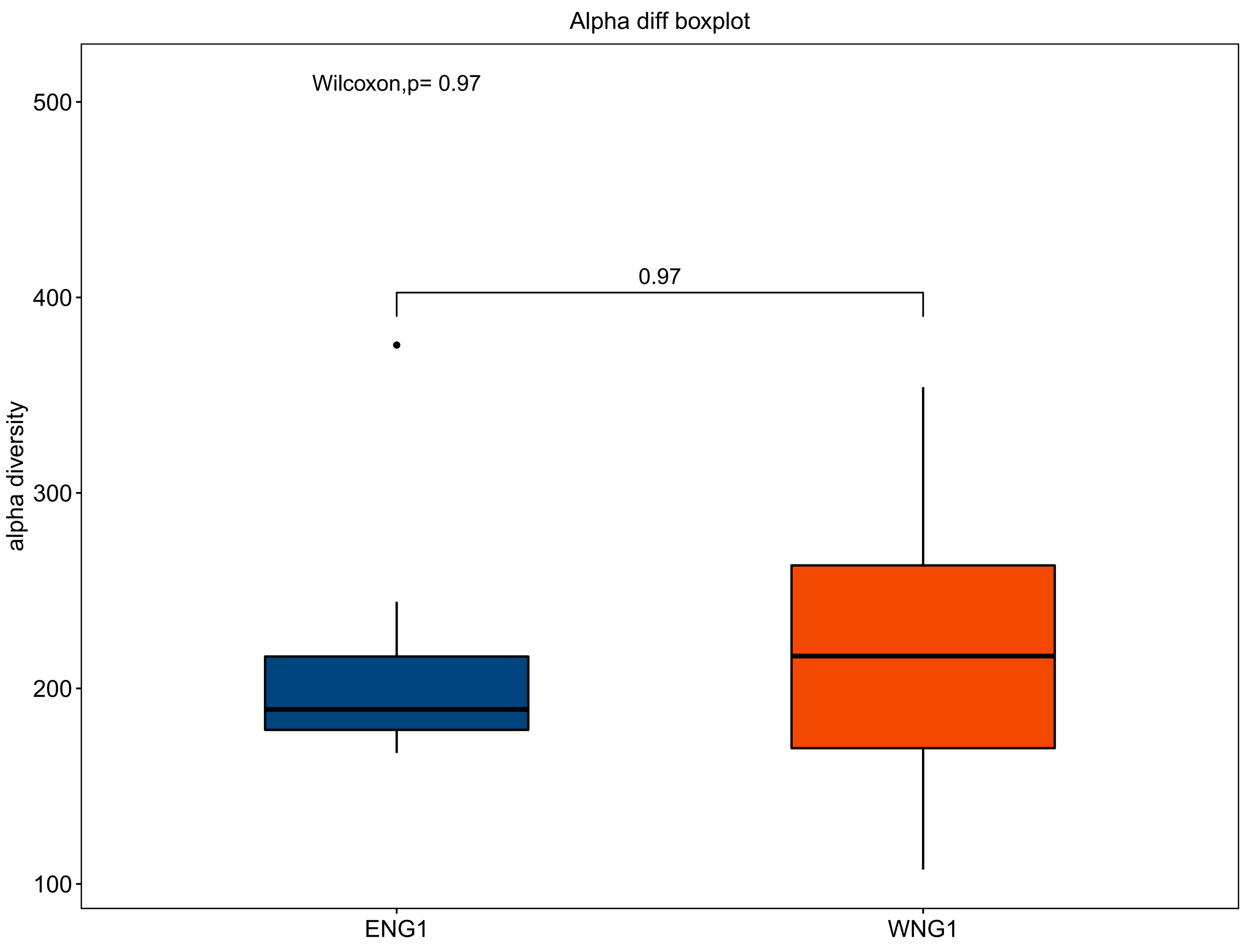

Alpha diff boxplot
Wilcoxon,p= 0.97
0.97
alpha diversity
500
400
300
200
100
ENG1
WNG1

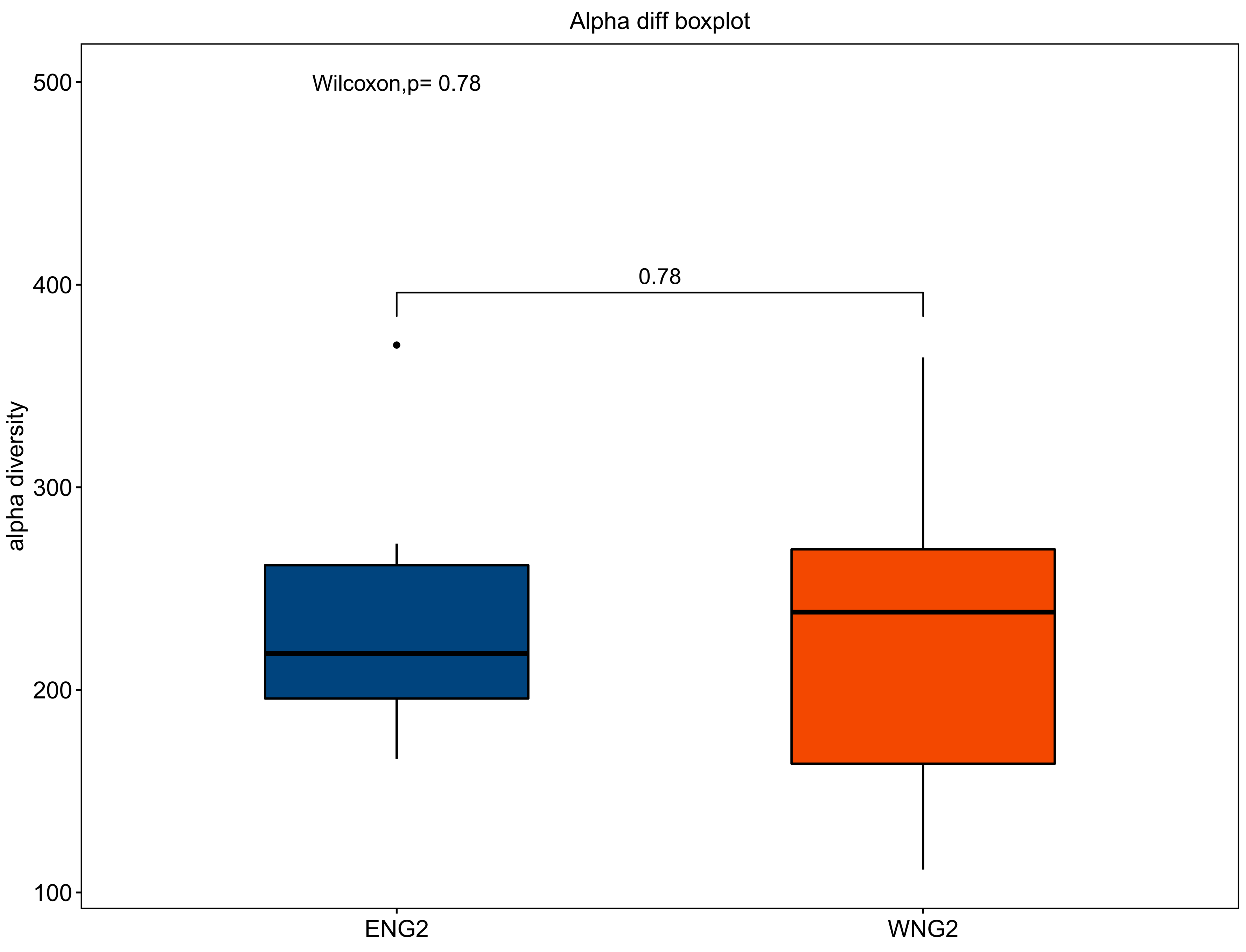
Alpha diff boxplot
Wilcoxon,p= 0.78
0.78
alpha diversity
500
400
300
200
100
ENG2
WNG2

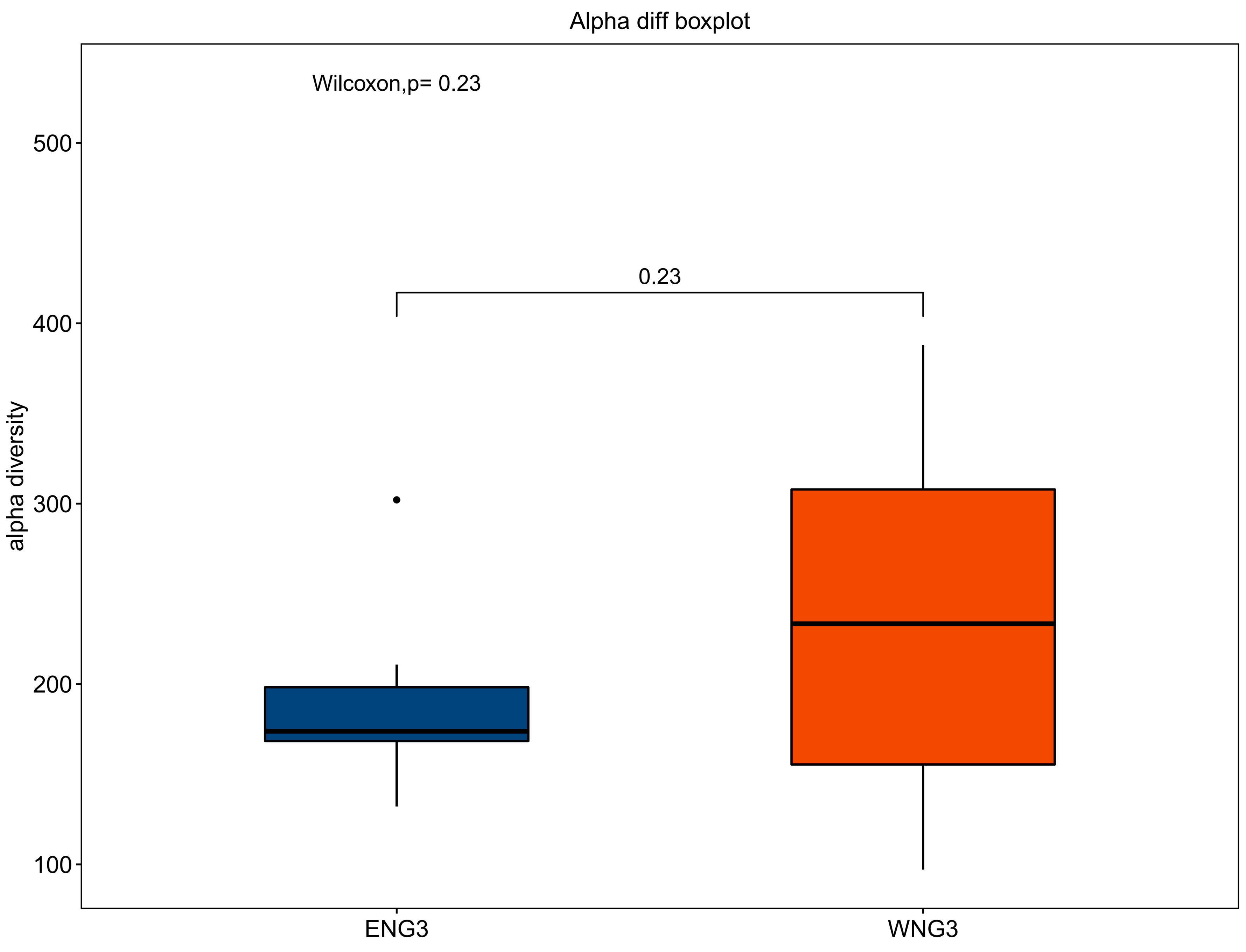
Alpha diff boxplot
Wilcoxon,p= 0.23
0.23
500
400
300
200
100
alpha diversity
ENG3
WNG3

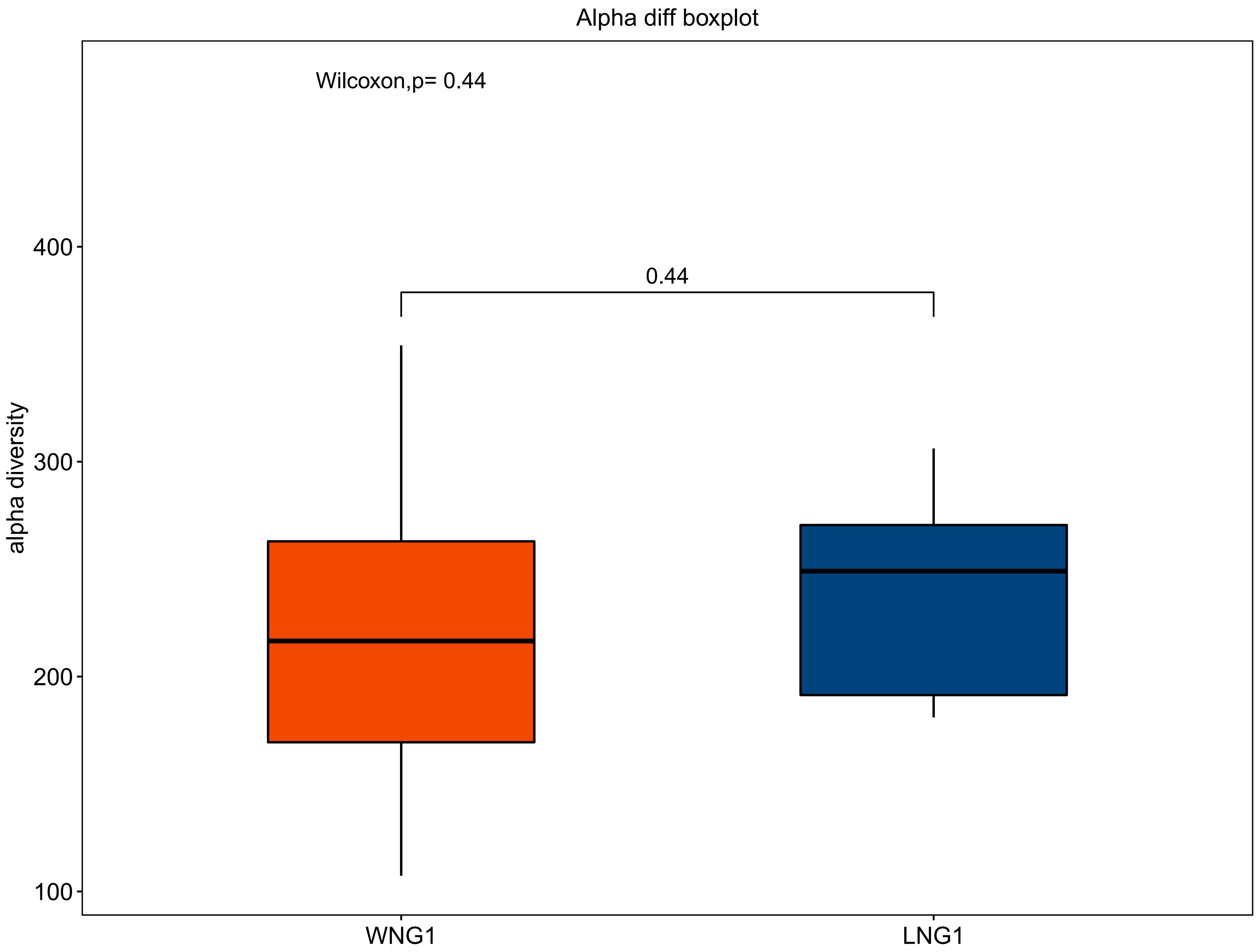
Alpha diff boxplot
Wilcoxon,p= 0.44
0.44
alpha diversity
400
300
200
100
WNG1
LNG1

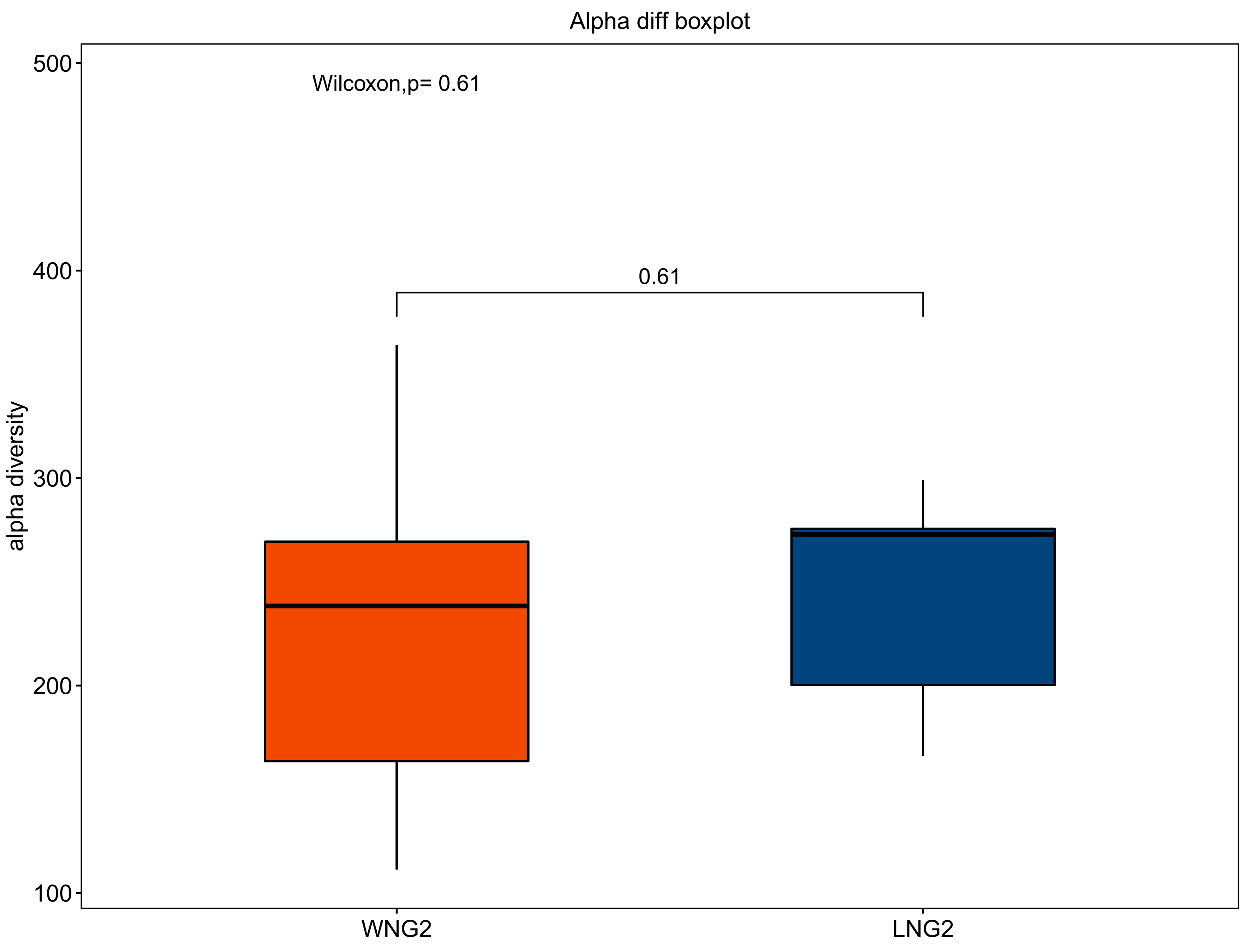
Alpha diff boxplot
Wilcoxon,p= 0.61
0.61
alpha diversity
500
400
300
200
100
WNG2
LNG2

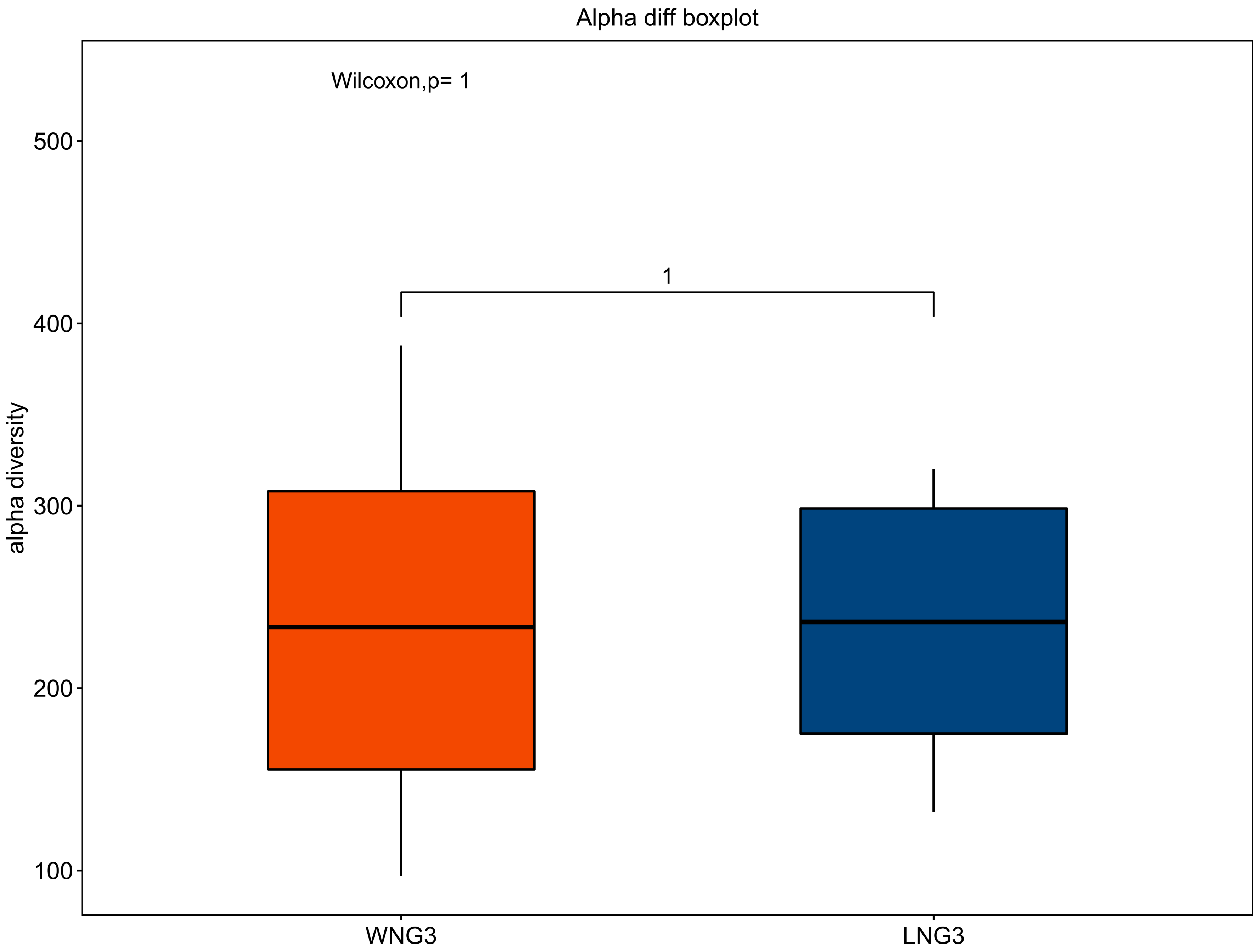
Alpha diff boxplot
Wilcoxon,p= 1
1
500
400
300
200
100
alpha diversity
WNG3
LNG3

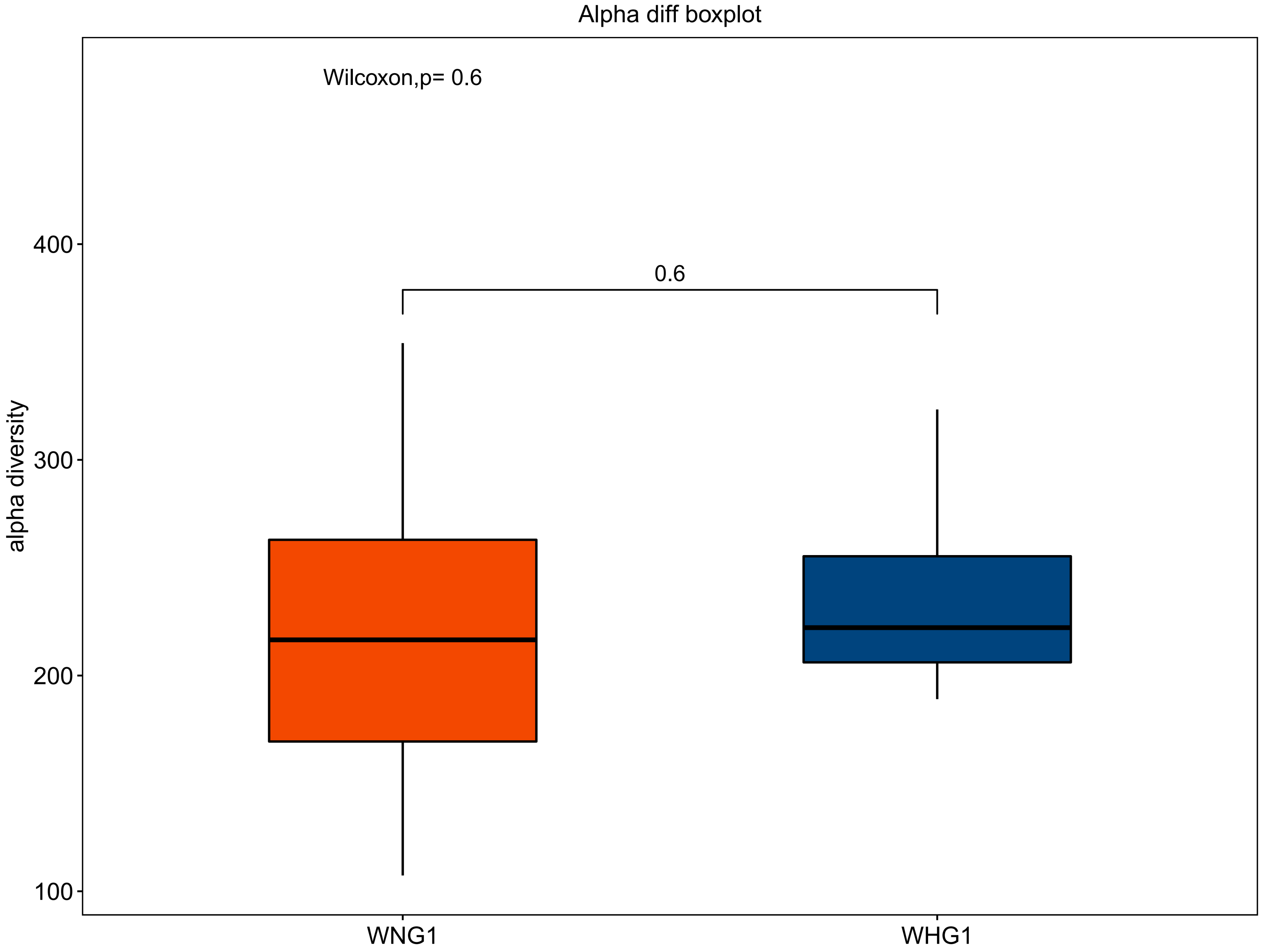
Alpha diff boxplot
Wilcoxon,p= 0.6
0.6
alpha diversity
400
300
200
100
WNG1
WHG1

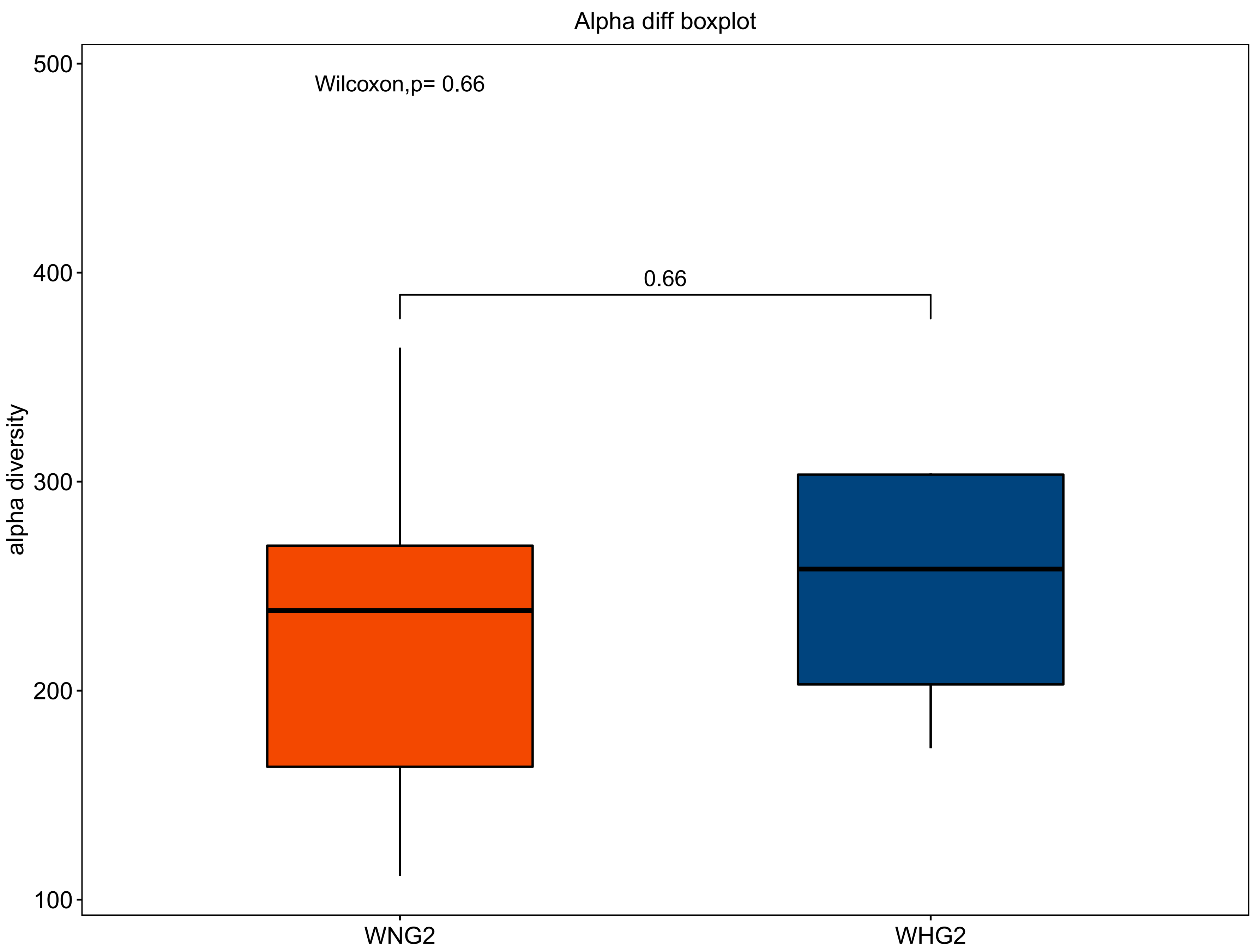

Alpha diff boxplot
Wilcoxon,p= 0.66
0.66
500
400
300
200
100
alpha diversity
WNG2
WHG2

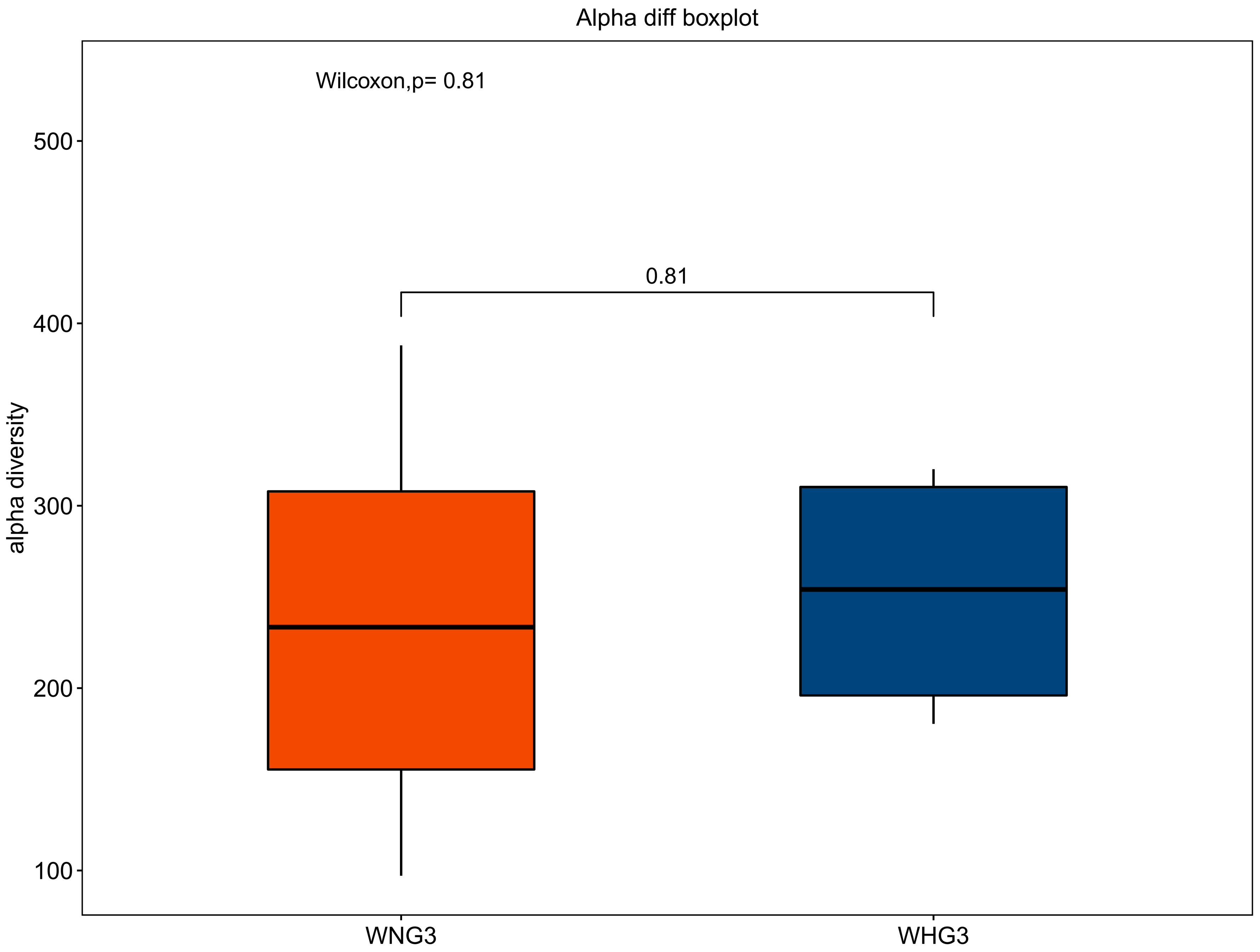
Alpha diff boxplot
Wilcoxon,p= 0.81
0.81
alpha diversity
500
400
300
200
100
WNG3
WHG3

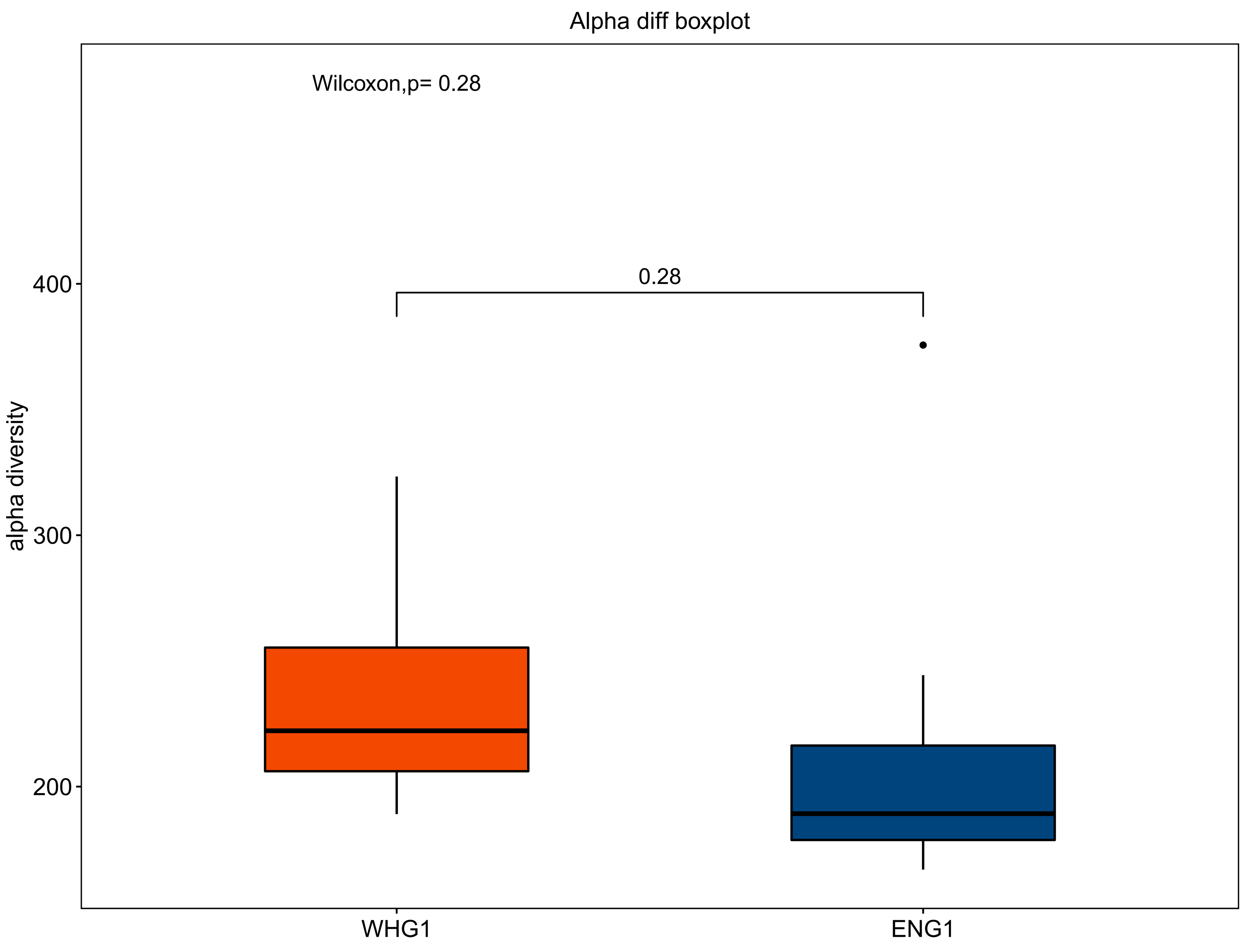
Alpha diff boxplot
Wilcoxon,p= 0.28
0.28
alpha diversity
400
300
200
WHG1
ENG1

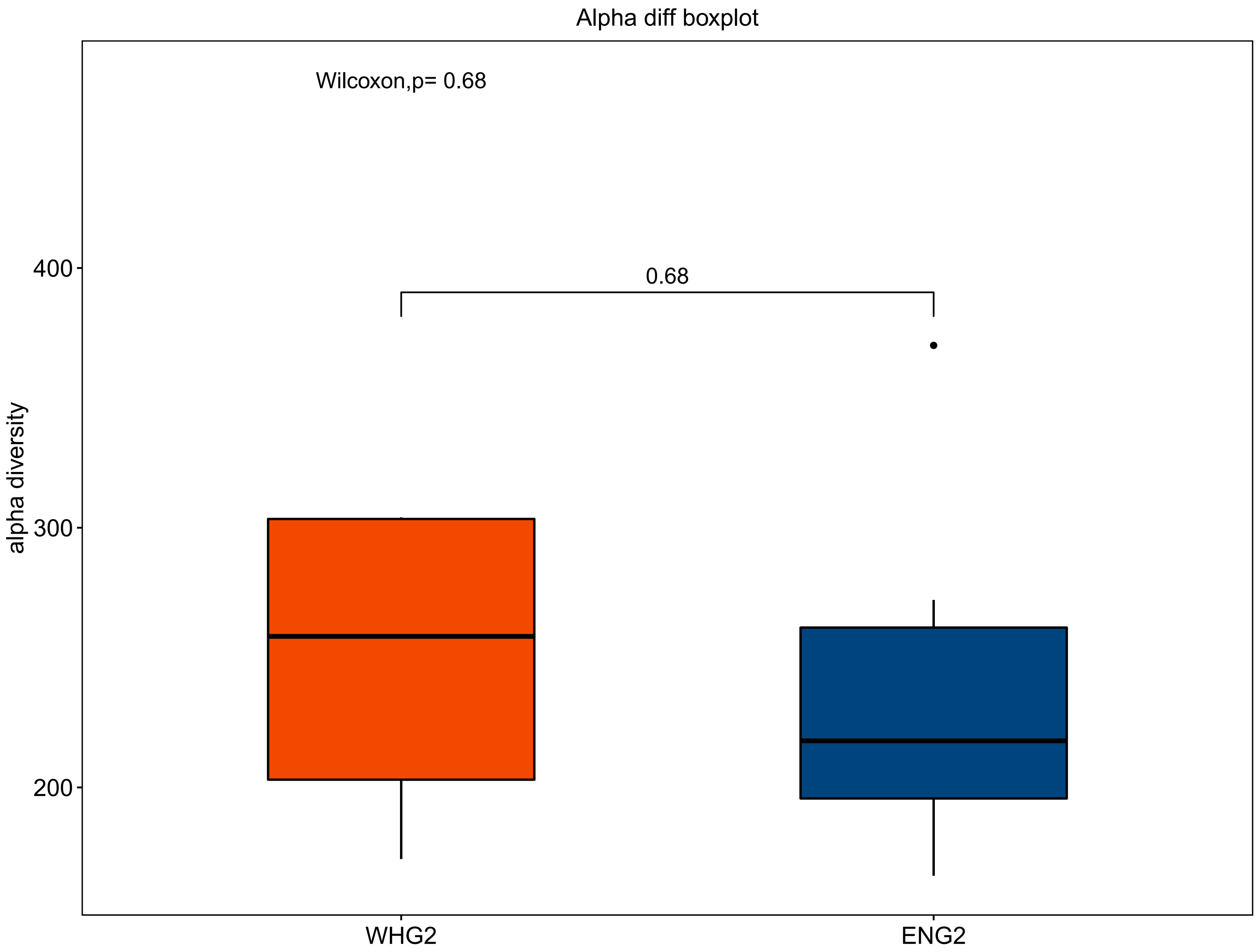
Alpha diff boxplot
Wilcoxon,p= 0.68
0.68
alpha diversity
400
300
200
WHG2
ENG2

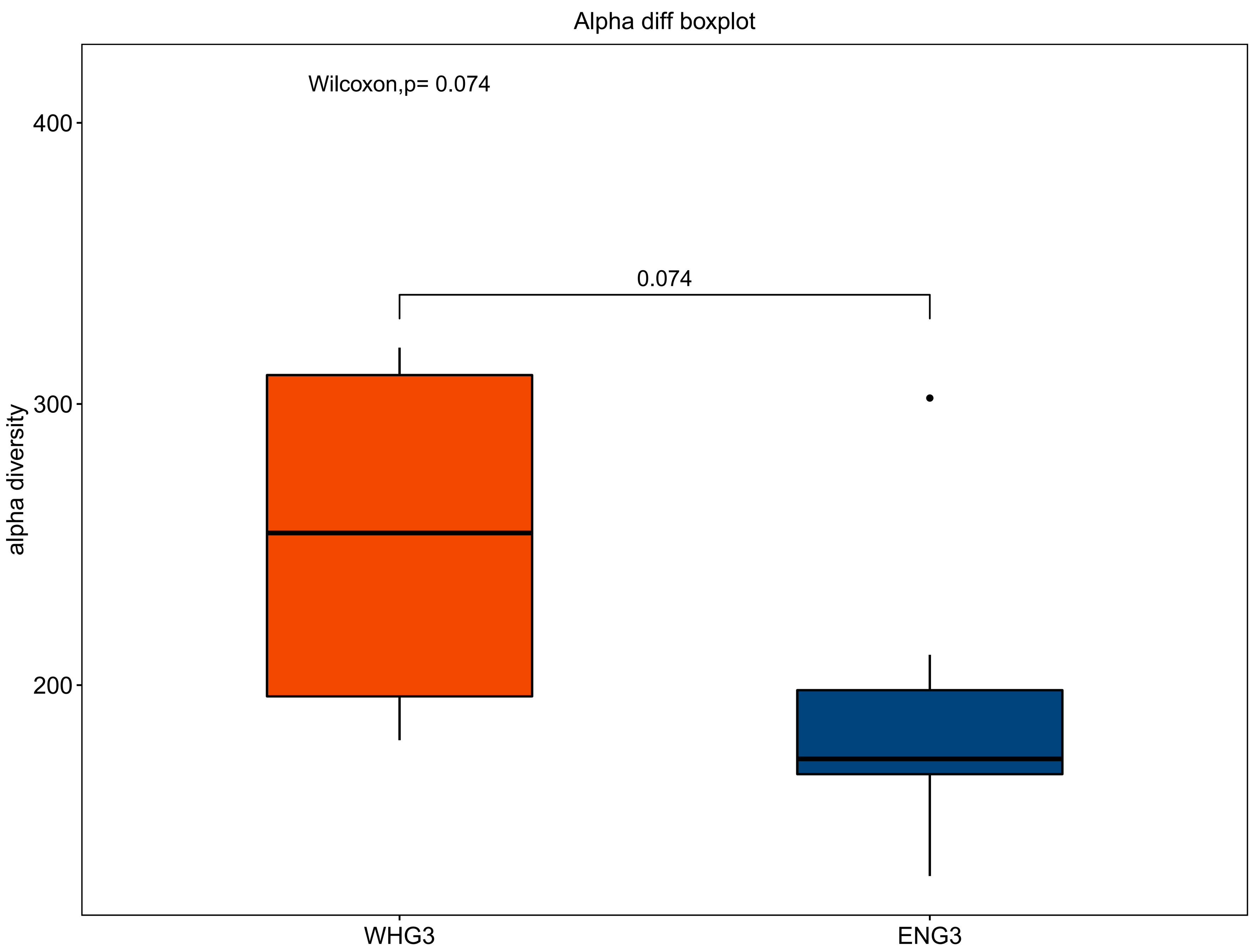
Alpha diff boxplot
Wilcoxon,p= 0.074
0.074
400
300
200
alpha diversity
WHG3
ENG3

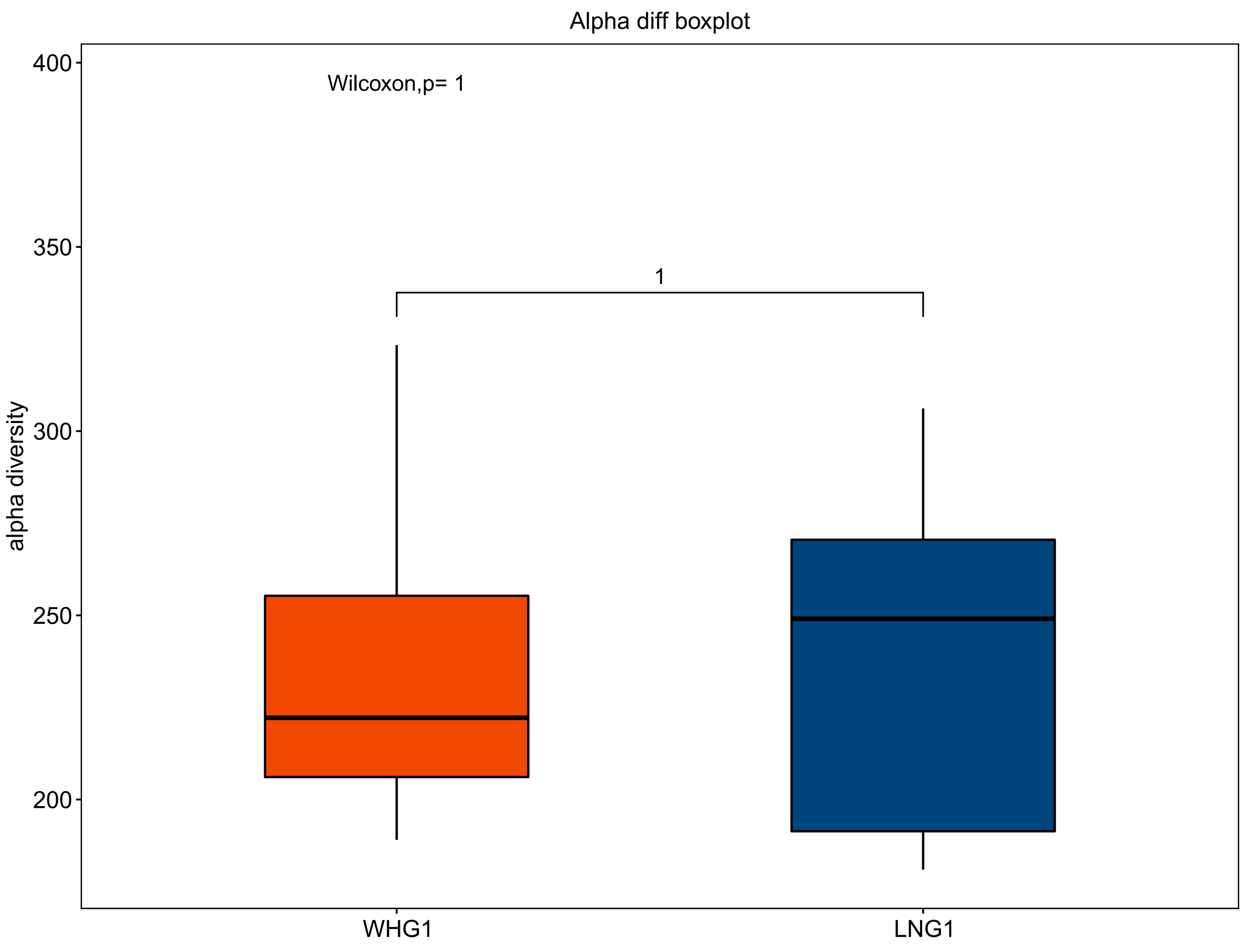
Alpha diff boxplot
Wilcoxon,p= 1
1
400
350
300
250
200
alpha diversity
WHG1
LNG1

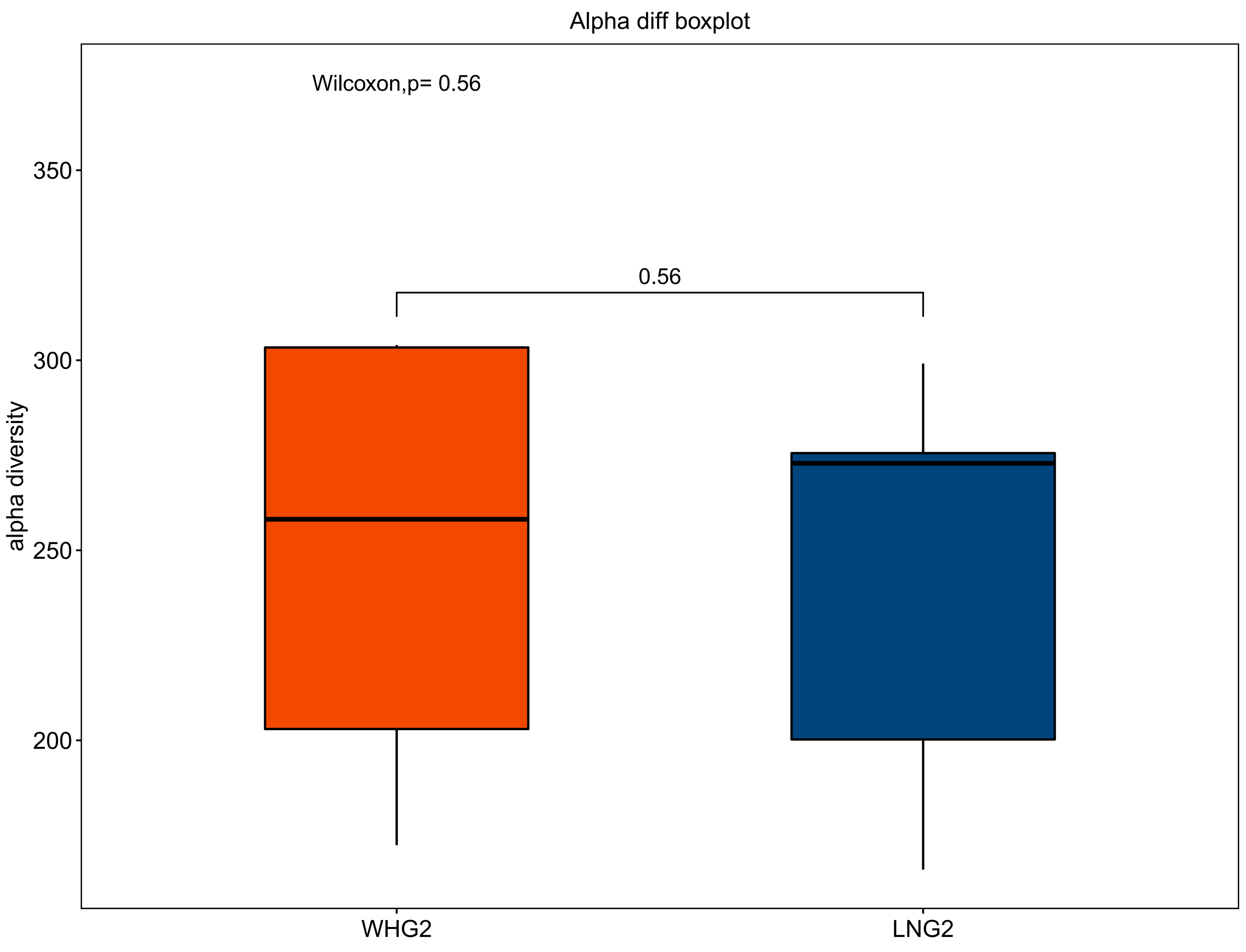
Alpha diff boxplot
Wilcoxon,p= 0.56
0.56
alpha diversity
350
300
250
200
WHG2
LNG2

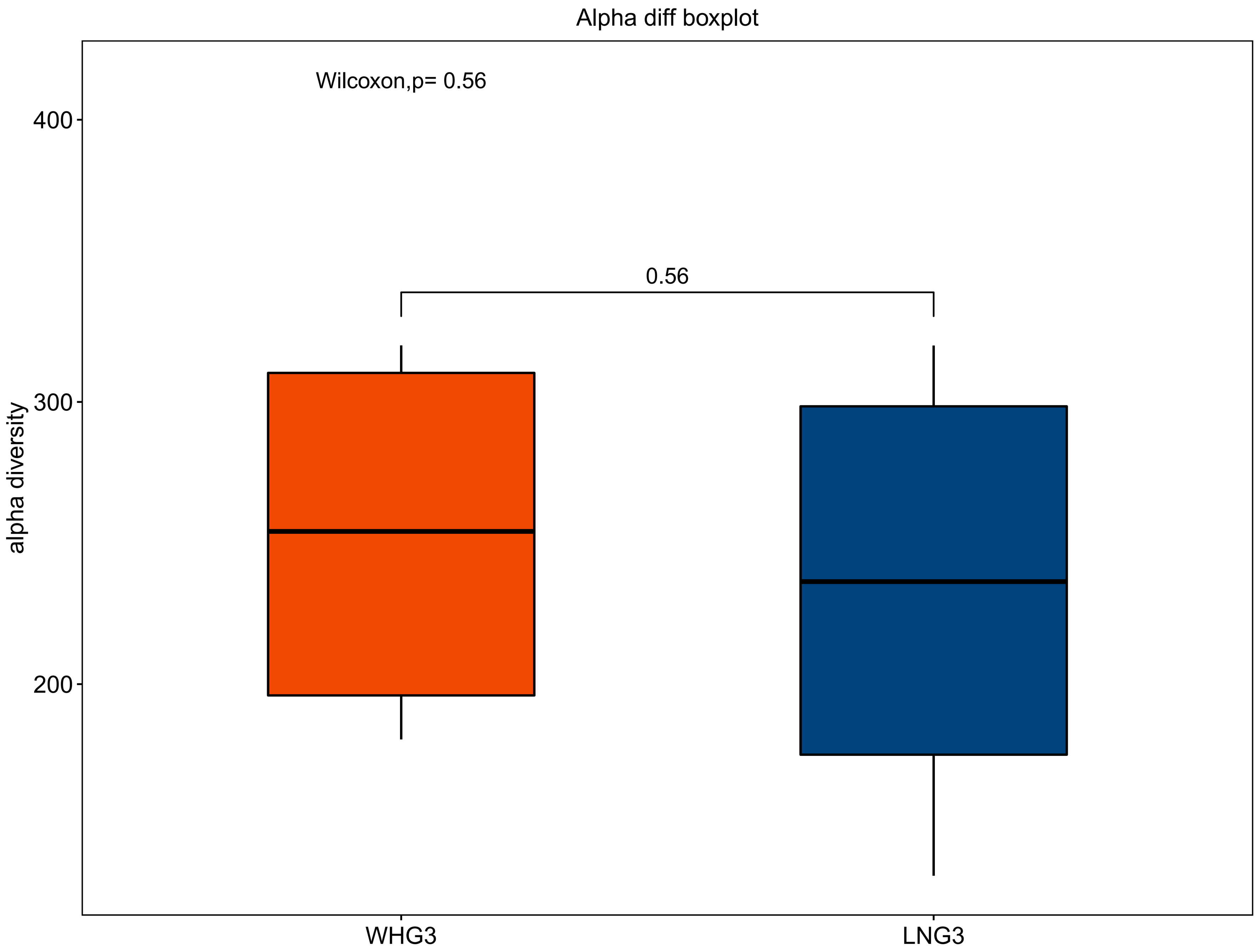

Alpha diff boxplot
Wilcoxon,p= 0.56
0.56
400
300
200
alpha diversity
WHG3
LNG3
