# Supplementary material for: Dynamic changes in the gut microbiota during three consecutive trimesters of pregnancy and their correlation with abnormal glucose and lipid metabolism
Source: Eur J Med Res. 2024 Feb 12;29:117. doi: 10.1186/s40001-024-01702-0 (PMC10860297; doi:10.1186/s40001-024-01702-0)
Supplement: Supplementary file 4 — Additional file 4: PCoA of the beta diversity of the gut microbiota in those groups with different onset time of high FBG across the three trimesters. There were no significant differences in the beta diversity of the gut microbiota (GM) between WNG and ENG (P1–3), WNG and LNG (P4–6), WNG and WHG (P7–9), WHG and ENG (P10–12) and WHG and LNG (P13–15) in T1, T2 and T3. P value is shown in each chart. All the abbreviations as described in Additional file 3. [file 40001_2024_1702_MOESM4_ESM.pdf]

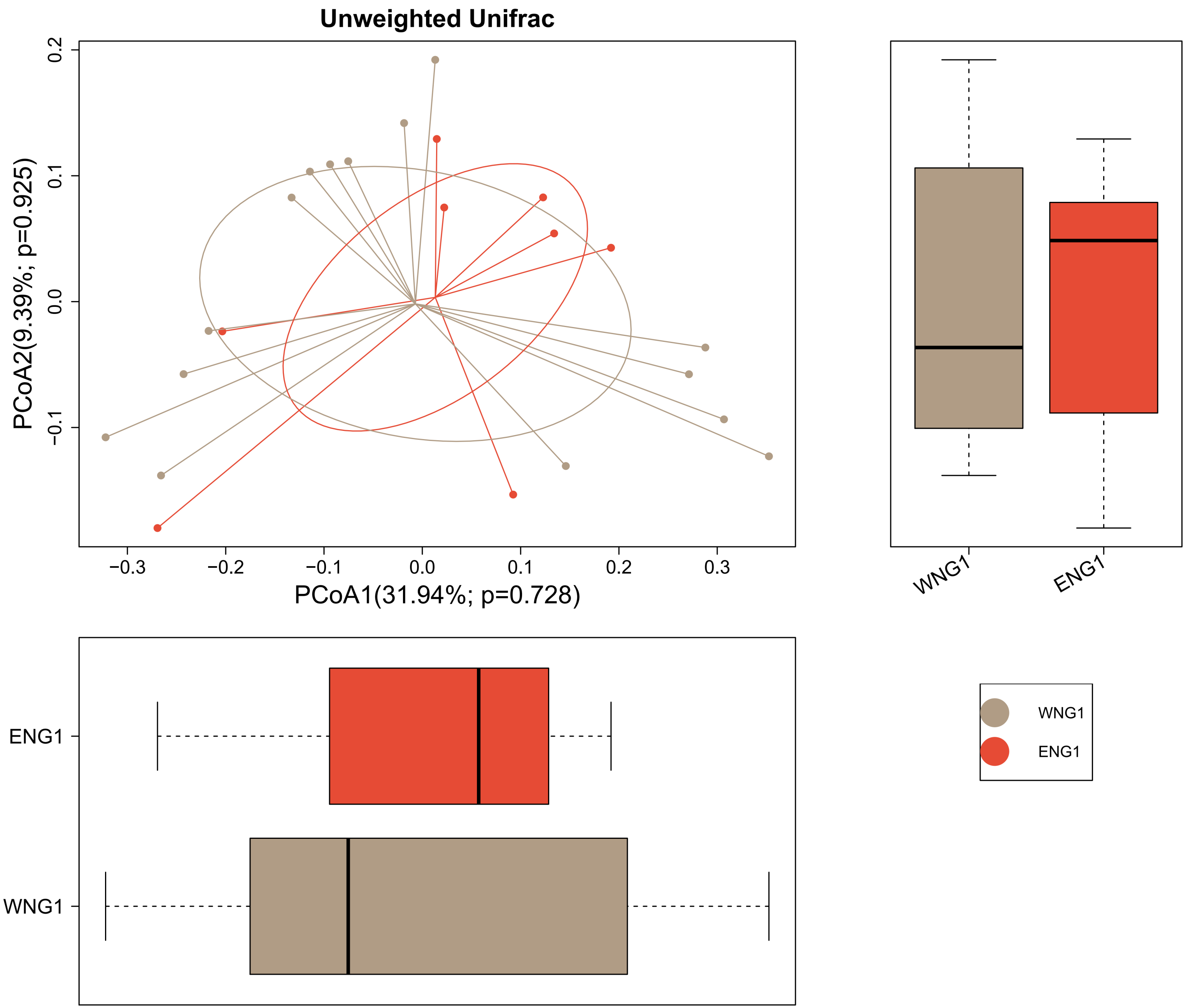

Unweighted Unifrac
PCoA2(9.39%; p=0.925)
PCoA1(31.94%; p=0.728)
−0.3
−0.2
−0.1
0.0
0.1
0.2
0.3
−0.1
0.0
0.1
0.2
WNG1
ENG1
WNG1
ENG1
ENG1
WNG1

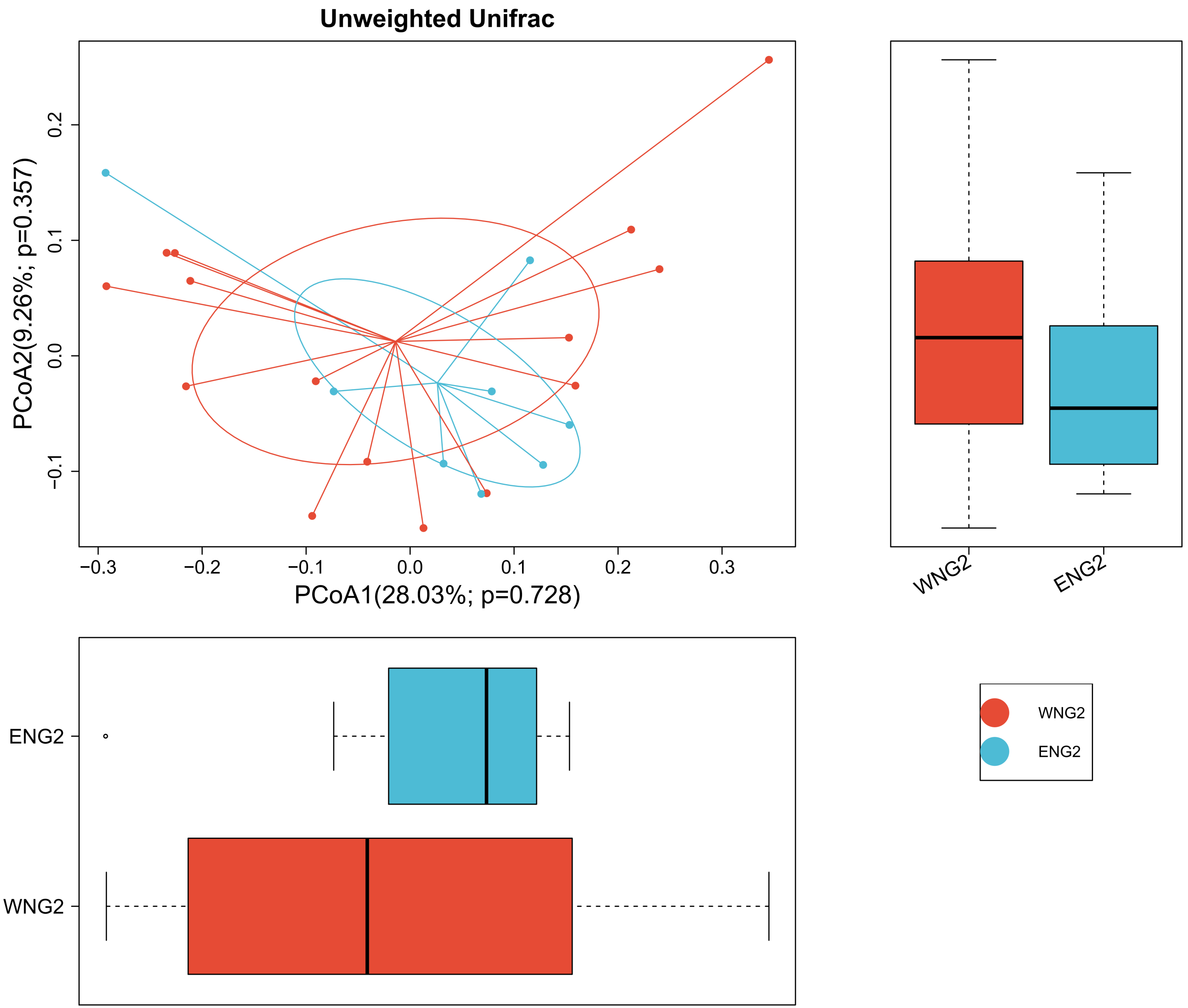
Unweighted Unifrac
PCoA2(9.26%; p=0.357)
PCoA1(28.03%; p=0.728)
−0.3
−0.2
−0.1
0.0
0.1
0.2
0.3
−0.1
0.0
0.1
0.2
WNG2
ENG2
ENG2
WNG2
WNG2
ENG2

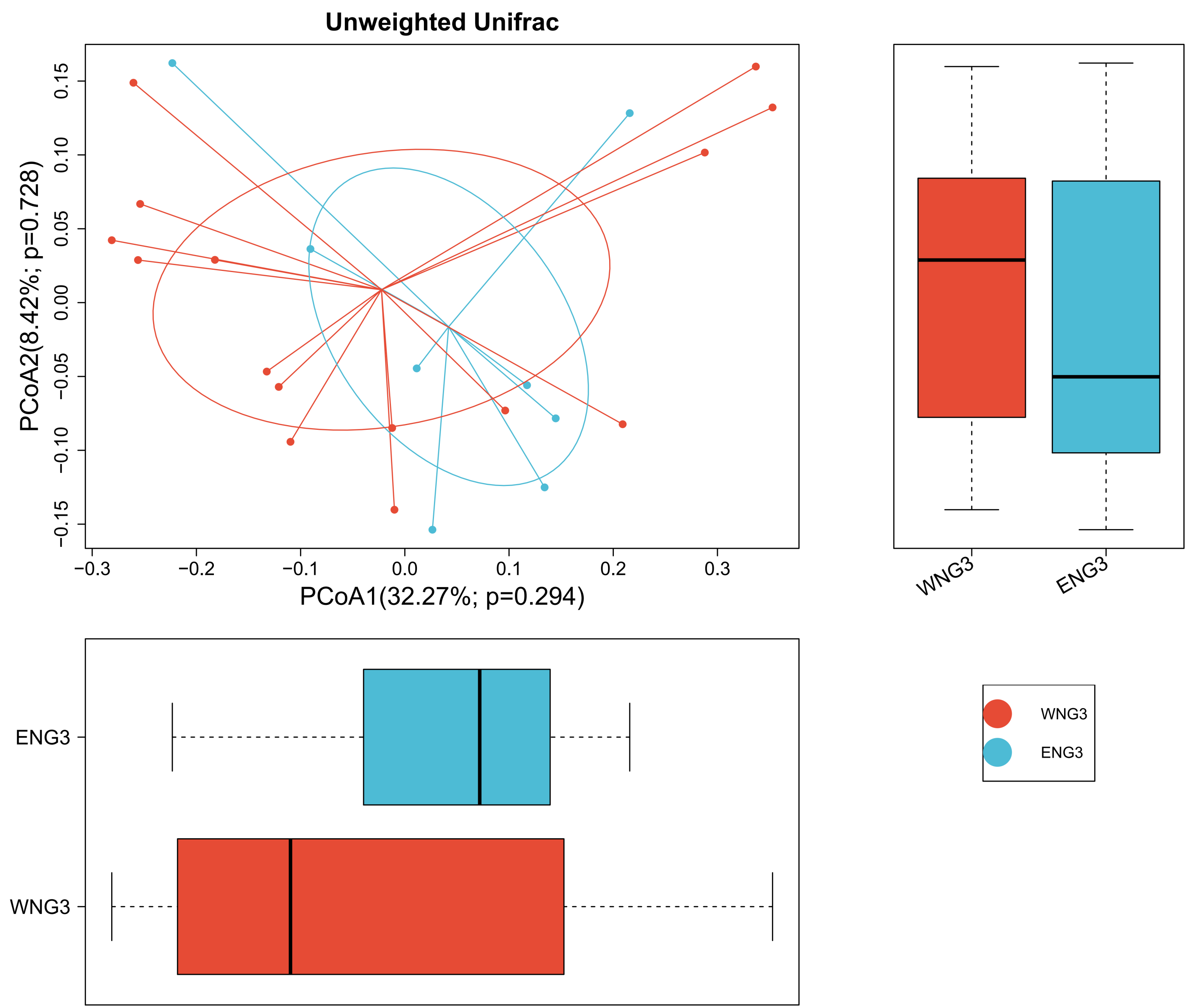

Unweighted Unifrac
PCoA2(8.42%; p=0.728)
PCoA1(32.27%; p=0.294)
0.15
0.10
0.05
0.00
−0.05
−0.10
−0.15
−0.3
−0.2
−0.1
0.0
0.1
0.2
0.3
WNG3
ENG3
ENG3
WNG3
WNG3
ENG3

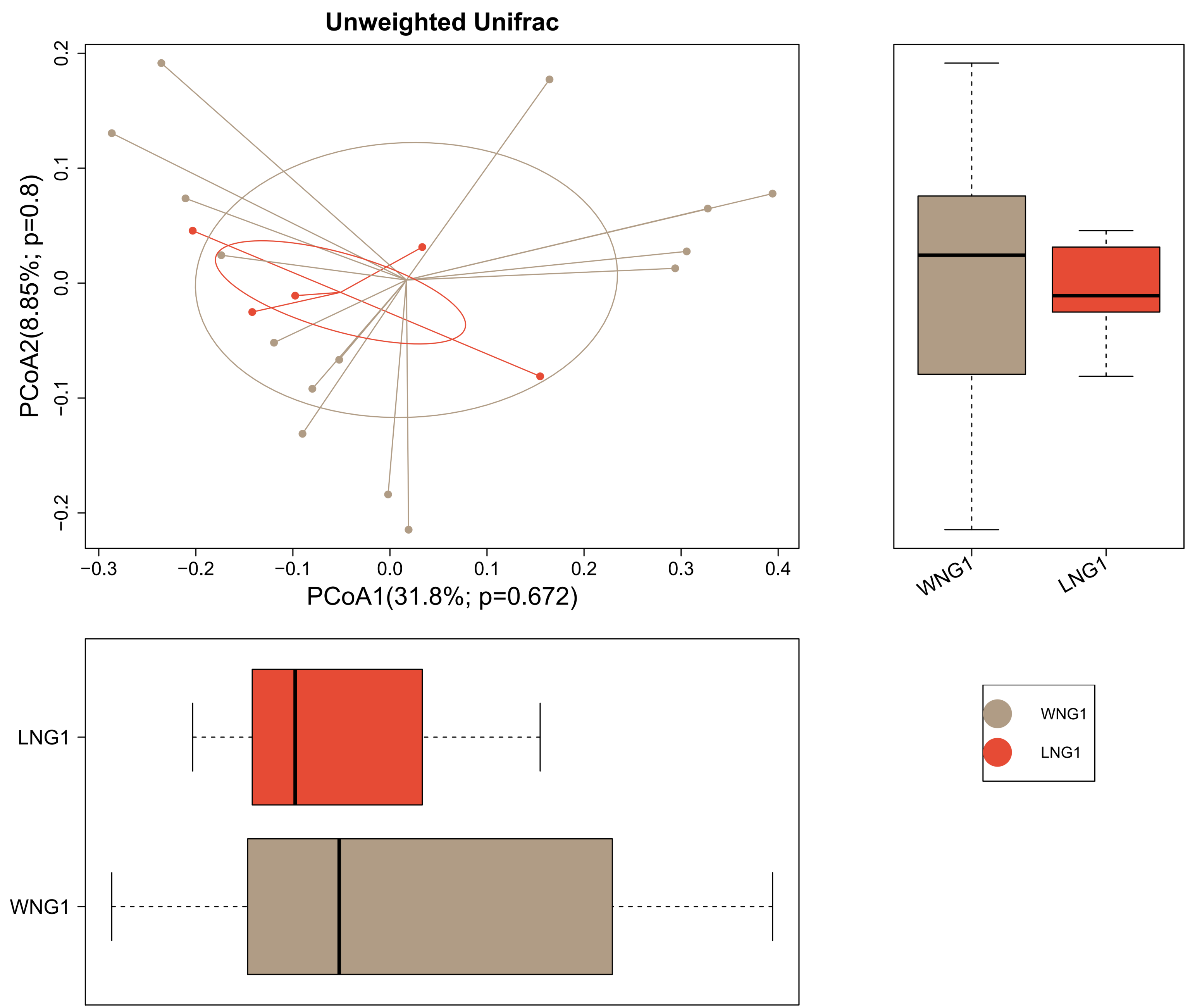
Unweighted Unifrac
PCoA2(8.85%; p=0.8)
PCoA1(31.8%; p=0.672)
0.2
0.1
0.0
−0.1
−0.2
−0.3
−0.2
−0.1
0.0
0.1
0.2
0.3
0.4
WNG1
LNG1
LNG1
WNG1
WNG1
LNG1

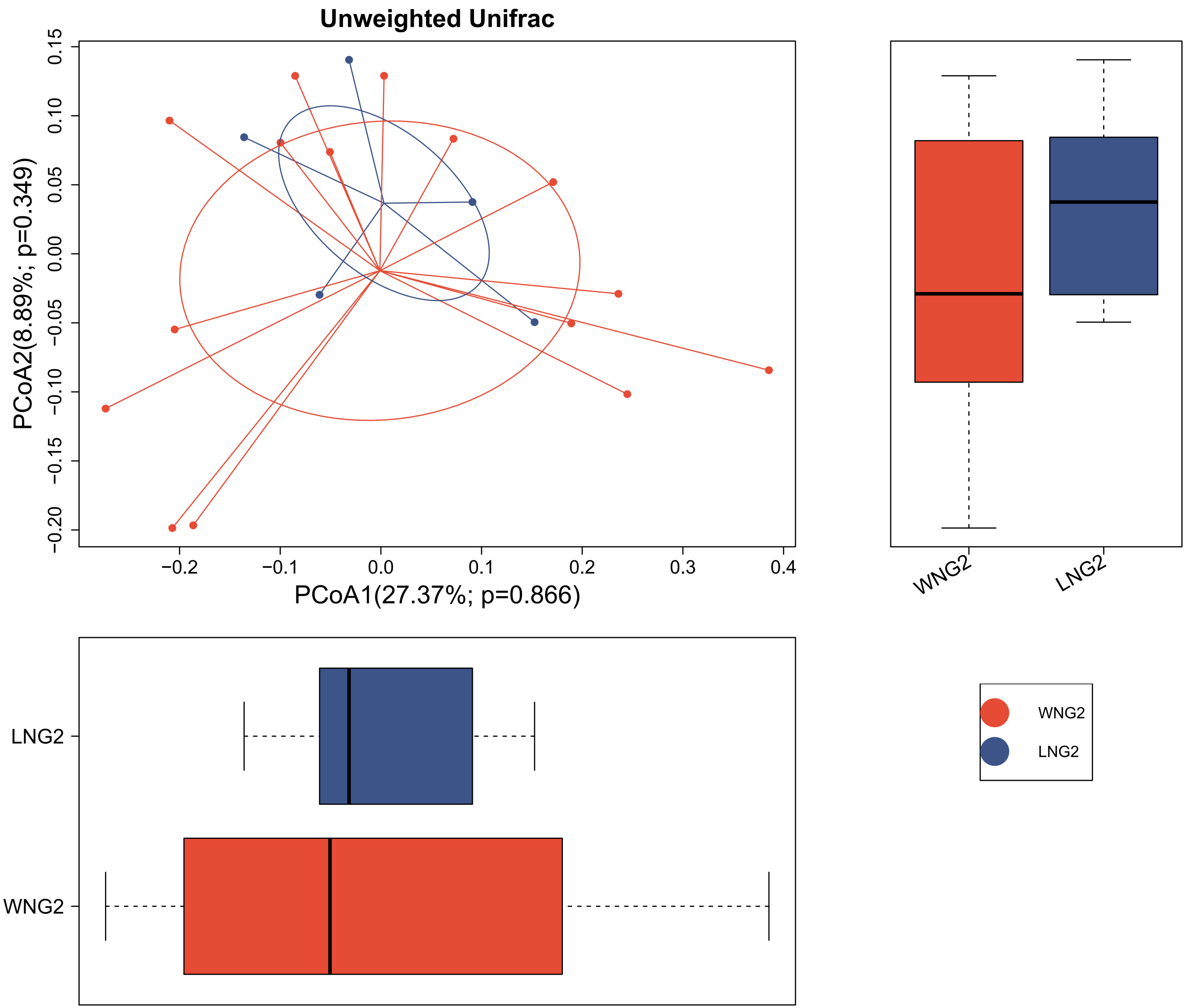

Unweighted Unifrac
PCoA2(8.89%; p=0.349)
PCoA1(27.37%; p=0.866)
0.15
0.10
0.05
0.00
−0.05
−0.10
−0.15
−0.20
−0.2
−0.1
0.0
0.1
0.2
0.3
0.4
WNG2
LNG2
LNG2
WNG2
WNG2
LNG2

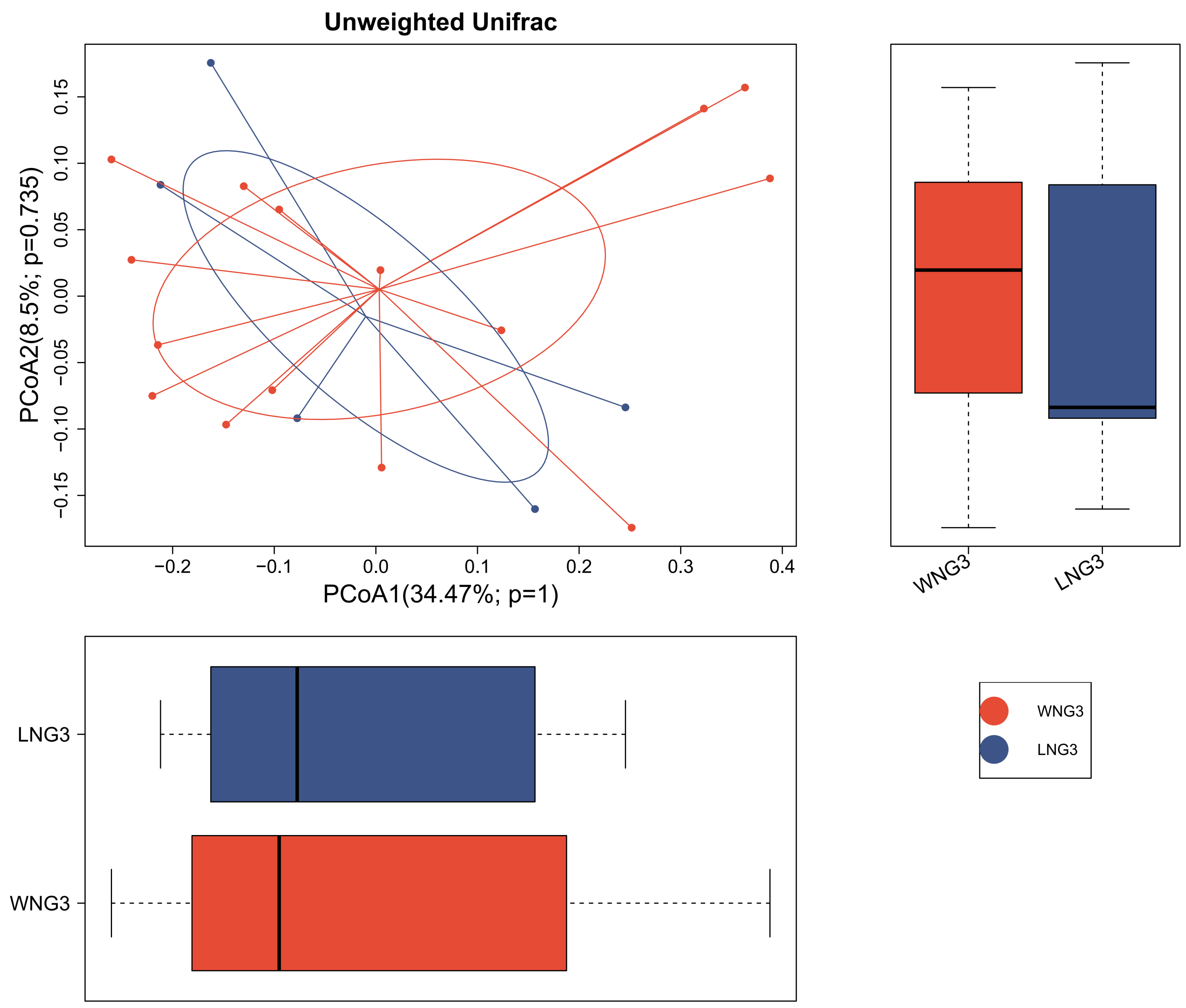
Unweighted Unifrac
PCoA1(34.47%; p=1)
PCoA2(8.5%; p=0.735)
−0.2
−0.1
0.0
0.1
0.2
0.3
0.4
−0.15
−0.10
−0.05
0.00
0.05
0.10
0.15
WNG3
LNG3
WNG3
LNG3
LNG3
WNG3
WNG3
LNG3

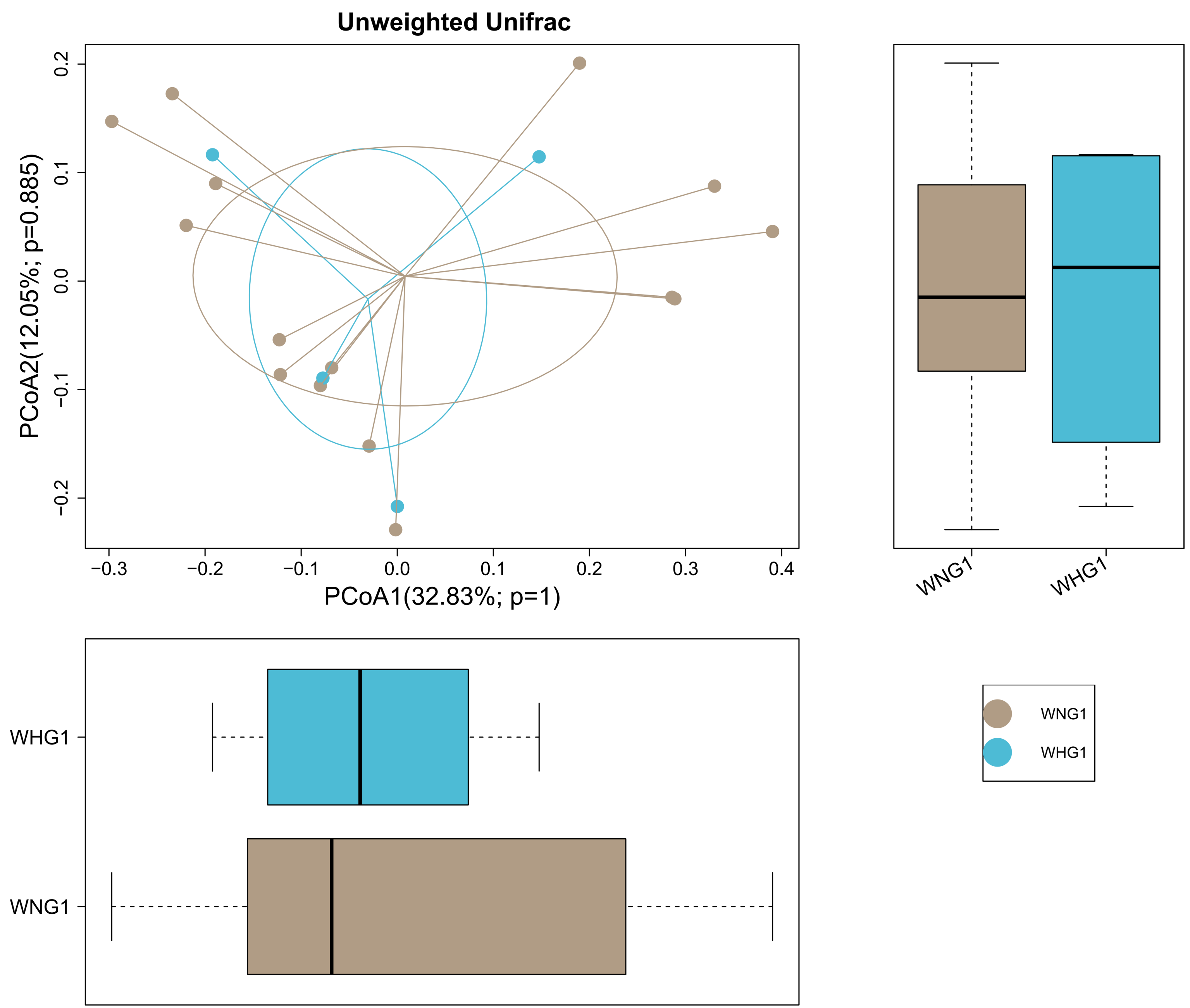
Unweighted Unifrac
PCoA1(32.83%; p=1)
PCoA2(12.05%; p=0.885)
−0.3
−0.2
−0.1
0.0
0.1
0.2
0.3
0.4
WNG1
WHG1

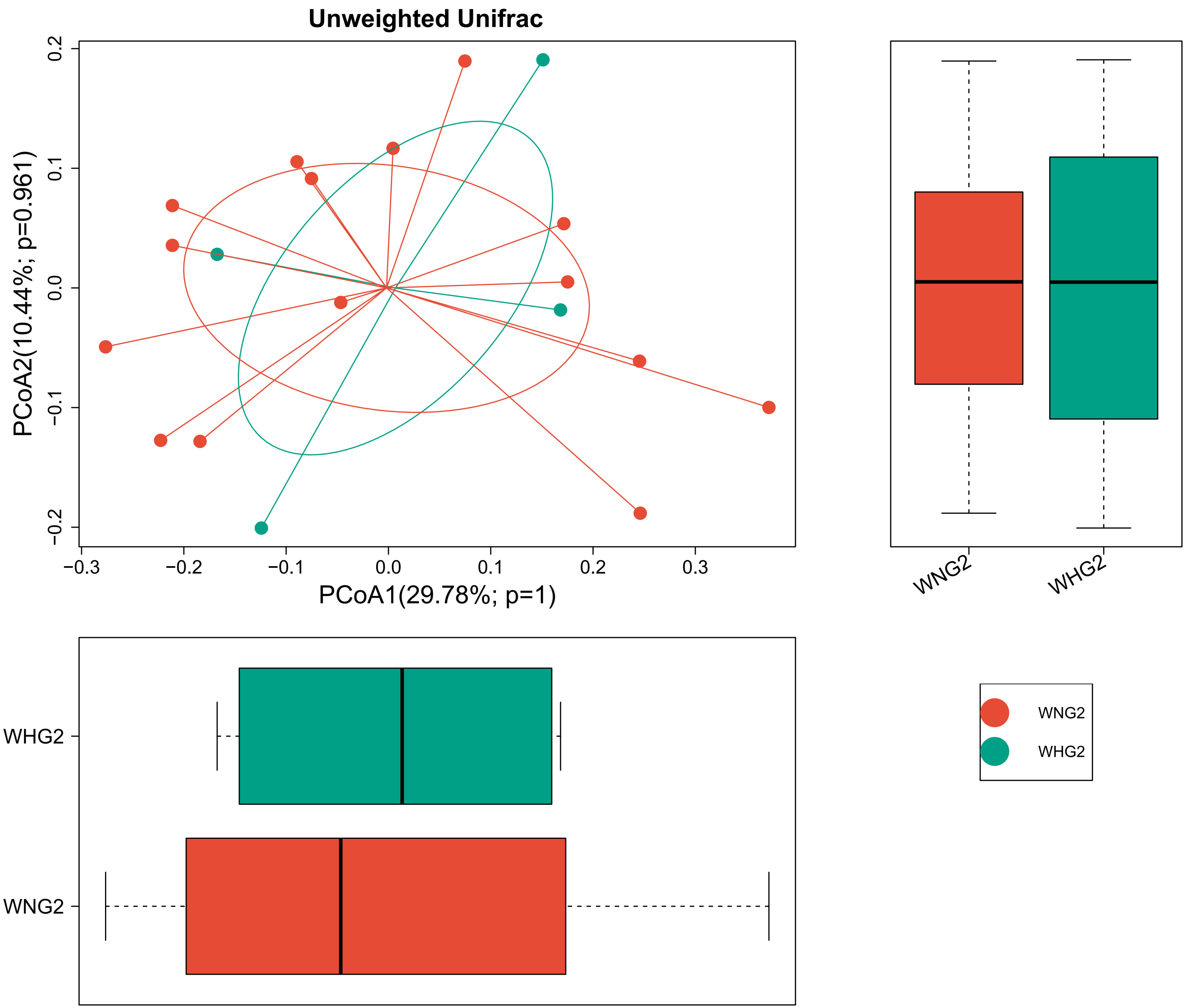
Unweighted Unifrac
PCoA2(10.44%; p=0.961)
PCoA1(29.78%; p=1)
0.2
0.1
0.0
−0.1
−0.2
−0.3
−0.2
−0.1
0.0
0.1
0.2
0.3
WNG2
WHG2
WHG2
WNG2
WNG2
WHG2

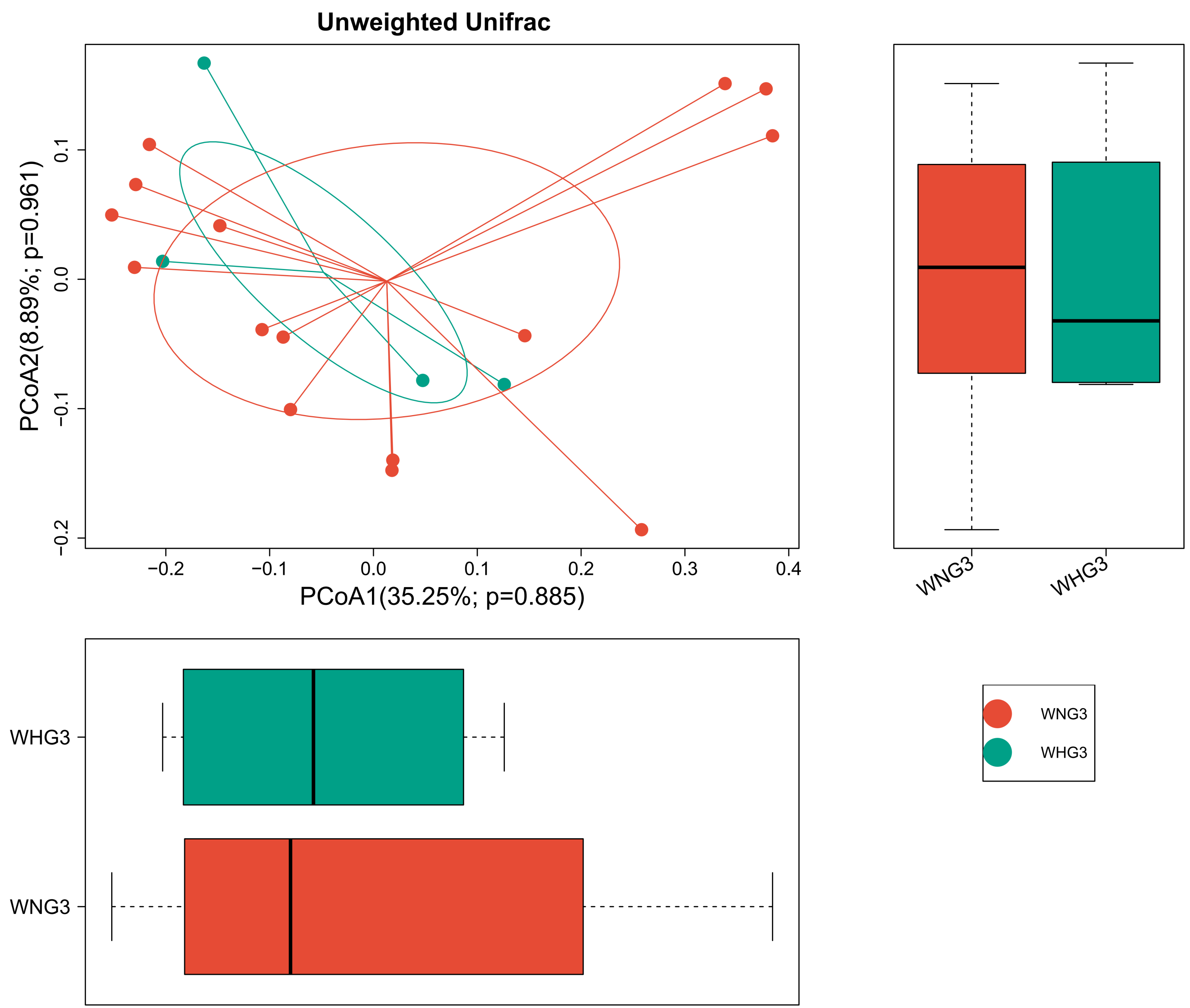

Unweighted Unifrac
PCoA2(8.89%; p=0.961)
PCoA1(35.25%; p=0.885)
0.1
0.0
−0.1
−0.2
−0.2
−0.1
0.0
0.1
0.2
0.3
0.4
WNG3
WHG3
WHG3
WNG3
WNG3
WHG3

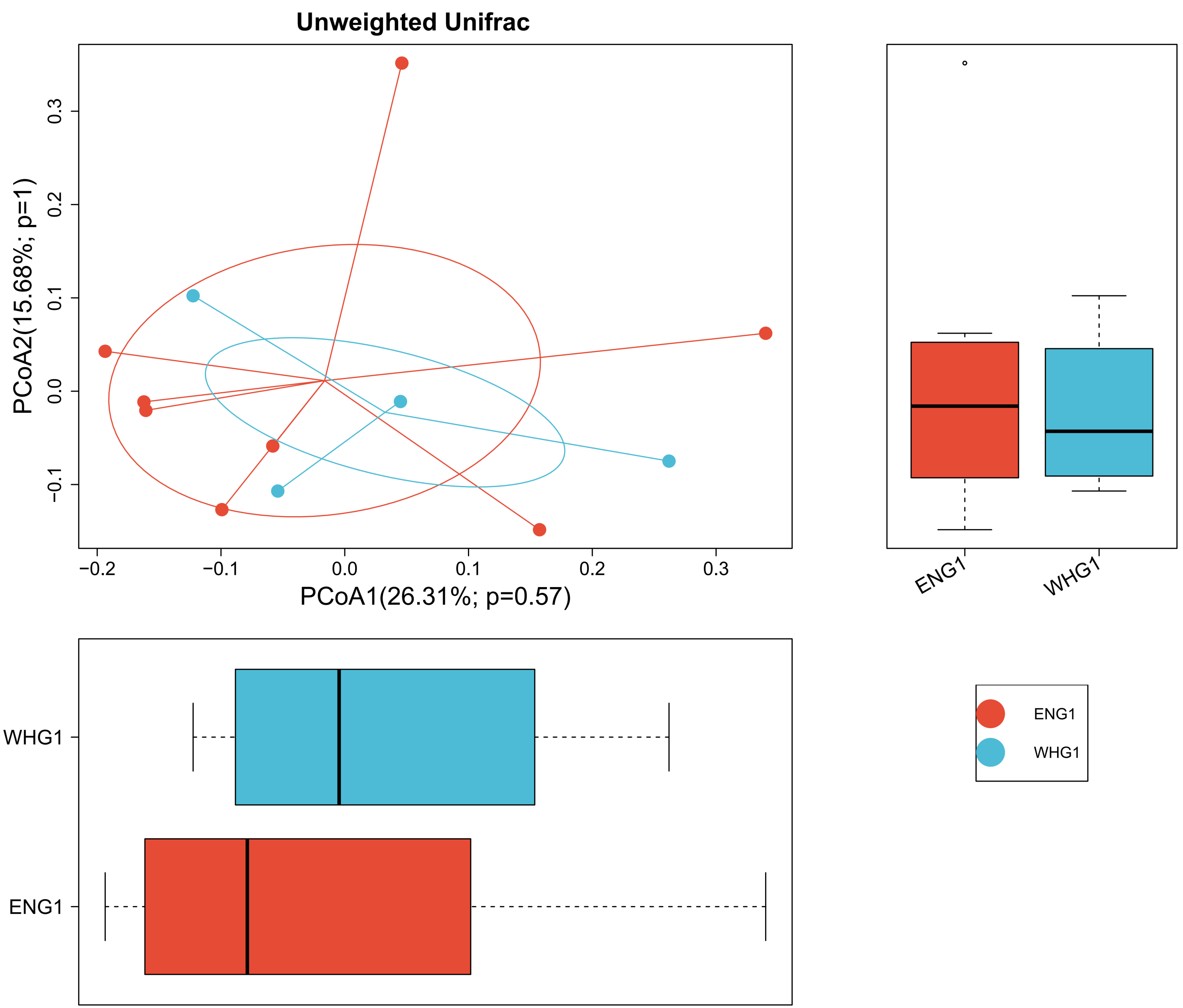

Unweighted Unifrac
PCoA2(15.68%; p=1)
PCoA1(26.31%; p=0.57)
0.3
0.2
0.1
0.0
−0.1
−0.2
−0.1
0.0
0.1
0.2
0.3
ENG1
WHG1
WHG1
ENG1
ENG1
WHG1

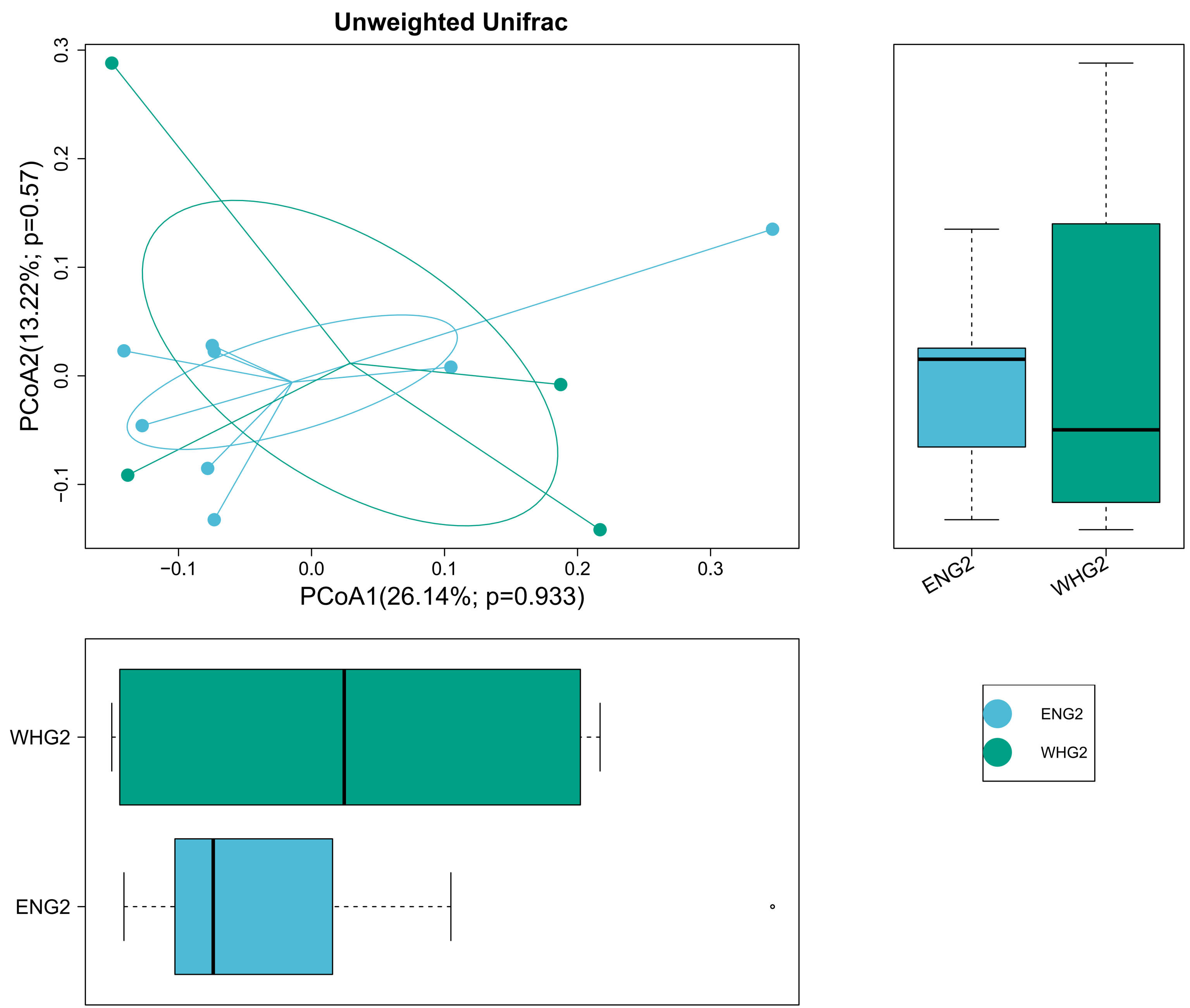

Unweighted Unifrac
PCoA1(26.14%; p=0.933)
PCoA2(13.22%; p=0.57)
−0.1
0.0
0.1
0.2
0.3
ENG2
WHG2

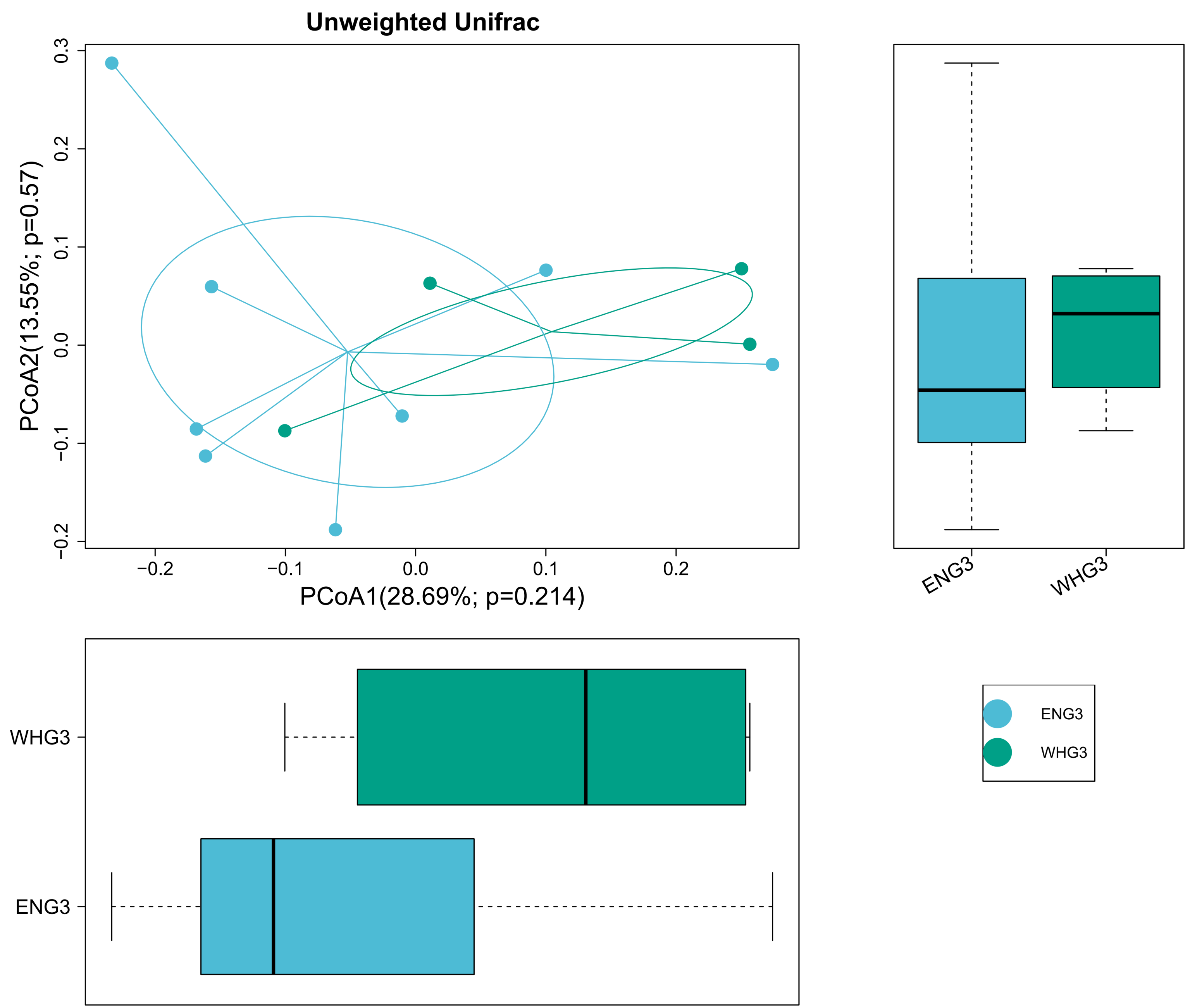

Unweighted Unifrac
PCoA2(13.55%; p=0.57)
PCoA1(28.69%; p=0.214)
0.3
0.2
0.1
0.0
−0.1
−0.2
−0.2
−0.1
0.0
0.1
0.2
ENG3
WHG3
WHG3
ENG3
ENG3
WHG3

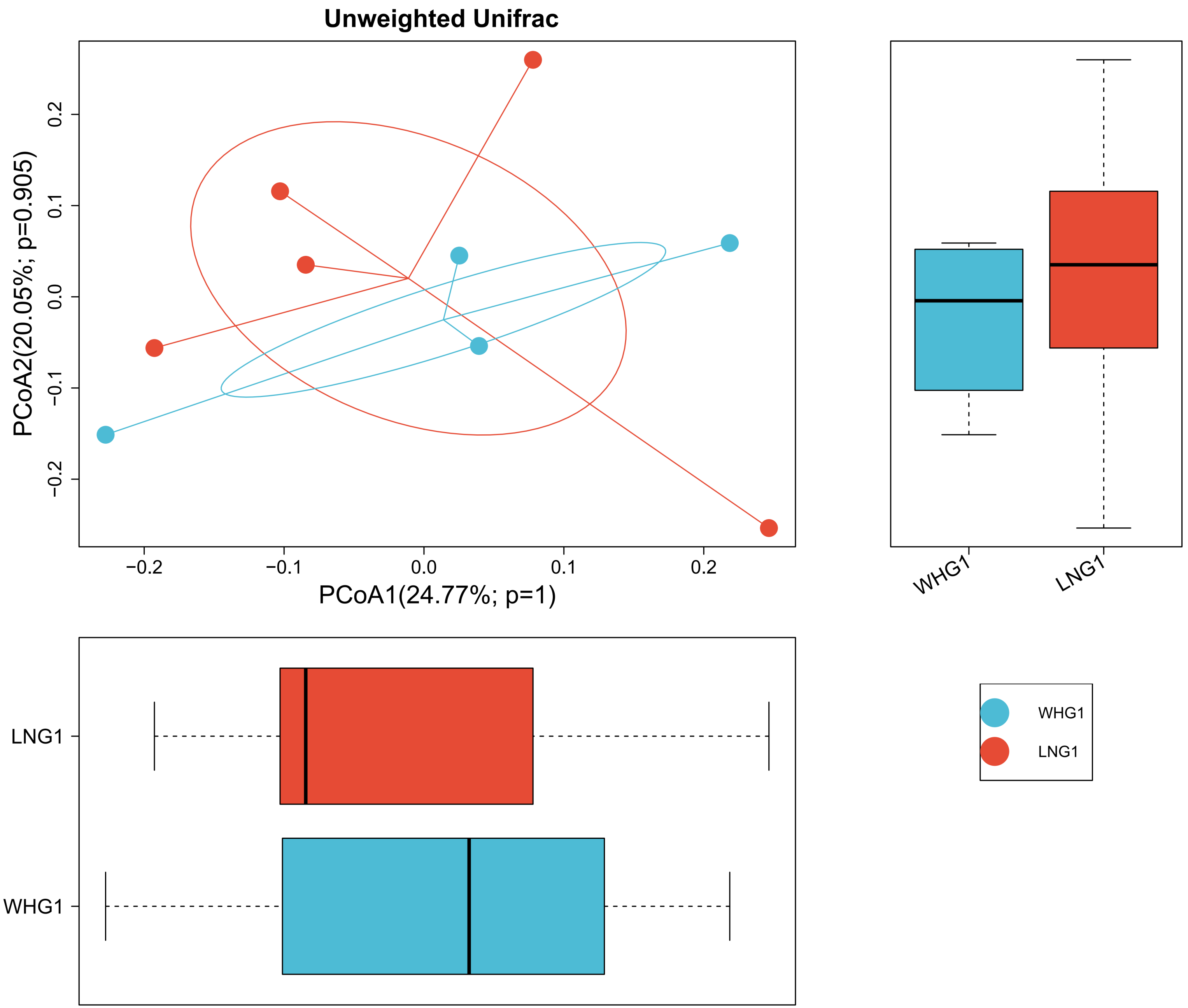

Unweighted Unifrac
PCoA2(20.05%; p=0.905)
0.2
0.1
0.0
−0.1
−0.2
−0.2
−0.1
0.0
0.1
0.2
PCoA1(24.77%; p=1)
WHG1
LNG1
LNG1
WHG1
WHG1
LNG1

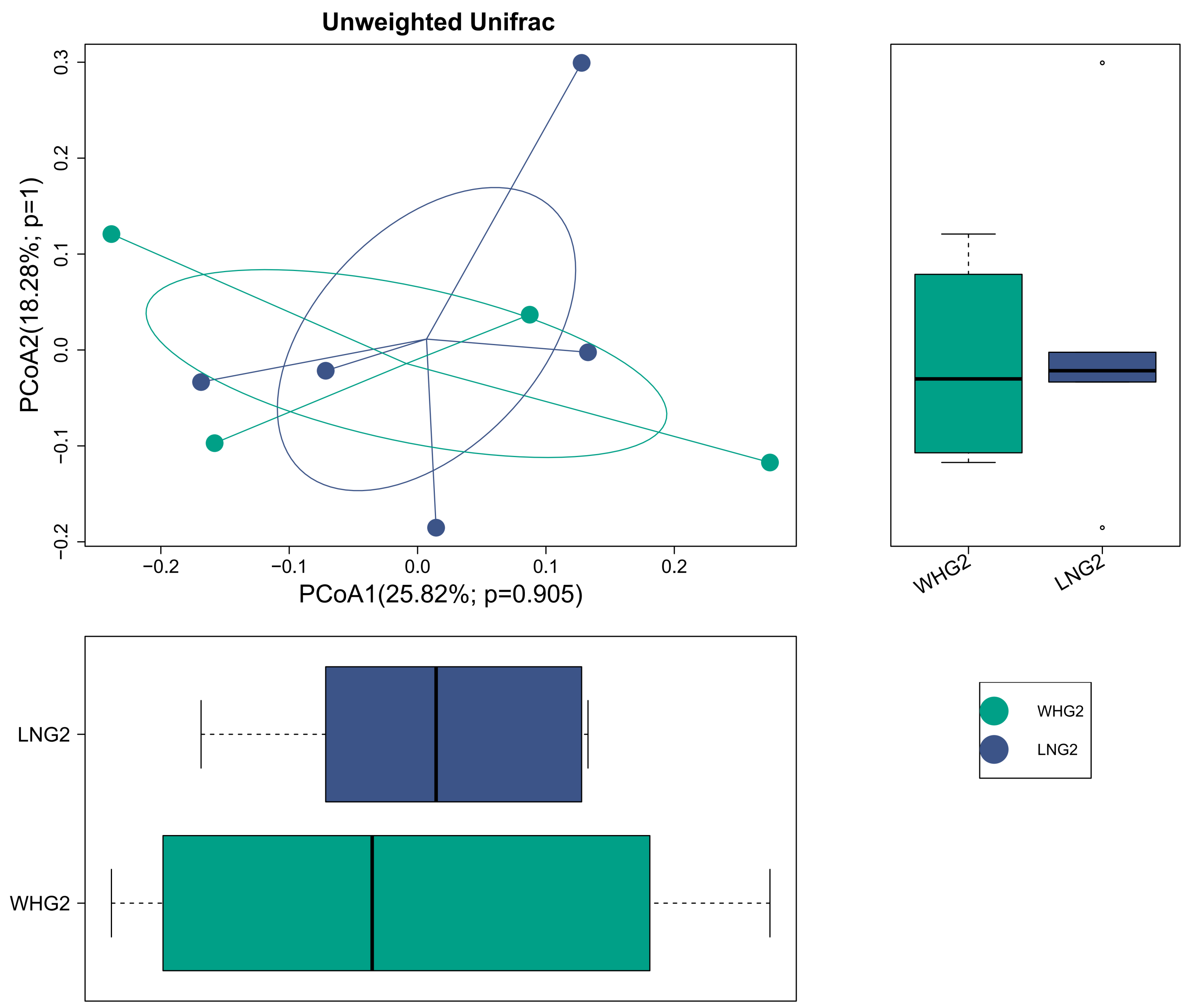

Unweighted Unifrac
PCoA2(18.28%; p=1)
0.3
0.2
0.1
0.0
−0.1
−0.2
−0.2
−0.1
0.0
0.1
0.2
PCoA1(25.82%; p=0.905)
WHG2
LNG2
LNG2
WHG2
WHG2
LNG2

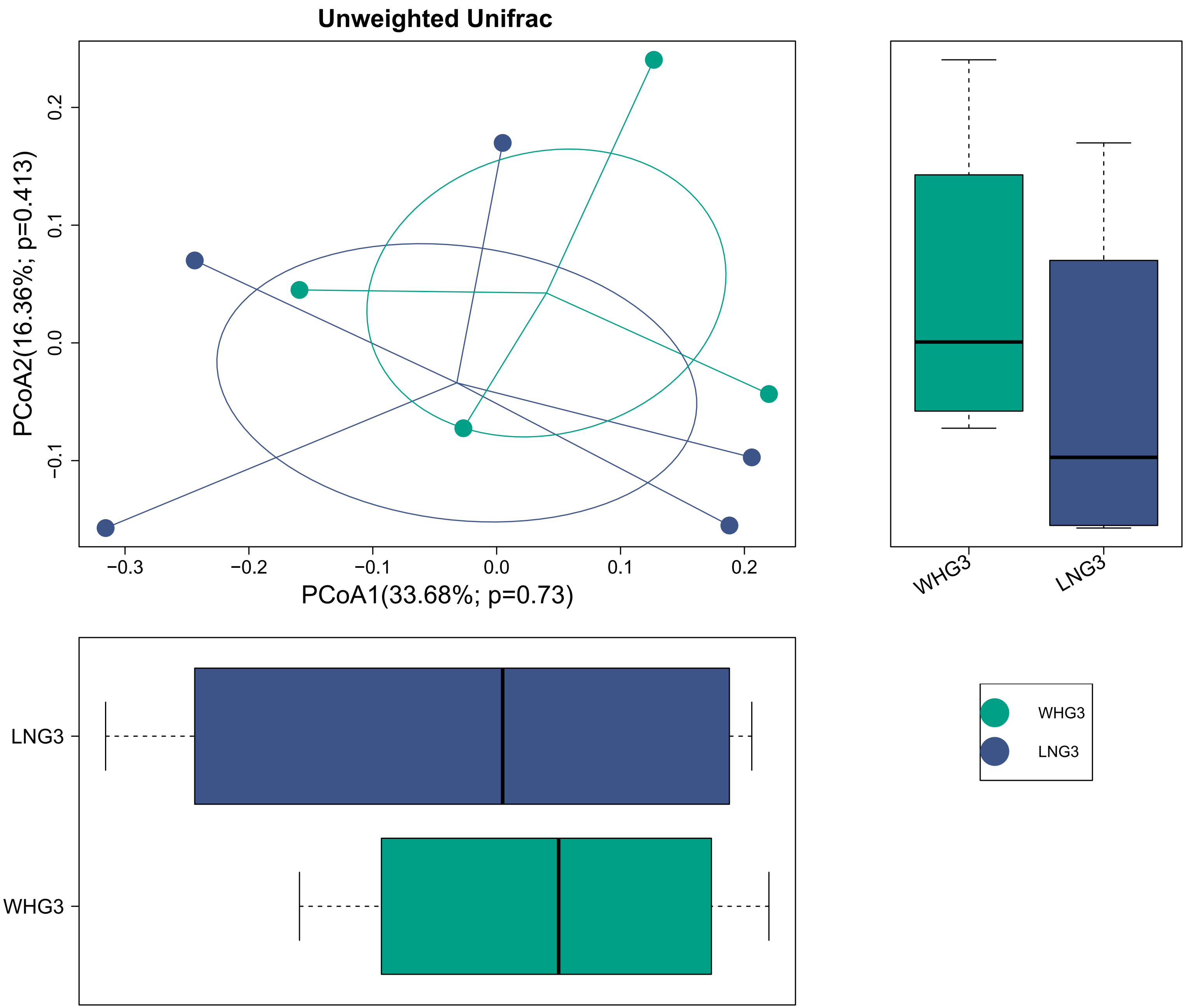

Unweighted Unifrac
PCoA2(16.36%; p=0.413)
PCoA1(33.68%; p=0.73)
0.2
0.1
0.0
−0.1
−0.3
−0.2
−0.1
0.0
0.1
0.2
WHG3
LNG3
LNG3
WHG3
WHG3
LNG3
