# Supplementary material for: Dynamic changes in the gut microbiota during three consecutive trimesters of pregnancy and their correlation with abnormal glucose and lipid metabolism
Source: Eur J Med Res. 2024 Feb 12;29:117. doi: 10.1186/s40001-024-01702-0 (PMC10860297; doi:10.1186/s40001-024-01702-0)
Supplement: Supplementary file 5 — Additional file 5. Comparisons of the Chao1 index alpha diversity of the gut microbiota in those groups with different onset time of dyslipidemia across the three trimesters. There were no significant differences in the alpha diversity of the gut microbiota (GM) between DL1 and DL2 (P1–3), DL1 and DL3(P4–6), DL2 and DL3 (P7–9) in T1, T2 and T3. P value is shown in each chart. T1: the first trimester; T2: the second trimester; T3: the third trimester; DL3: with dyslipidemia only in T3, DL2: with dyslipidemia since T2, DL1: dyslipidemia starting from T1 of pregnancy. [file 40001_2024_1702_MOESM5_ESM.pdf]

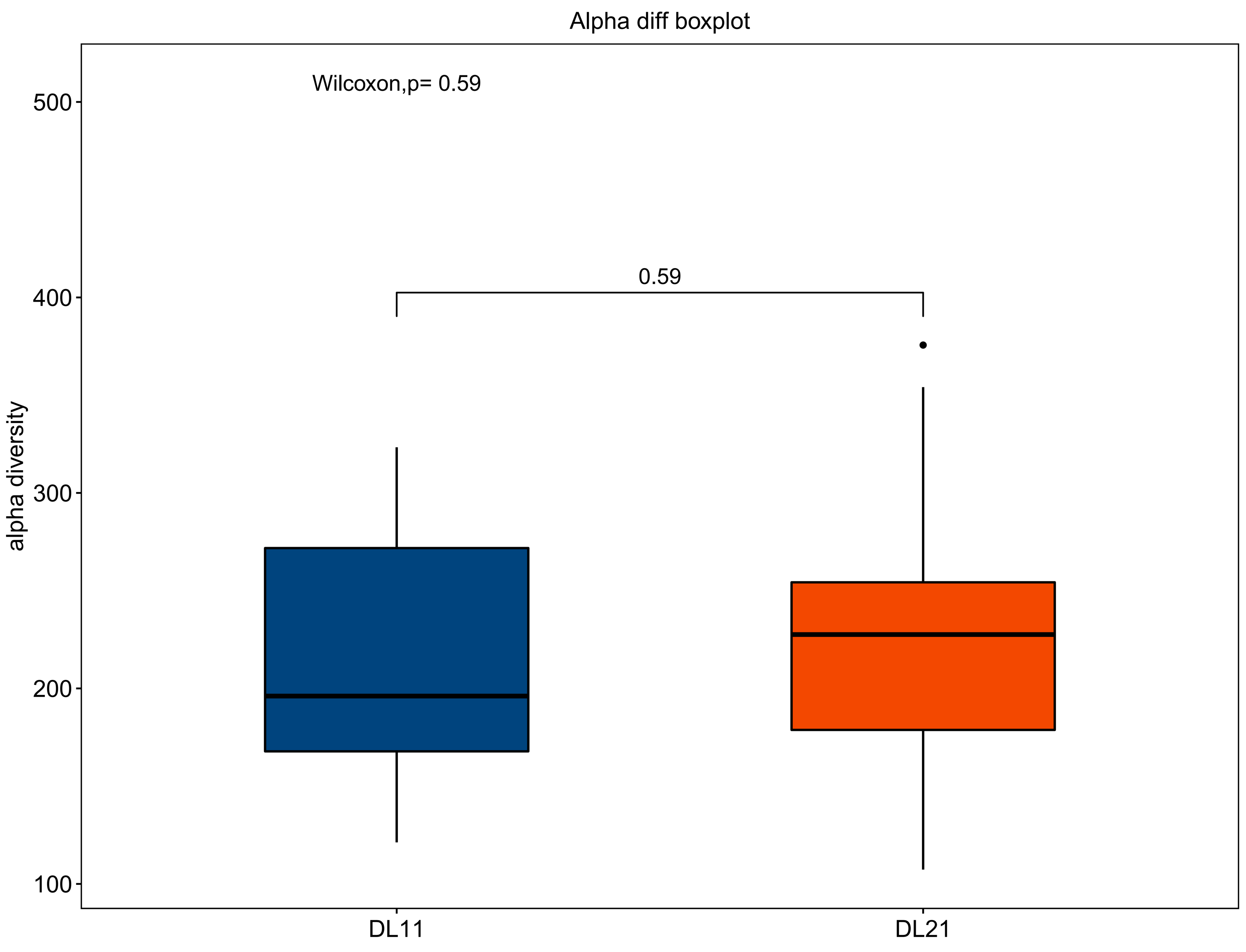
Alpha diff boxplot
Wilcoxon,p= 0.59
0.59
alpha diversity
500
400
300
200
100
DL11
DL21

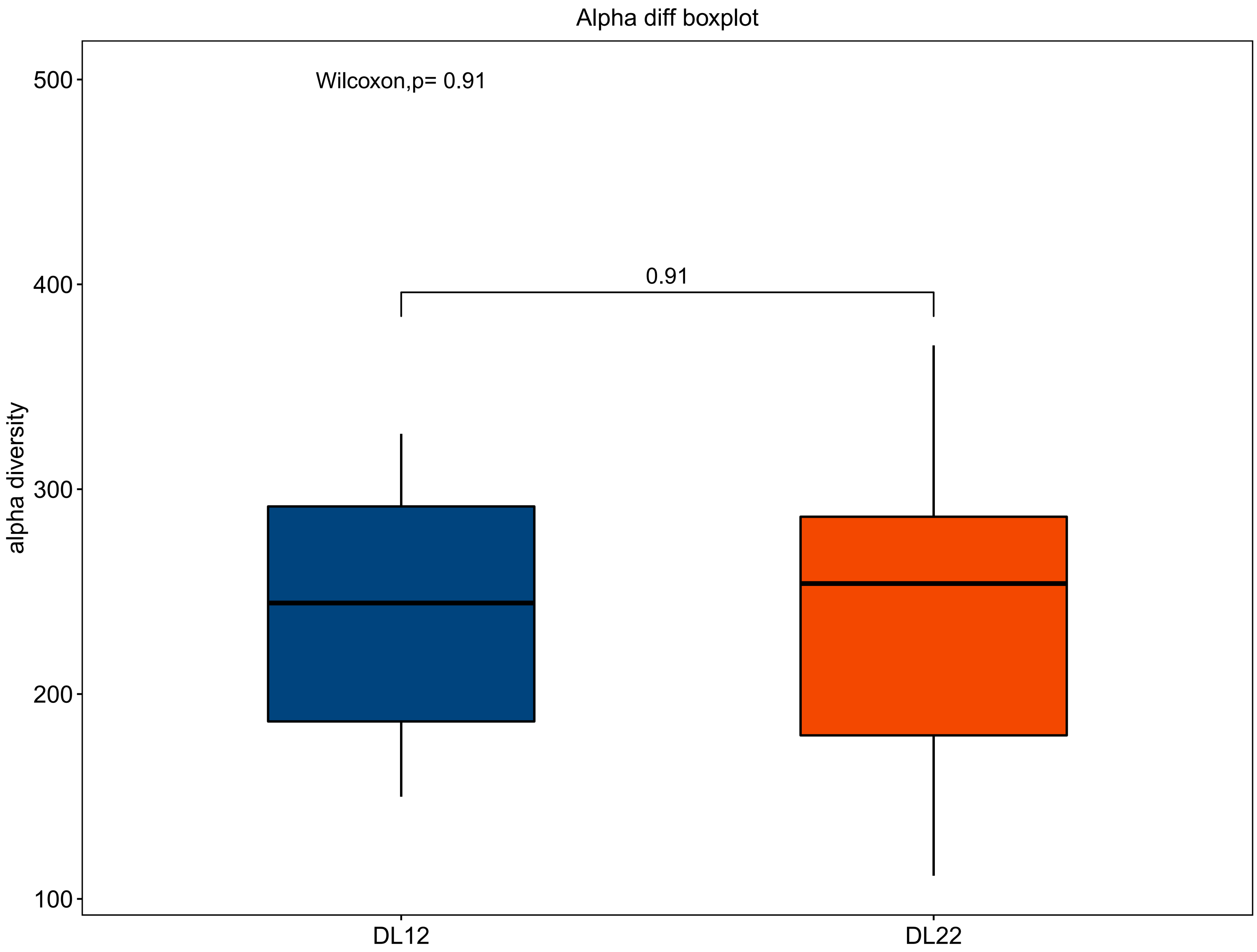
Alpha diff boxplot
Wilcoxon,p= 0.91
0.91
alpha diversity
500
400
300
200
100
DL12
DL22

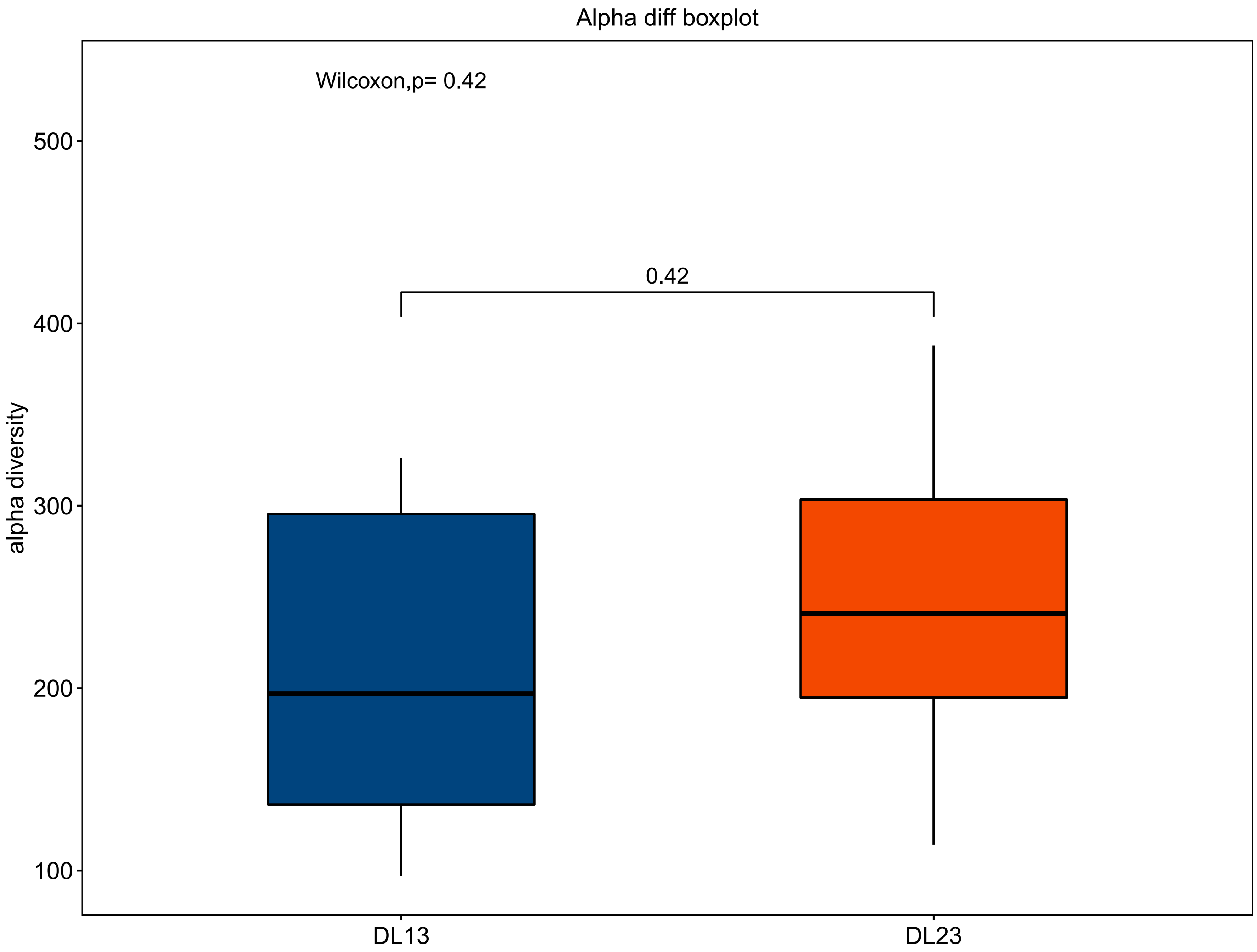

Alpha diff boxplot
Wilcoxon,p= 0.42
0.42
alpha diversity
500
400
300
200
100
DL13
DL23

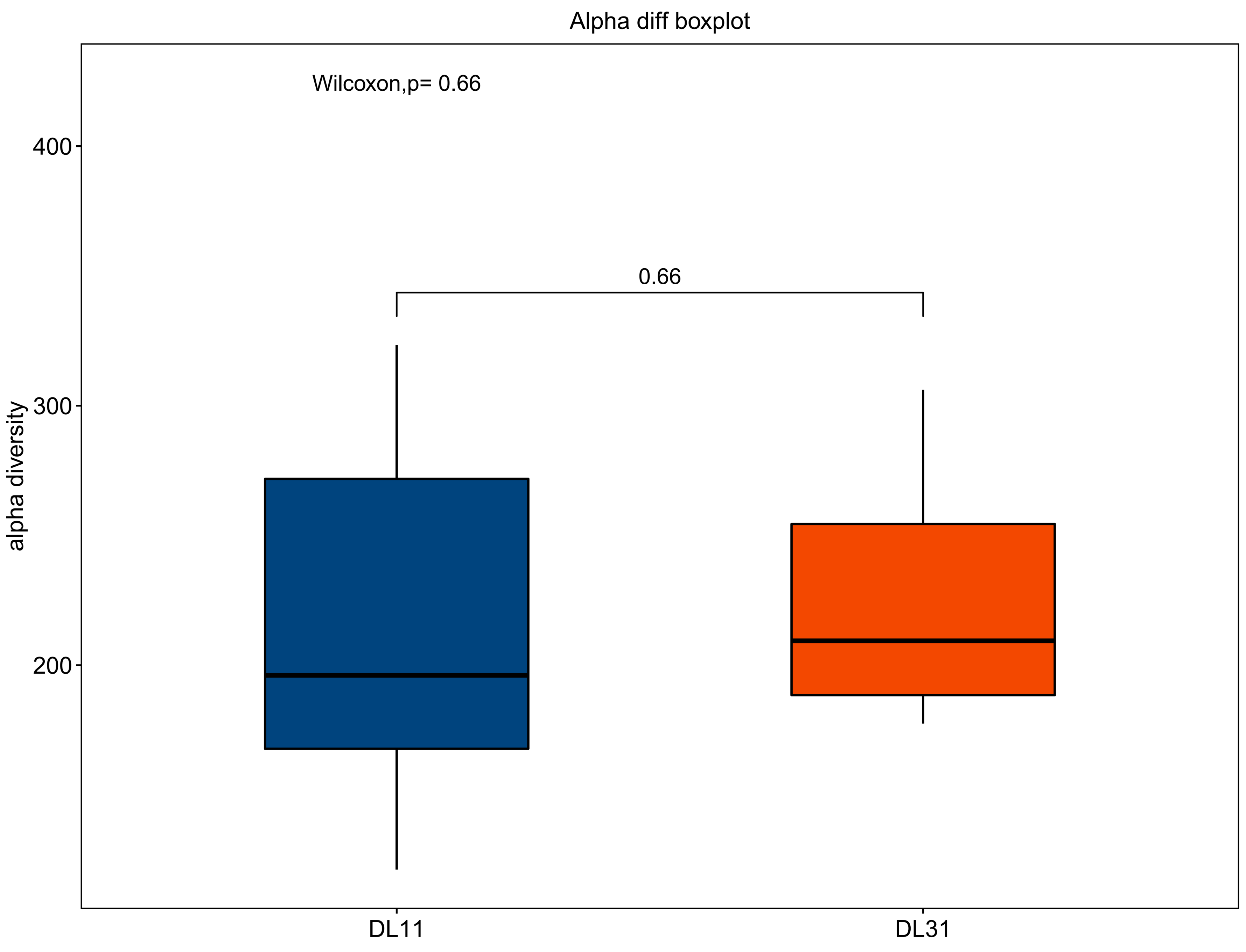
Alpha diff boxplot
Wilcoxon,p= 0.66
0.66
alpha diversity
400
300
200
DL11
DL31

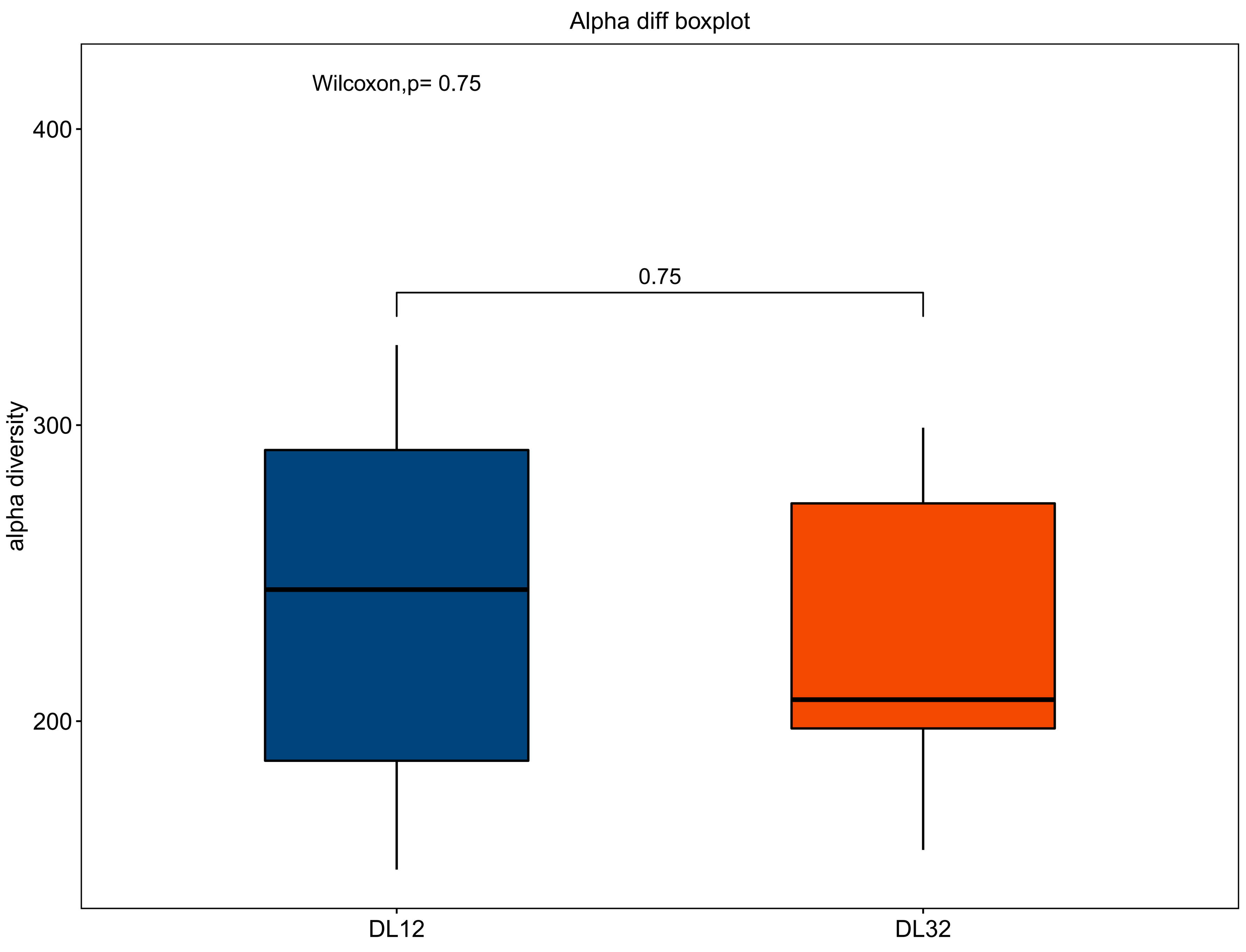

Alpha diff boxplot
Wilcoxon,p= 0.75
0.75
alpha diversity
400
300
200
DL12
DL32

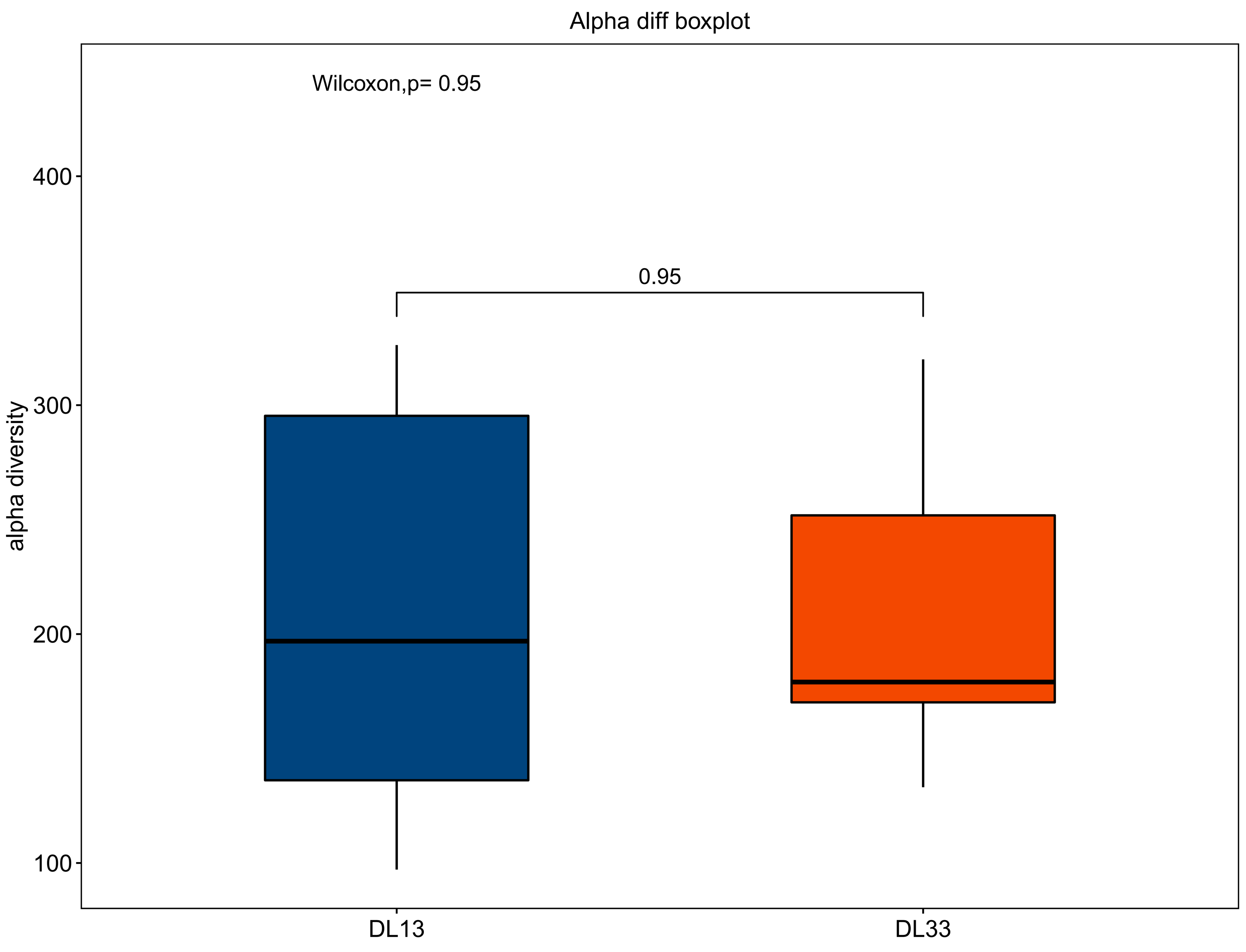

Alpha diff boxplot
Wilcoxon,p= 0.95
0.95
alpha diversity
400
300
200
100
DL13
DL33

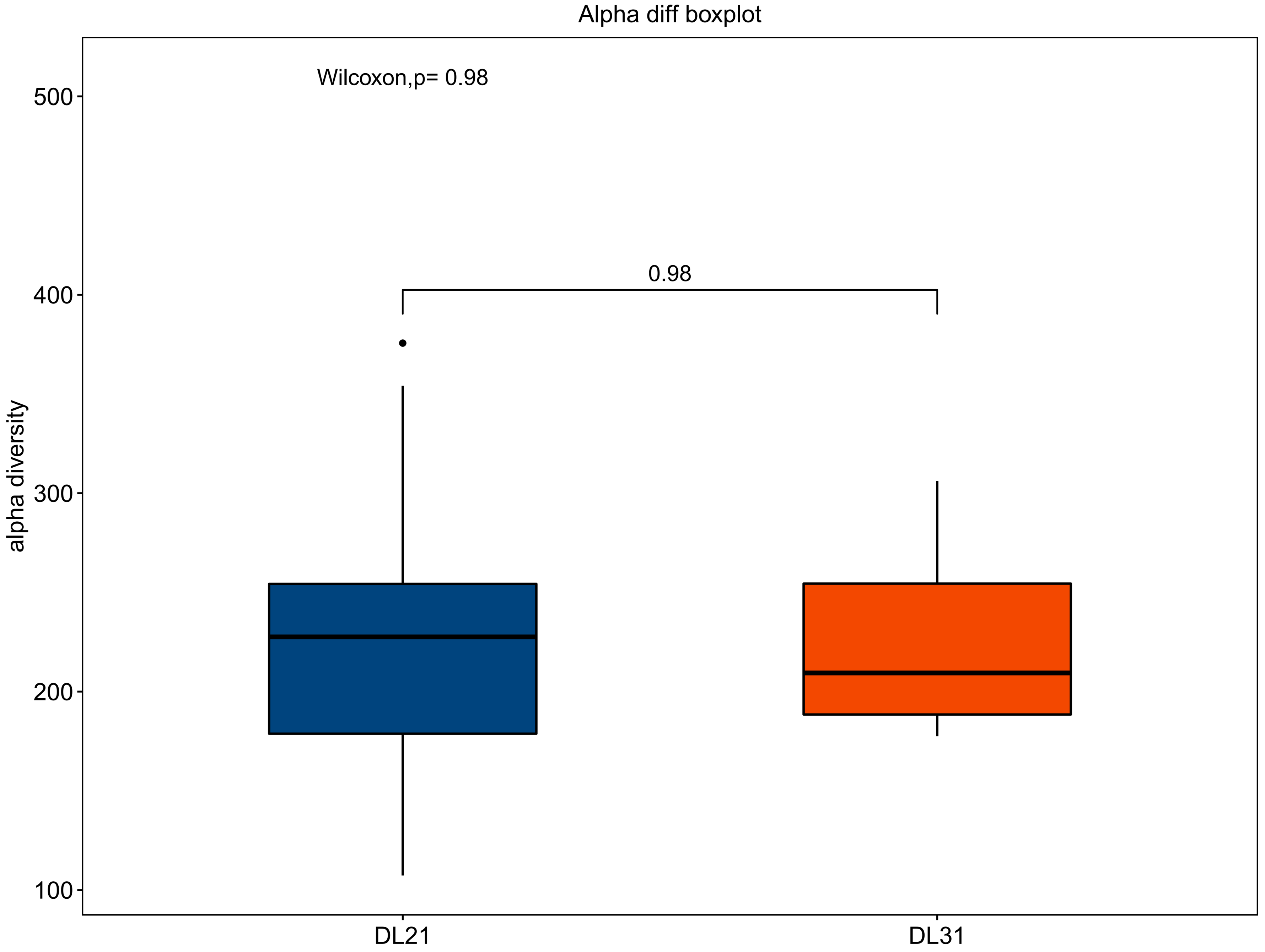
Alpha diff boxplot
Wilcoxon,p= 0.98
0.98
500
400
300
200
100
alpha diversity
DL21
DL31

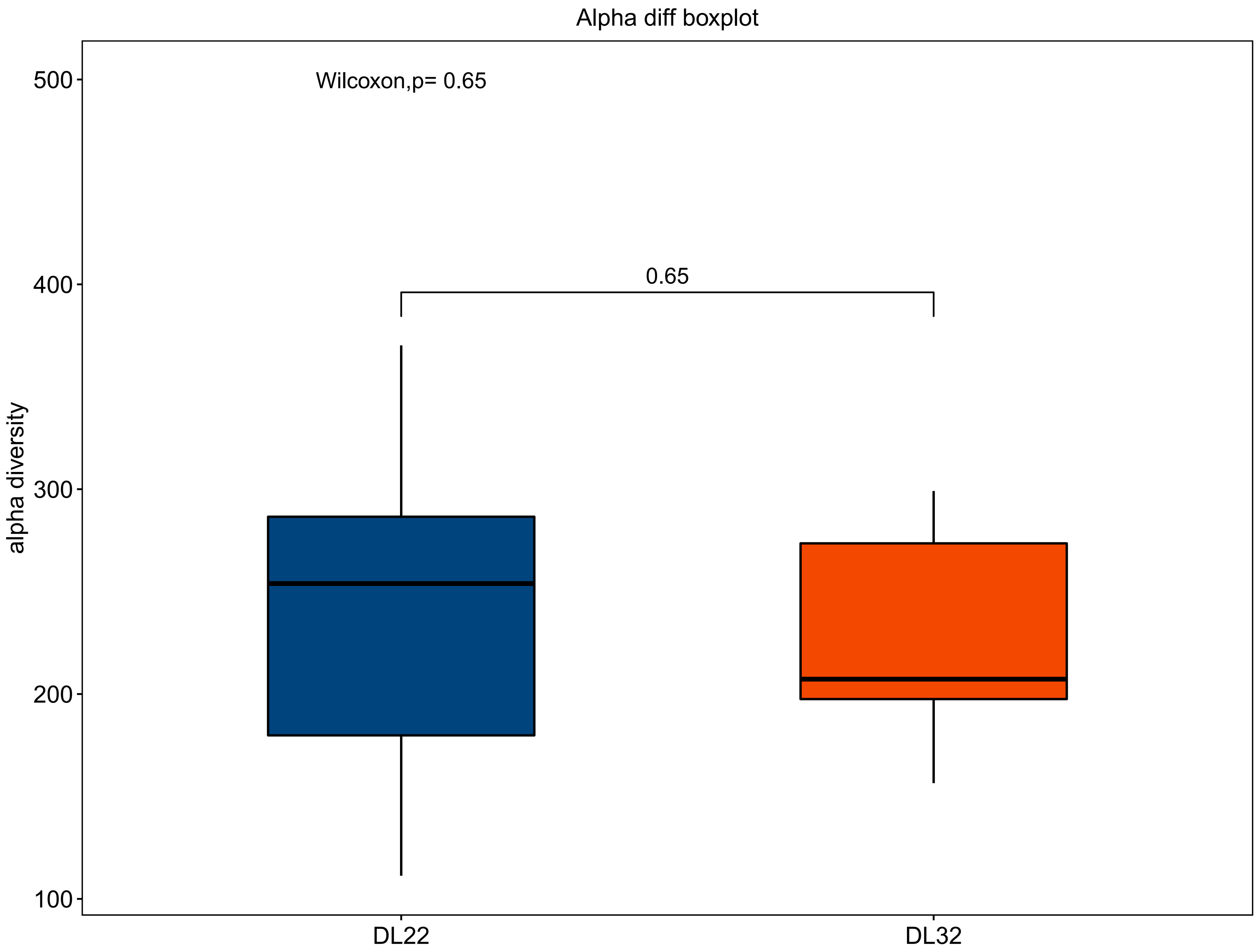

Alpha diff boxplot
Wilcoxon,p= 0.65
0.65
alpha diversity
500
400
300
200
100
DL22
DL32

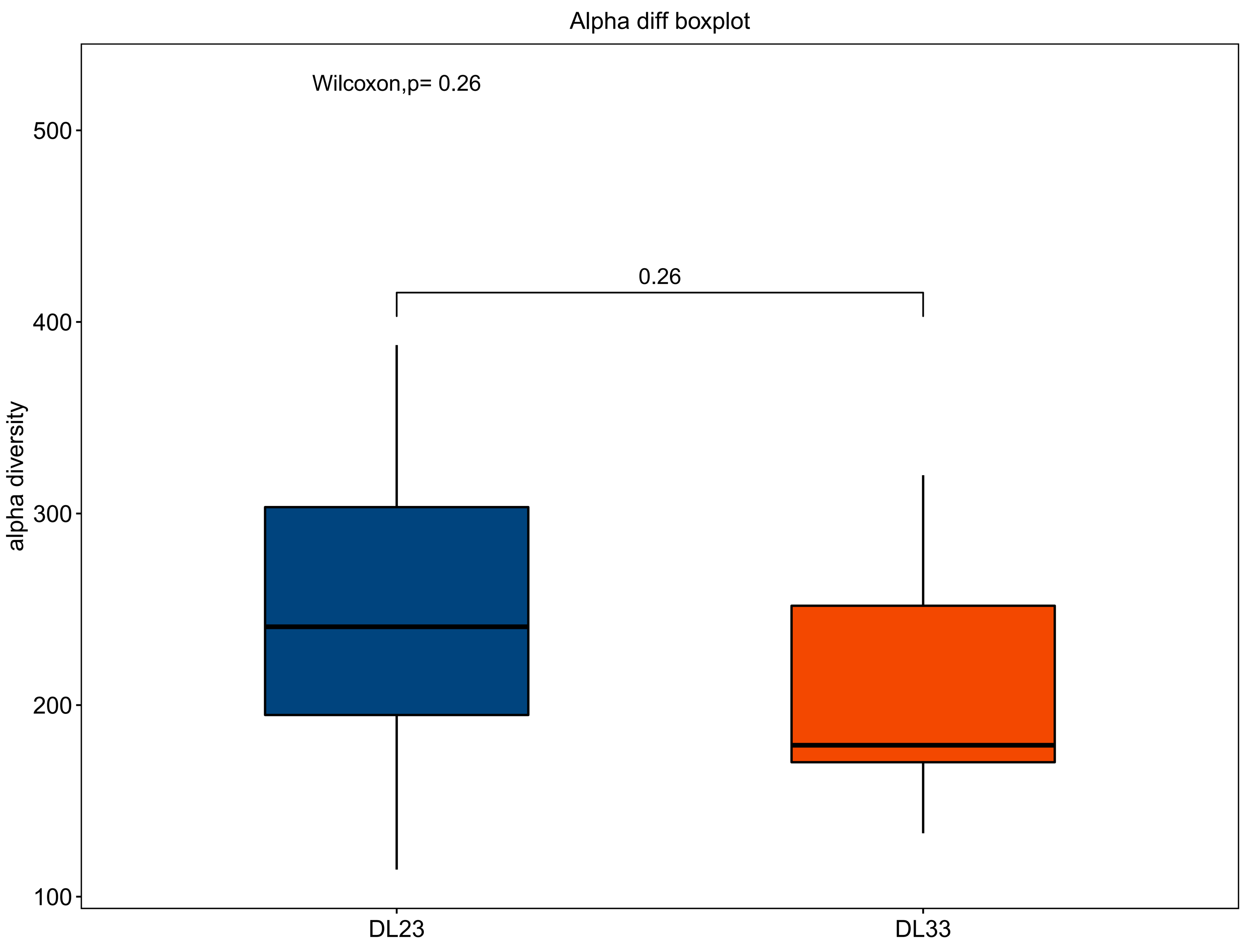

Alpha diff boxplot
Wilcoxon,p= 0.26
0.26
alpha diversity
500
400
300
200
100
DL23
DL33
